# Discovery of multi-target receptor tyrosine kinase inhibitors as novel anti-angiogenesis agents

**DOI:** 10.1038/srep45145

**Published:** 2017-03-23

**Authors:** Jinfeng Wang, Lin Zhang, Xiaoyan Pan, Bingling Dai, Ying Sun, Chuansheng Li, Jie Zhang

**Affiliations:** 1 School of Pharmacy, Health Science Center, Xi’an Jiaotong University, No. 76, Yanta West Road, Xi’an, 710061, P.R. China

## Abstract

Recently, we have identified a biphenyl-aryl urea incorporated with salicylaldoxime (**BPS-7**) as an anti-angiogenesis agent. Herein, we disclosed a series of novel anti-angiogenesis agents with **BPS-7** as lead compound through combining diarylureas with *N*-pyridin-2-ylcyclopropane carboxamide. Several title compounds exhibited simultaneous inhibition effects against three pro-angiogenic RTKs (VEGFR-2, TIE-2 and EphB4). Some of them displayed potent anti-proliferative activity against human vascular endothelial cell (EA.hy926). In particular, two potent compounds (CDAU-1 and CDAU-2) could be considered as promising anti-angiogenesis agents with triplet inhibition profile. The biological evaluation and molecular docking results indicate that *N*-pyridin-2-ylcyclopropane carboxamide could serve as a hinge-binding group (HBG) for the discovery of multi-target anti-angiogenesis agents. CDAU-2 also exhibited promising anti-angiogenic potency in a tissue model for angiogenesis.

Pathological angiogenesis plays a critical role in numerous diseases including cancer, rheumatoid arthritis, and retinopathies^1^. Recently, many efforts have been focused on the discovery of novel anti-angiogenesis agents for the treatment of cancer, ocular, joint or skin disorders^2^,^3^. Receptor tyrosine kinases (RTKs) display a variety of biological activities including cell proliferation and migration. Multiple RTKs are involved in pathological angiogenesis and have been identified as valid targets for developing anti-angiogenesis agents. Inhibition of angiogenic RTKs has been also considered as a systemic strategy for tumor treatment. Vascular endothelial growth factor (VEGF) is one of the most potent pro-angiogenic factors, and its binding to the receptors (VEGFRs) promotes endothelial cell survival, proliferation, and migration^4^. VEGFR families mainly consists of VEGFR-1 (Flt-1), VEGFR-2 (KDR/Flk-1), and VEGFR-3 (Flt-4)^5^,^6^. Among all of them, VEGFR-2 plays an essential role in tumor angiogenesis^7^. Likewise, angiogenic growth factors (Ang) and their tyrosine kinase with Ig and EGF homology domain-2 (TIE-2) have been implicated in sprouting, branching, remodeling and maturation of the blood vessels^8^,^9^,^10^. Recent research indicated that erythropoietin producing hepatocyte receptor B4 (EphB4) and its transmembrane-type ligand (ephrin B2) were crucial for angiogenesis, vessel maturation, and pericyte recruitment^11^. EphB4 belongs to the Eph family which is the largest subfamily of RTKs^12^. Meaningfully, EphB4-ephrinB2 signaling pathway play critical roles in tumor vessel development and maturation^13^,^14^. Tumor growth is dependent on multiple factors, including the physiological process of angiogenesis. It has been demonstrated that tumors are capable of secreting multiple angiogenic growth factors. Inhibiting angiogenesis have been identified as a new approach for inhibition of tumor growth, and its clinical efficacy are being evaluated now. Through this approach, some promising small-molecule inhibitors towards various angiogenic RTKs including VEGFR-2, FGFR, PDGFR, TIE-2 and EphB4 are reported. However, it has been also demonstrated that inhibition of a single angiogenic pathway in angiogenesis could induce the acquired resistance. Maximizing the potential of anti-angiogenic therapy is likely to require a broader therapeutic approach using a new generation of multi-target anti-angiogenesis agents. We speculate that simultaneous inhibition of multiple RTKs might be a good strategy to address this issue and achieve maximal clinical efficacy. There are several small molecular multi-target RTK inhibitors in clinical trials as anti-angiogenesis agents. Four drugs with different inhibition profile against angiogenic RTKs have been approved including sorafenib^15^, linifanib^16^, vandetanib^17^, and cabozantinib^18^. Herein, we described here the first discovery of multiple inhibitors of VEGFR-2/EphB4/TIE-2 as novel anti-angiogenesis agents.

## Results

### Design strategy of multi-target inhibitors

**Figure 1:**
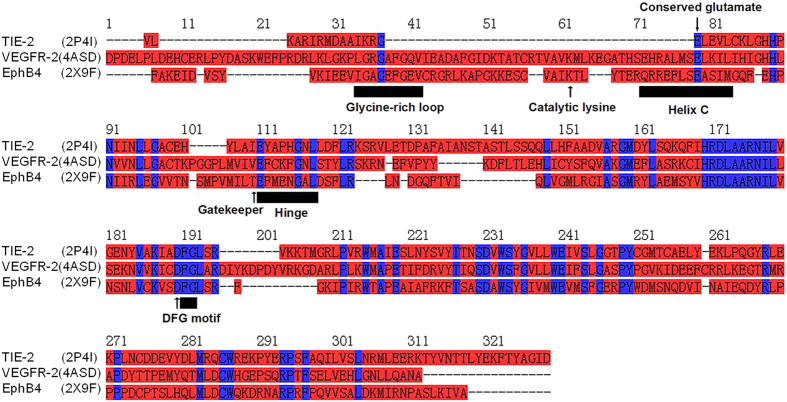
Sequence alignment of ATP-binding pocket of the three RTKs with PDB code and name (VEGFR-2, Tie-2, and EphB4; Conserved residues are colored blue).

With the determination of RTK crystal structures, it has been recognized that the primary sequence homology among various RTKs was apparent. The catalytic domains and ATP-binding site in the active conformation of these RTKs are notably similar. In addition, it is reasonable to assume that all the ATP-competitive RTK inhibitors with anti-angiogenesis potency share the same binding sites. Structural and sequence comparison of three RTKs (VEGFR-2, TIE-2, and EphB4) were depicted in Fig. 1. It is indicated that extensive sequence homology is evident along the kinase domain among VEGFR-2, TIE-2, and EphB4. Moreover, they share several short motifs including glycine-rich loop, conserved glutamate, gatekeeper domain, hinge region, and DFG-motif. All these similar motifs are essential for their catalytic activity and design of novel inhibitors.

**Figure 2:**
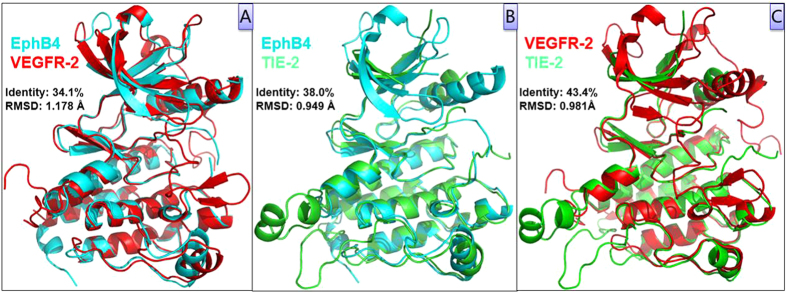
Protein structure alignment and superposition of three angiogenic RTKs. VEGFR-2 (Red), EphB4 (Cyan), and TIE-2 (Green).

Alignment of the crystal structures of three RTKs is depicted in Fig. 2. Structural alignment and comparison of independence and full sets of inter-residue distances indicated that there were no significant difference in root mean square distance (RMSD). The difference in RMSD for them are 1.178 Å, 0.949 Å, and 0.981 Å, respectively. As they displayed a similar two-lobed architecture, we believe that the large size of the RTK family and the similarity of kinase domains make the multiplex inhibition strategy a feasible drug discovery approach.

**Figure 3:**
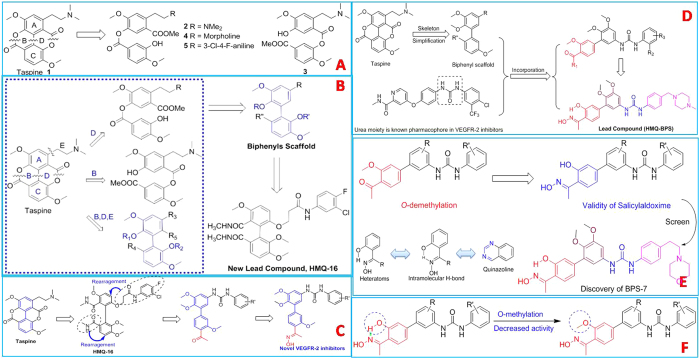
Structural optimization of taspine and identification of BPS-7.

Numbers of biphenyl-aryl ureas incorporated with salicylaldoxime have been developed as potent and selective VEGFR-2 inhibitors. We have identified salicylaldoxime as a novel HBG of multi-target RTK inhibitors. A novel biphenyl-aryl urea bearing salicylaldoxime (BPS-7) has been developed with natural alkaloid taspine as lead compound (Fig. 3). Fortunately, BPS-7 also displayed selective inhibition against angiogenic EphB4 and TIE-2. Moreover, it significantly inhibited the proliferation and migration of human umbilical vein endothelial cells^19^. Based on our previous findings, we propose that multiplex inhibition of VEGFR-2, TIE-2 and EphB4 would enhance anti-angiogenesis effect and reduce the occurrence of drug resistance^20^.

**Figure 4:**
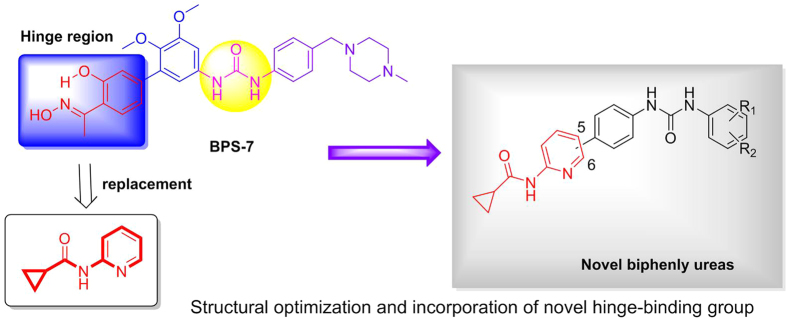
Exploration structural diversity of hinge-binding group (HBG) and structures of title compounds.

In order to continue our study in discovery of novel anti-angiogenesis agents, we explored the structural diversity of hinge-binding group (HBG) of BPS-7. *N*-pyridin-2-ylcyclopropane carboxamide was incorporated as HBG of VEGFR-2/TIE-2/EphB4 multiple inhibitors. We supposed that it might simultaneously form hydrogen bonds with hinge of three RTKs and therefore provide an opportunity to improve affinity. Moreover, the two methoxyl groups on biphenyl have been removed in order to reduce the steric hindrance of inhibitors when binding with receptors. In summary, we designed and synthesized a series of diaryl ureas incorporation of *N*-pyridin-2-ylcyclopropane carboxamide as novel multi-target anti-angiogenesis agents (Fig. 4). Biological evaluation and molecular modeling of these multiple VEGFR-2/TIE-2/EphB4 inhibitors were also carried out.

### Chemistry

**Figure 5:**
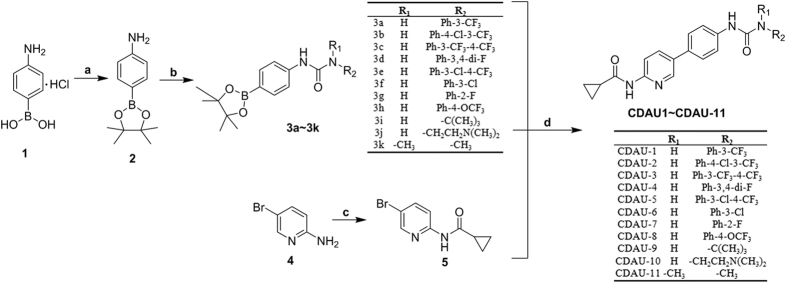
Synthetic route of the title compounds (CDAU-1~CDAU-11). *Reagents and conditions*: (**a**) Pinacol, MgSO_4_, Et_3_N, THF; (**b**) R-NH_2_, BTC, Et_3_N, DCM, 0 °C to rt; (**c**) Cyclopropanecarbonyl chloride, Et_3_N, DCM, 0 °C to rt; (**d**) Pd(PPh_3_)_4_, K_2_CO_3_, H_2_O, dioxane, reflux, 100 °C.

**Figure 6:**
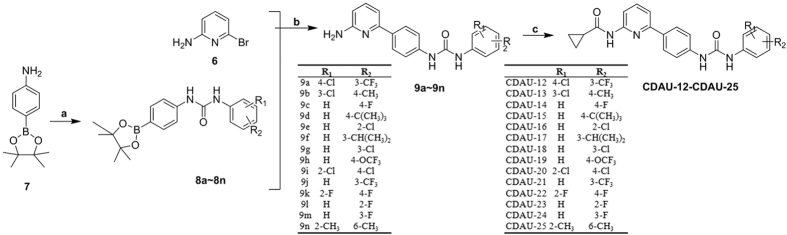
Synthetic route of the title compounds (CDAU-12~CDAU-25). *Reagents and conditions*: (**a**) R-NH_2_, BTC, Et_3_N, DCM, 0 °C to rt; (**b**) Pd(PPh_3_)_4_, K_2_CO_3_, H_2_O, CH_3_CN, reflux, 100 °C; (**c**) Cyclopropanecarbonyl chloride, Et_3_N, THF, 0 °C to rt.

The synthetic routes of title compounds were illustrated in Figs 5 and 6. Firstly, the key intermediates (3a-3k) were prepared in a two-step sequence from commercially available 4-aminophenylboronic acid (1). 4-Aaminophenylboronic acid (1) was converted to 4-aminophenylboronic acid pinacol ester (2)^11^. Then, various substituted anilines was treated with triphosgene to afford isocyanate, followed by reacting with intermediate (2) in dichloromethane to generate corresponding ureas (3a-3k). The key intermediate (5) was prepared from commercially available 2-amino-5-bromopyridine (4). Subsequently, the title commands (CDAU-1~CDAU-11) were prepared from (3a-3k) and (5) by Pd-catalyzed Suzuki coupling reaction^22^.

The synthetic steps of ureas (8a-8n) were similar to the synthesis of intermediates (3a-3k). The key intermediates (9a-9n) were prepared from corresponding ureas (8a-8n) and 2-amino-6-bromopyridine (6) by Pd-catalyzed Suzuki coupling reaction. Finally, cyclopropanecarbonyl chloride coupling of (9a-9n) through the acylation reaction afford the title compounds (CDAU-12~CDAU-25). All the title compounds characterized by ^1^H-NMR, ^13^C-NMR, high resolution mass spectrum (HRMS), and melting point analysis and their purity were above 95% determined by LC-MS (Supplementary Information).

### RTK inhibitory activity

All the title compounds were evaluated for their inhibitory potency against VEGFR-2, TIE-2 and EphB4 with sorafenib as positive control. Tyrosine kinase inhibition were tested by luminescent ADP-Glo^TM^ assay. As observed in Table 1, several compounds exhibited simultaneous inhibition against the three angiogenic RTKs. In particular, compound CDAU-1 and CDAU-2 displayed the most potent activity against VEGFR-2, TIE-2 and EphB4 with IC_50_ values of 1.11 nM, 7.20 nM, 5.34 nM (CDAU-1) and 1.01 nM, 8.32 nM, 5.11 nM (CDAU-2), respectively. In addition, another two compounds (CDAU-3 and CDAU-5) bearing trifluoromethyl group exhibited potent inhibitory activity against VEGFR-2 and TIE-2. The results indicated that dual-halogen substituent and trifluoromethyl were the most favorable for their enzymatic inhibitory activity. However, the compounds incorporated with aliphatic amine at terminal (CDAU-9, CDAU-10 and CDAU-11) showed the poorest activity. It might be due to the deficiency of interaction with allosteric site of RTKs.

For title compounds with cyclopropancarboxamid at *meta*-position of biphenyl (Table 2), the majority of them exhibited poor activity except for compound bearing 3-trifluoromethyl substituent on terminal aniline (CDAU-21). It displayed moderate RTKs (VEGFR-2, TIE-2 and EphB4) inhibitory activities with IC_50_ values of 6.27 nM, 42.76 nM, and 161.74 nM, respectively. These results indicated that the position of the cyclopropancarboxamid plays critical role in tyrosine kinase inhibition. *Para*-position was more favorable than *meta*-position for their potency. We speculate that *para*-position was beneficial for interaction with the hinge region. In addition, terminal anilines incorporated with trifluoromethyl were beneficial for VEGFR-2/TIE-2/EphB4 inhibitory activities. The inhibition against three angiogenic RTKs indicated that *N*-pyridin-2-ylcyclopropane carboxamide could serve as novel hinge-binding group for VEGFR-2/TIE-2/EphB4 multi-kinase inhibitors.

### Kinase Selectivity assays

We investigated the kinase selectivity of the most potent compounds (CDAU-1, CDAU-2) against other four kinases including B-Raf, FGFR-1, EGFR, and Src for its selective profile. The results were summarized in Table 3. The results revealed that they showed less potency against FGFR-1, EGFR, and Src compared with VEGFR-2, TIE-2, and EphB4. Moreover, they also exhibited potent B-Raf inhibitory activity. It was indicated that they exhibited moderate selectivity for VEGFR-2/TIE-2/EphB4 relative to other RTKs including FGFR-1, EGFR, and Src.

### Cell growth inhibition

On the basis of RTK inhibition assays, thirteen title compounds were selected and further evaluated for their anti-proliferative activity against human vascular endothelial cell (EA.hy926) with sorafenib as positive control. As shown in Table 4, the majority of them displayed moderate to high anti-proliferative activities with IC_50_ values ranging from 16.11 μM to 879.73 μM. Seven compounds (CDAU-1, -2, -7, -18, -19, -20, -21) exhibited potent inhibition against the growth of human vascular endothelial cell. Particularly, CDAU-1 and CDAU-2 exhibited the highest activity with IC_50_ values of 16.11 μM and 14.54 μM, respectively. These two compounds represent the first class of multiplex inhibitors with a “triplet” inhibition profile as well as anti-angiogenesis potency. They might not only inhibit the process of angiogenesis, but also prevent the occurrence of resistance.

### Molecular docking study

**Figure 7:**
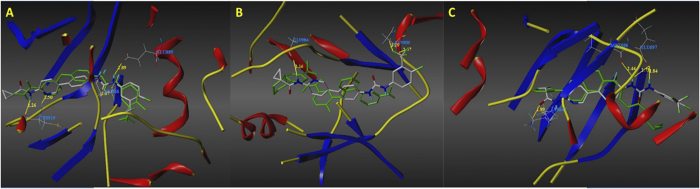
Low-energy docking model of the RTK/inhibitor complexes. Hydrogen bonds interactions are shown as dark-yellow dotted lines. (**A**)VEGFR-2 (PDB ID: 4ASD); (**B**) TIE-2 (PDB ID: 2P4I); (**C**) EphB4 (PDB ID: 4BB4).

For further structural optimization and investigation of the potential binding mode, molecular modeling studies were performed using Sybyl-X (Version 2.0, Tripos Inc. St. Louis, MO). The most potent compound, CDAU-2, was constructed and optimized using Powell’s method with a Tripos force field. The molecular modeling was performed using Sybyl-X/Surflex-dock module, and the residues in a 5.0 Å radius around the ligand of VEGFR-2 (PDB ID: 4ASD), TIE-2 (PDB ID: 2P4I) and EphB4 (PDB ID: 4BB4) were selected as the active site. The binding mode of CDAU-2 with the ATP-pocket of VEGFR-2 (A), TIE-2 (B), EphB4 (C) were depicted in Fig. 7. As shown in Fig. 7A, *N*-pyridin-2-ylcyclopropane carboxamide in CDAU-2 forms two hydrogen bonds with Cys 919 in the hinge region of VEGFR-2 with distance of 2.26 Å and 2.50 Å, respectively. In addition, the NH of urea formed one hydrogen bond with conserved Glu 885 for the bond length of 1.89 Å, while C = O formed a hydrogen bond with Asp 1046 of DFG-motif with distance of 1.67 Å. As for TIE-2, the binding model was described in Fig. 7B. NH of cyclopropylcarboxamide also formed a hydrogen bond with Cly 984 in hinge region of TIE-2 with the bond length of 2.16 Å. The fluorine atoms at terminal aniline form two hydrogen bonds with Asn 909 with distances of 2.19 Å and 2.67 Å, respectively. Favorable binding interactions of CDAU-2 with the active site of EphB4 included four hydrogen bonds (Fig. 7C): (1) the first forming between C = O of cyclopropylcarboxamide and NH of Lys 647 in hinge region, the distance was 1.89 Å; (2) another two hydrogen bonds forming between NH of urea and C = O of conserved Glu 697 with the distance of 2.73 Å and 1.84 Å, respectively; (3) the last one was observed between NH of urea and Met 696 with a distance of 2.44 Å. These molecular docking results indicated that the *N*-pyridin-2-ylcyclopropane carboxamide and urea groups are beneficial for affinity of inhibitors with VEGFR-2, TIE-2, and EphB4. Interestingly, *N*-pyridin-2-ylcyclopropane carboxamide could be considered as novel hinge-binding group for further discovery of multiple RTK inhibitors.

### Anti-angiogenesis potency on the tissue model for angiogenesis

**Figure 8:**
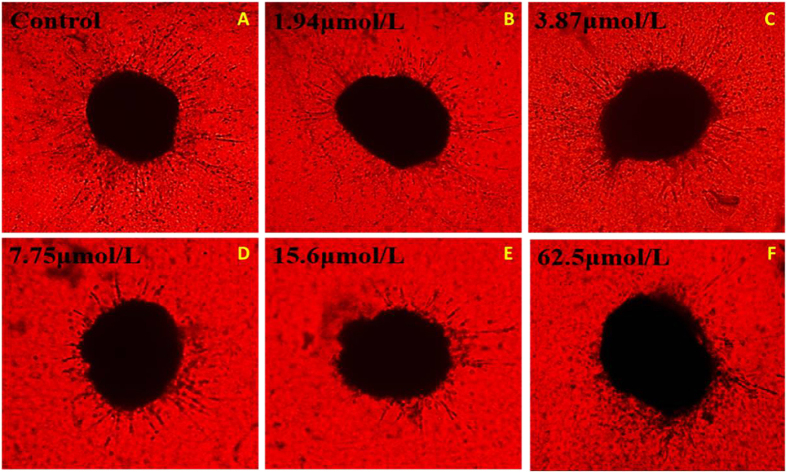
Anti-angiogenesis potency of compound CDAU-2 in tissue model for angiogenesis (TMA). (**A–F**) the representative images of lung tissue blood vessels in the TMA on the 5^th^ day; (**A**) the untreated control group; (**B–F**) lung tissue vessels in the CDAU-2 treated group; (**B**) 1.94 μM; (**C**) 3.87 μM; (**D**) 7.75 μM; (**E**) 15.6 μM; (F) 62.5 μM. Vessels grew normally in control group; vessels in the CDAU-2 treated group exhibited the slow increase compared with the control group.

A novel tissue model for angiogenesis that imitated new blood vessels formation *in vivo* had been established in the previous study^23^. Here, it was used to evaluate the anti-angiogenesis potency of the most potent CDAU-2. The results indicated that CDAU-2 could effectively inhibit the blood vessels formation (Fig. 8). CDAU-2 inhibited vessels outgrew from the periphery of the lung tissues evidently at 15.6 μM, and exhibited good reproducibility. Furthermore, CDAU-2 could potently inhibit vessels growth at the tested concentrations.

## Discussion

**Figure 9:**
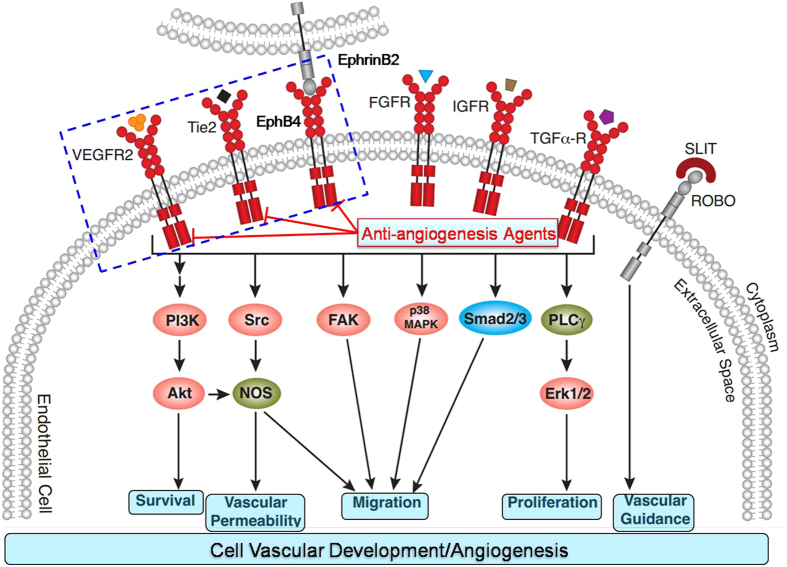
Design strategy and potential action mechanism of multi-target anti-angiogenesis agents with VEGFR-2/TIE-2/EphB4 as targets. Simultaneous blockade of VEGFR-2/TIE-2/EphB4 signaling pathways leads to inhibition of EC survival, vascular permeability, migration, and proliferation within angiogenesis.

Herein, we disclosed the first class of multi-target inhibitors with a “triplet” inhibition profile. Extensive investigations linked drug resistance with compensatory activation of angiogenic RTKs, especially for VEGFR-2, TIE-2, and EphB4. Moreover, complexity and heterogeneity of angiogenesis make it difficult to be treated with single target agents. Accordingly, we proposed that multiple inhibition of RTKs could enhance the efficacy and overcome the resistance on the basis of vascular normalization concept. Meanwhile, it is feasible to develop multiple inhibitors against VEGFR-2/TIE-2/EphB4 because of their highly conserved DFG-out conformation. These novel strategies have yielded promising results in the discovery of anti-angiogenesis agents. We have developed the first class of multiple inhibitors of VEGFR-2/TIE-2/EphB4. Simultaneous blockade of VEGFR-2/TIE-2/EphB4 signaling pathways leads to inhibition of endothelial cell survival, vascular permeability, migration, and proliferation within angiogenesis (Fig. 9). These novel inhibitors might contribute to the discovery of novel anti-angiogenesis agents with VEGFR-2/TIE-2/EphB4 as multiple targets.

## Conclusion

In conclusion, we described the discovery of multi-target inhibitors as novel anti-angiogenesis agents. *N*-pyridin-2-ylcyclopropane carboxamide was firstly introduced to diaryl urea core as hinge-binding group. A series of diarylureas incorporated of *N*-pyridin-2-ylcyclopropane carboxamide were designed, synthesized and evaluated as multi-target inhibitors of VEGFR-2/TIE-2/EphB4. The biological evaluation revealed that compounds CDAU-1 and CDAU-2 exhibited the most potent inhibitory activity against VEGFR-2, TIE-2 and EphB4. Meanwhile, they displayed potent anti-proliferative activity against human vascular endothelial cell (EA. hy926). These two compounds represented the first class of multiple VEGFR-2/EphB4/TIE-2 inhibitors with a “triplet” inhibition profile. The most potent CDAU-2 also exhibited promising anti-angiogenic potency in a tissue model for angiogenesis. Moreover, biological evaluation and molecular docking indicated that *N*-pyridin-2-ylcyclopropane carboxamide is beneficial for potency of these multi-target inhibitors. It could be considered as a novel hinge-binding group for further discovery of multi-target anti-angiogenesis agents. Our results may contribute to the discovery of novel anti-angiogenesis agents for the intervention of pathological angiogenesis-related diseases.

## Methods

### A statement identifying the institutional and/or licensing committee experimental approval

All experimental protocols were carried out in accordance with relevant guidelines and regulations and approved by the Ethical Committee of Xi’an Jiaotong University.

### Chemical Synthesis

Solvents and reagents are purified according to the standard procedure. Reactions are monitored by thin layer chromatography on 0.25-mm silica gel plates (60GF-254) and visualized with UV light. The reactions except those in aqueous media are carried out by standard techniques for the exclusion of moisture. Melting points are determined on electrothermal melting point apparatus and are uncorrected. ^1^H NMR and ^13^C NMR spectra are measured at 400 MHz on a Bruker Advance AC 400 instrument with TMS as an internal standard. High resolution mass spectra (HRMS) are obtained on a Shimadzu LCMS-IF-TOF instrument.

#### 4-(4,4,5,5-tetramethyl-1,3,2-dioxaborolan-2-yl) aniline (2)

A round bottom flask charged with 4-aminobenzeneboronic acid hydrochloride (1) (4.00 g, 23.07 mmol), pinacol (4.09 g, 34.61 mmol) and MgSO_4_ (8.22 g, 68.52 mmol), Et_3_N (20 mL), THF (100 mL) was then added, the mixture was stirred at room temperature for 5 h. After completion of reaction, the reaction mixture the distilled by rotary evaporation to remove THF afforded (2) (4.01 g, 79.41%) as white solid.

#### 1-(4-(4,4,5,5-tetramethyl-1,3,2-dioxaborolan-2-yl)phenyl)-3-(3-(trifluoromethyl)phenyl)urea (3a)

Triphosgene (0.80 g, 2.74 mmol) was dissolved in anhydrous CH_2_Cl_2_ (20 mL) and the mixture was stirred on ice-bath for 5 min. A solution of 3-(trifluoromethyl)aniline (1.10 g, 6.85 mmol) in anhydrous CH_2_Cl_2_ was added dropwise to the above mixture and stirring was continued for 15 min. Then triethanolamine (1.15 mL, 8.22 mmol) diluted with CH_2_Cl_2_ (10 mL) was then added onto the mixture. Stirring was continued for 15 min, a solution of triethanolamine (1.15 mL, 8.22 mmol) and intermediate (2) (1.50 g, 6.85 mmol) in anhydrous CH_2_Cl_2_ (10 mL) was added and continued stirring for 20 min. Subsequently, the ice bath was removed, and the mixture was reacted at room temperature overnight. After completion of the action, the reaction was quenched with dilute NaHCO_3_. The organic layer was washed with water and brine, and dried over Na_2_SO_4_. After filtration and concentration *in vacuo*, the residues was purified by silica gel flash chromatography (PE/AcOEt = 7:1) affording (3a) as white solid (1.77 g, 63.67%).

#### N-(5-bromopyridin-2-yl)cyclopropanecarboxamide (5)

2-Amino-5-bromopyridine (4) (1.00 g, 5.78 mmol) was dissolved in anhydrous CH_2_Cl_2_ (20 mL) and the mixture was stirred on the ice-bath. Triethanolamine (2.89 mL, 20.75 mmol) diluted with CH_2_Cl_2_ (10 mL) was then added onto the mixture. Stirring was continued for 30 min, a solution of cyclopropanecarbonyl chloride (1.05 mL, 11.65 mmol) in anhydrous CH_2_Cl_2_ was added dropwise to the above mixture. Then, the ice bath was removed, and the mixture was reacted at room temperature overnight. The product was extracted with CH_2_Cl_2_ (20 mL × 3), washed twice with water and brine, and dried over Na_2_SO_4_. After filtration and concentration *in vacuo*, the residues was purified by silica gel flash chromatography (PE/AcOEt = 5:1) afford (5) as white solid (1.28 g, 97.71%).

#### N-(5-(4-(3-(3-(trifluoromethyl)phenyl)ureido)phenyl)pyridin-2-yl)cyclopropanecarboxamide (CDAU-1)

A flask charged with Pd(PPh_3_)_4_ (0.45 g, 0.39 mmol), potassium carbonate (1.63 g, 11.82 mmol), and intermediate (3a) (1.60 g, 3.94 mmol) and (5) (0.89 g, 3.94 mmol) were flushed with nitrogen and suspended in 1,4-dioxane (90 mL) and water (30 mL). The mixture was then refluxed overnight under nitrogen. The hot suspension was filtered and the filtrate distilled by rotary evaporation to remove 1,4-dioxane. Water (50 mL) was added and the product was extracted with AcOEt (30 mL × 3), washed with water, and dried over Na_2_SO_4_. After filtration and concentration *in vacuo*, the residue was purified by silica gel flash chromatography (PE/AcOEt = 3:1) affording CDAU-1 (0.23 g, 13.22%) as white solid. HRMS *m/z* calcd for C_23_H_19_F_3_N_4_O_2_ ([M + H]^+^) 441.1538, found 441.1514, mp: 270~272 °C. ^1^H NMR (400 MHz, DMSO*-d*_*6*_) δ 10.87 (s, 1H), 9.21 (s, 1H), 8.99 (s, 1H), 8.63 (d, *J* = 2.2 Hz, 1H), 8.14 (d, *J* = 8.7 Hz, 2H), 8.04 (m, *J* = 8.7, 2.4 Hz, 1H), 7.65 (t, *J* = 8.3 Hz, 4H), 7.58 (d, *J* = 8.7 Hz, 2H), 2.03 (t, *J* = 12.3, 8.6, 4.8 Hz, 1H), 0.92–0.71 (m, 4H). ^13^C NMR (101 MHz, DMSO*-d*_*6*_) δ 173.01, 152.91, 151.41, 145.65, 141.00, 139.51, 135.96, 131.04, 130.98, 130.40, 130.15, 129.84, 127.15, 126.04, 123.34, 122.36, 119.35, 118.58, 114.65, 114.61, 113.69, 14.69, 8.15.

All the title compounds **CDAU-2~CDAU-11** were prepared using the general procedure described above.Their structures were characterized by ^1^H-NMR, ^13^C-NMR, high resolution mass spectrum (HRMS), and melting point analysis and their purity were above 95% determined by LC-MS (Supplementary Information).

#### 1-(4-chloro-3-(trifluoromethyl)phenyl)-3-(4-(4,4,5,5-tetramethyl-1,3,2-dioxaborolan-2-yl)phenyl)urea (8a)

Triphosgene (1.40 g, 4.77 mmol) was dissolved in anhydrous CH_2_Cl_2_ (30 mL) and the mixture was stirred on the ice-bath for 5 min. A solution of 4-chloro-3-(trifluoromethyl)aniline (1.50 g, 10.59 mmol) in anhydrous CH_2_Cl_2_ was added dropwise to the above mixture and stirring was continued for 15 min. Then triethanolamine (1.75 mL, 12.71 mmol) diluted with CH_2_Cl_2_ (20 mL) was added onto the mixture. Stirring was continued for 15 min, a solution of triethanolamine (1.75 mL, 12.71 mmol) and 4-(4,4,5,5-tetramethyl-1,3,2-dioxaborolan-2-yl)aniline (7) (1.86 g, 8.47 mmol) in anhydrous CH_2_Cl_2_ (30 mL) was added and continued stirring for 20 min. Subsequently, the ice bath was removed, and the mixture was reacted at room temperature overnight. After completion of the action, the reaction was quenched with dilute NaHCO_3_.The organic layer was washed with water and brine, and dried over Na_2_SO_4_. After filtration and concentration *in vacuo*, the residues was purified by silica gel flash chromatography (PE/AcOEt = 3:1) affording (8a) as white solid (3.03 g, 65.00%).

#### 1-(4-(6-aminopyridin-2-yl)phenyl)-3-(4-chloro-3-(trifluoromethyl)phenyl)urea (9a)

A flask charged with Pd(PPh_3_)_4_ (0.31 g, 0.27 mmol), cesium carbonate (1.77 g, 5.44 mmol), intermediate (8a) (1.20 g, 2.72 mmol) and (5) (0.89 g, 3.94 mmol) were flushed with nitrogen and suspended in acetonitrile (90 mL) and water (60 mL). The mixture was then refluxed overnight under nitrogen atmosphere. The hot suspension was filtered and the filtrate distilled by rotary evaporation to remove 1,4-dioxane. Water (50 mL) was added and the product was extracted with AcOEt (30 mL × 3), washed with water, and dried over Na_2_SO_4_. After filtration and concentration *in vacuo*, the residue was purified by silica gel flash chromatography (PE/AcOEt = 3:1) affording (9a) (0.50 g, 45.50%) as yellow solid.

#### N-(6-(4-(3-(4-chloro-3-(trifluoromethyl)phenyl)ureido)phenyl)pyridin-2-yl)cyclopropanecarboxamide (CDAU-12)

To a mixture of (9a) (0.20 g, 0.57 mmol) and triethanolamine (0.32 mL, 2.28 mmol) dissolved in 20 mL of THF in ice-bath. After stirring for 30 min, cyclopropanecarbonyl chloride (0.12 mL, 1.36 mmol) was added dropwise. The reaction was warmed to the room temperature and stirred overnight. After the completion of the reaction, the mixture was filtered and the filtrate distilled by rotary evaporation to remove THF. After filtration and concentration *in vacuo*, the residues was purified by silica gel flash chromatography (PE/AcOEt = 1:1) yielding (CDAU-12) as white solid (0.09 g, 33.33%). HRMS *m/z* calcd for C_23_H_18_ClF_3_N_4_O_2_ ([M +H]^+^) 474.1070, found 475.0081, mp:207~209 °C, ^1^H NMR (400 MHz, DMSO*-d*_*6*_) δ 10.74 (s, 1H), 9.23 (s, 1H), 9.05 (s, 1H), 8.15 (d, *J* = 2.2 Hz, 1H), 8.04 (d, *J* = 8.8 Hz, 2H), 7.98 (d, *J* = 8.2 Hz, 1H), 7.80 (t, *J* = 7.9 Hz, 1H), 7.65 (d, *J* = 3.2 Hz, 2H), 7.61 (d, *J* = 5.4 Hz, 2H), 7.59 (d, *J* = 4.1 Hz, 1H), 2.09 (m, *J* = 7.4, 5.1 Hz, 1H), 0.83 (m, *J* = 9.0, 3.2 Hz, 4H). ^13^C NMR (101 MHz, DMSO*-d*_*6*_) δ 176.02, 173.17, 154.82, 152.76, 152.19, 140.69, 139.73, 139.49, 132.62, 132.49, 132.00, 129.17, 127.63, 123.63, 118.81, 117.27, 115.19, 112.04, 25.42, 14.68, 8.10.

All the title compounds CDAU-13~CDAU-25 were prepared using the general procedure described above. Their structures were characterized by ^1^H-NMR, ^13^C-NMR, high resolution mass spectrum (HRMS), and melting point analysis and their purity were above 95% determined by LC-MS (Supplementary Information).

### Angiogenic RTK inhibition evaluation

The *in vitro* kinase inhibition assays against VEGFR-2, TIE-2, and EphB4 of all the title compounds were detected using the ADP-Glo™ kinase assay kit (Promega, Madison) with sorafenib as positive control^24^. The kinase assay was performed in duplicate in a reaction mixture of final volume of 10 μL. General procedures are as the following: for VEGFR-2 assays, the tyrosine kinase (0.6 ng/mL) were incubated with substrates (0.2 mg/mL), tested title compounds (1.2 × 10^−4^~12 μM) and ATP (50 μM) in a final buffer of Tris 40 mM, MgCl_2_ 10 mM, BSA 0.1 mg/mL, DTT 1 mM in 384-well plate with the total volume of 5 μL. The assay plate was incubated at 30 °C for 1 h. After the plate was cooled at room temperature for 5 min, 5 μL of ADP-Glo reagent was added into each well to stop the reaction and consume the remaining ADP within 40 min. At the end, 10 μL of kinase detection reagent was added into the well and incubated for 30 min to produce a luminescence signal. As for TIE-2 and EphB4 assays, the tyrosine kinase (2.4 ng/mL) were incubated with substrates (0.2 mg/mL), tested title compounds (1.2 × 10^−4^~12 μM) and ATP (50 μM) in a final buffer of Tris 40 mM, MgCl_2_ 10 mM, BSA 0.1 mg/mL, DTT 1 mM in 384-well plate with the total volume of 5 μL. The assay plate was incubated at 30 °C for 4 h. After the plate was cooled at room temperature for 5 min, 5 μL of ADP-Glo reagent was added into each well to stop the reaction and consume the remaining ADP within 1 h. At the end, 10 μL of kinase detection reagent was added into the well and incubated for 30 min to produce a luminescence signal. The luminescence was read by VICTOR-X multi- label plate reader. The signal was correlated with the amount of ATP present in the reaction and was inversely correlated with the kinase activity.

### Cell growth inhibitory activity in cancer cell lines

Growth inhibitory activities were evaluated against human vascular endothelial cell (EA.hy926)^25^. Thirteen selected title compounds were tested using MTT assay to assess cell proliferation. Exponentially growing cells were harvested and plated in 96-well plates at a concentration of 1 × 1^4 ^cells/well, and then incubated for 24 h at 37 °C. The cells were treated with title compounds respectively at various concentrations for 48 h. Then, 22 mL fresh MTT (5 mg/mL) was added to each well and incubated for 4 h at 37 °C. Supernatant was discarded, and 150 mL DMSO was added to each well. Absorbance values were determined by a microplate reader (Bio-Rad Instruments) at 490 nm. The IC_50_ values were calculated according to inhibition ratios.

### Molecular docking modeling

In order to understand the binding mode of inhibitors with VEGFR-2/TIE-2/EphB4, molecule docking was performed using Sybyl-X/Surflex-dock module based on the crystal structures of VEGFR-2 (PDB ID: 4ASD), TIE-2 (PDB ID: 2P4I) and EphB4 (PDB ID: 4BB4)^26^. Hydrogen was added and minimized using the Tripos force field and Pullman charges. The most potent compound (CDAU-A2) was depicted with the Sybyl-X/Sketch module (Tripos Inc.) and optimized applying Powell’s method with the Tripos force field with convergence criterion set at 0.05 kcal/(Åmol), and assigned with the Gasteiger-Hückel charge. The docking studied was carried out using Surflex-dock module. The residues in a radius 5.0 Å around the ligand of VEGFR-2/TIE-2/ EphB4 in the crystal complex were selected as the active site. Other docking parameters were kept at default.

### Screening of CDAU-2 on the tissue model for angiogenesis

In brief, the mouse lung tissue was separated and cut into pieces (0.5–1.0 mm^3^)^27^. Then pieces of lung tissue were placed onto 48-well plate coated with polymerized fibrinogen with thrombin. After consolidation, each group was incubated with 200 μL/well 1640 medium containing different concentrations of CDAU-2. Control groups were incubated with 2.5% DMSO or 1640 medium alone. The sprouting vessels were observed at the fifth day post treatment.

## Additional Information

**How to cite this article:** Wang, J. *et al*. Discovery of multi-target receptor tyrosine kinase inhibitors as novel anti-angiogenesis agents. *Sci. Rep.* **7**, 45145; doi: 10.1038/srep45145 (2017).

**Publisher's note:** Springer Nature remains neutral with regard to jurisdictional claims in published maps and institutional affiliations.

## Supplementary Material

Supplementary Information

## Figures and Tables

**Table 1:** Structures and RTK inhibitory activities of title compounds (CDAU-1~CDAU-11).

Compound	R_1_	R_2_	VEGFR-2(nM)	Tie-2(nM)	EphB4(nM)
CDAU-1	H	Ph-3-CF_3_	1.11	7.20	5.34
CDAU-2	H	Ph-4-Cl-3-CF_3_	1.01	8.32	5.11
CDAU-3	H	Ph-3-CF_3_-4-CF_3_	2.00	27.53	>1000
CDAU-4	H	Ph-3,4-di-F	1.42	>1000	ND
CDAU-5	H	Ph-3-Cl-4-CF_3_	5.52	61.26	>1000
CDAU-6	H	Ph-3-Cl	23.31	32.91	948.01
CDAU-7	H	Ph-2-F	>1000	>1000	>1000
CDAU-8	H	Ph-4-OCF_3_	>1000	>1000	>1000
CDAU-9	H	-C(CH_3_)_3_	>1000	857.65	>1000
CDAU-10	H	-CH_2_CH_2_N(CH_3_)_2_	>1000	>1000	>1000
CDAU-11	-CH_3_	-CH_3_	>1000	>1000	>1000
**Sorafenib**			0.77	2.77	1.05

ND = not determined.

**Table 2:** Structures and RTK inhibitory activities oftitle compounds (CDAU-12~CDAU-25).

Compound	R_1_	R_2_	VEGFR-2 (nM)	TIE-2 (nM)	EphB4 (nM)
CDAU-12	4-Cl	3-CF_3_	>1000	>1000	>1000
CDAU-13	3-Cl	4-CH_3_	>1000	271.49	>1000
CDAU-14	H	4-F	ND	32.92	>1000
CDAU-15	H	4-C(CH_3_)_3_	28.05	>1000	ND
CDAU-16	H	2-Cl	73.39	>1000	>1000
CDAU-17	H	3-CH(CH_3_)_2_	62.97	ND	169.62
CDAU-18	H	3-Cl	ND	4.92	>1000
CDAU-19	H	4-OCF_3_	33.97	ND	>1000
CDAU-20	2-Cl	4-Cl	42.72	78.76	ND
CDAU-21	H	3-CF_3_	6.27	42.76	161.74
CDAU-22	2-F	4-F	>1000	>1000	ND
CDAU-23	H	2-F	>1000	>1000	ND
CDAU-24	H	3-F	>1000	>1000	ND
CDAU-25	2-CH_3_	6-CH_3_	ND	>1000	>1000
**Sorafenib**			0.55	4.65	3.00

ND = not determined.

**Table 3:** Receptor tyrosine kinase selectivity profile of the most active compounds (IC_50_, nM).

Compound	VEGFR-2	Tie-2	EphB4	B-Raf	FGFR-1	EGFR	Src
CDAU-1	1.11	7.20	5.34	57.24	155.31	204.21	>500
CDAU-2	1.01	8.32	5.11	34.62	317.43	190.89	>500

**Table 4:** Anti-proliferative activities of inhibitors against human vascular endothelial cell EA.hy 926 cell (IC_50_, μM).

Compound	EA.hy926	Compound	EA.hy926
CDAU-1	16.11	CDAU-10	>1000
CDAU-2	14.54	CDAU-17	133.18
CDAU-3	309.43	CDAU-18	22.60
CDAU-4	84.95	CDAU-19	31.01
CDAU-5	151.91	CDAU-20	45.68
CDAU-6	879.73	CDAU-21	40.51
CDAU-7	46.22	Sorafenib	12.49
