# Supplementary material for: Discovery of multi-target receptor tyrosine kinase inhibitors as novel anti-angiogenesis agents
Source: Sci Rep. 2017 Mar 23;7:45145. doi: 10.1038/srep45145 (PMC5362808; doi:10.1038/srep45145)
Supplement: Supplementary Information [file srep45145-s1.pdf]

Discovery of multi-target receptor tyrosine kinase inhibitors as novel anti-angiogenesis agents

Jinfeng Wang, Lin Zhang, Xiaoyan Pan, Bingling Dai, Ying Sun, Chuansheng Li, Jie Zhang*

School of Pharmacy, Health Science Center, Xi'an Jiaotong University, No. 76, Yanta West Road, Xi'an, 710061, P.R. China

Table of Contents

1. **The nomenclature, structure, melting point, HRMS, ^{1}H-NMR spectra, ^{13}C-NMR spectra of Title Compounds (CDAU-1~CDAU-11).**
2. **The nomenclature, structure, melting point, HRMS, ^{1}H-NMR spectra, ^{13}C-NMR spectra of Title Compounds (CDAU-12~CDAU-25).**

* Corresponding author. Tel/Fax: +86-29-82655451; E-mail: zhj8623@xjtu.edu.cn (Jie Zhang).

N-(5-(4-(3-(3-(trifluoromethyl)phenyl)ureido)phenyl)pyridin-2-yl)cyclopropanecarboxamide(CDAU-1)

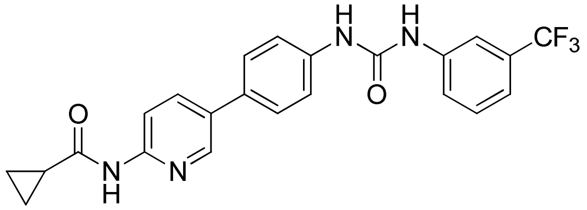

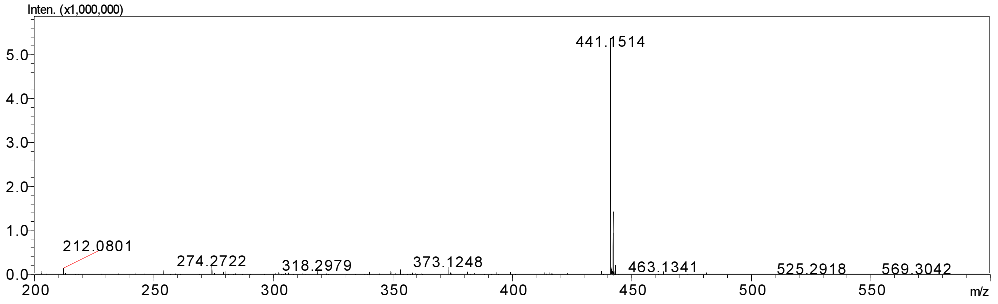

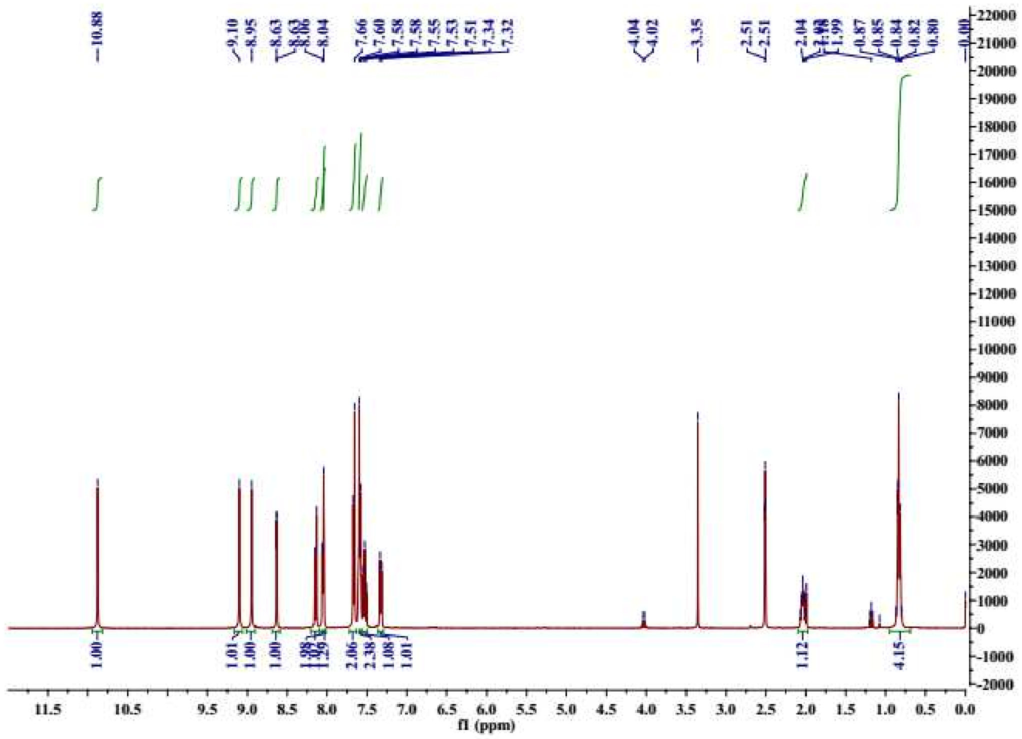

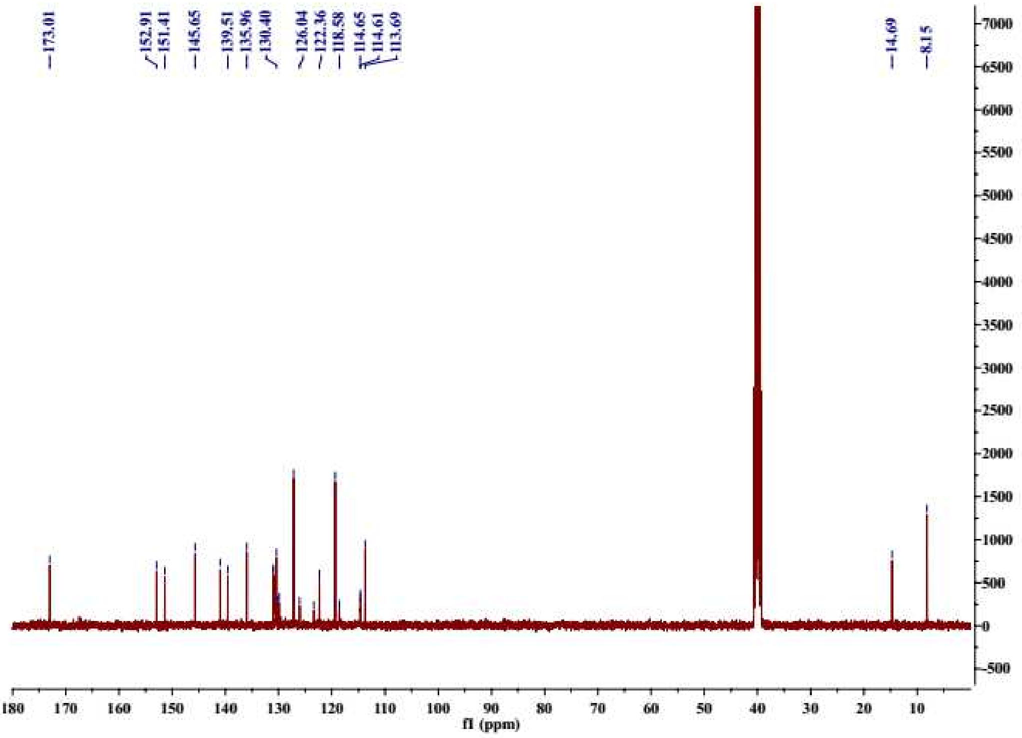

mp:270~272℃, HRMS *m/z* calcd for $C_{23}H_{19}F_3N_4O_2$ ($[M+H]^+$) 441.1538, found 441.1514. ^1H NMR (400 MHz, DMSO-d_6) δ 10.87 (s, 1H), 9.21 (s, 1H), 8.99 (s, 1H), 8.63 (d, *J* = 2.2 Hz, 1H), 8.14 (d, *J* = 8.7 Hz, 2H), 8.04 (m, *J* = 8.7, 2.4 Hz, 1H), 7.65 (t, *J* = 8.3 Hz, 4H), 7.58 (d, *J* = 8.7 Hz, 2H), 2.03 (t, *J* = 12.3, 8.6, 4.8 Hz, 1H), 0.92 – 0.71 (m, 4H).^{13}C NMR (101 MHz, DMSO-d_6) δ 173.01, 152.91, 151.41, 145.65, 141.00, 139.51, 135.96, 131.04, 130.98, 130.40, 130.15, 129.84, 127.15, 126.04, 123.34, 122.36, 119.35, 118.58, 114.65, 114.61, 113.69, 14.69, 8.15.

N-(5-(4-(3-(4-chloro-3-(trifluoromethyl)phenyl)ureido)phenyl)pyridin-2-yl)cyclopropanecarboxamide(CDAU-2)

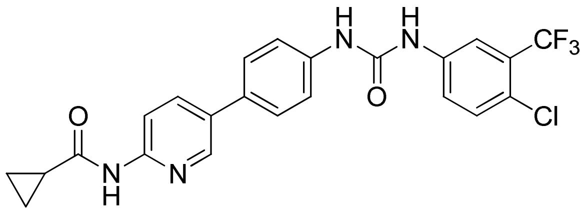

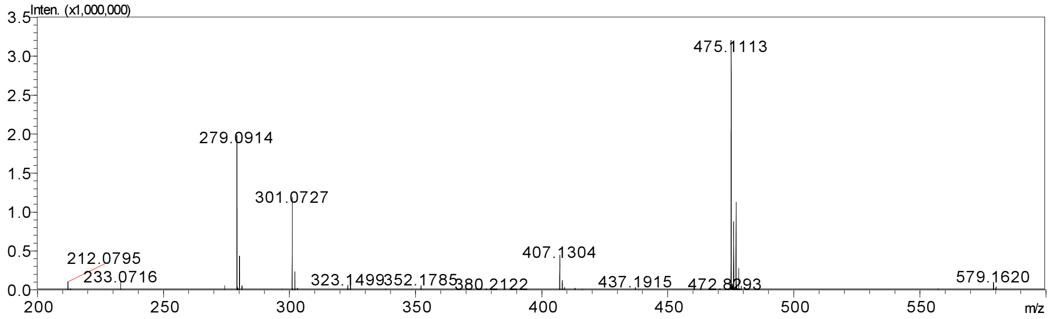

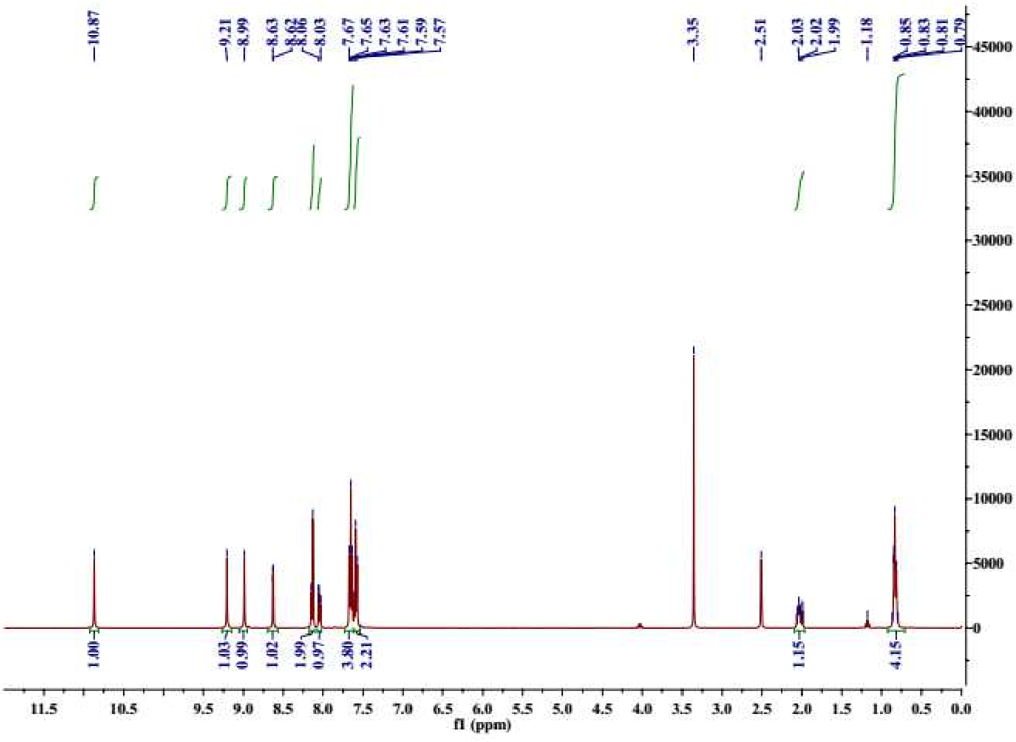

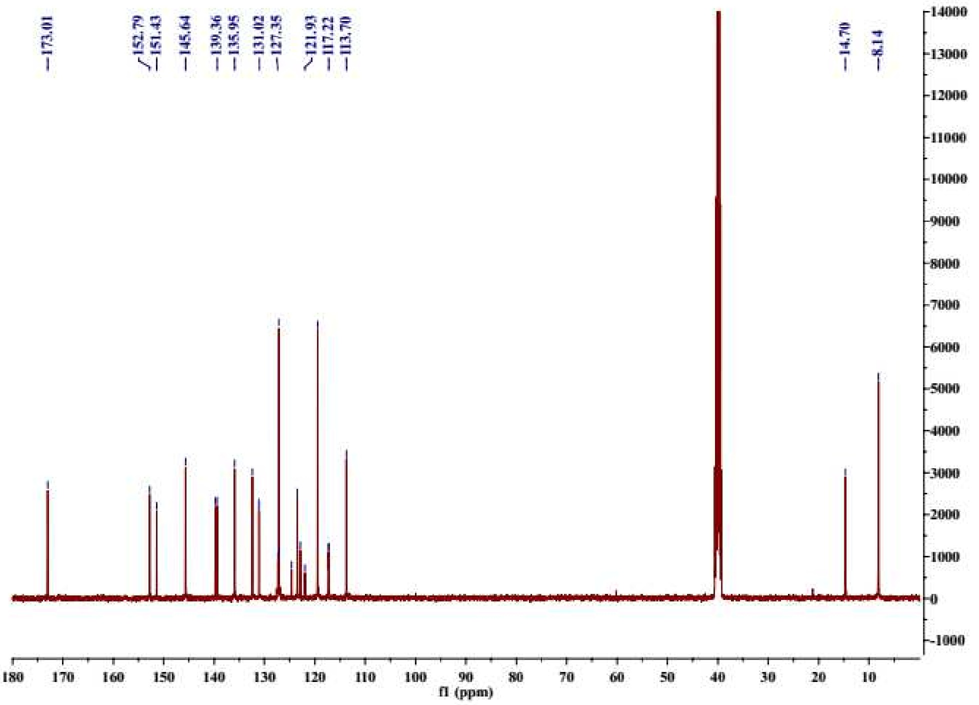

mp:273~275℃, HRMS *m/z* calcd for $C_{23}H_{18}ClF_3N_4O_2$ ($[M+H]^+$) 475.1149, found 475.1113. ^1H NMR (400 MHz, DMSO-d_6) δ 10.88 (s, 1H), 9.10 (s, 1H), 8.95 (s, 1H), 8.63 (d, *J* = 2.4 Hz, 1H), 8.14 (d, *J* = 8.7 Hz, 1H), 8.09 – 8.02 (m, 1H), 8.04 (s, 1H), 7.67 (d, *J* = 8.7 Hz, 2H), 7.60 – 7.57 (m, 2H), 7.53 (t, *J* = 7.9 Hz, 1H), 7.33 (d, *J* = 7.6 Hz, 1H), 2.03 (m, *J* = 12.1, 8.5, 4.7 Hz, 1H), 0.95 – 0.69 (m, 4H).^{13}C NMR (101 MHz, DMSO-d_6) δ 173.01, 152.79, 151.43, 145.64, 139.77, 139.36, 135.95, 132.42, 131.13, 131.02, 127.35, 127.13, 127.04, 124.64, 123.51, 122.82, 121.93, 119.47, 117.28, 117.22, 113.70, 14.70, 8.14.

N-(5-(4-(3-(3,4-bis(trifluoromethyl)phenyl)ureido)phenyl)pyridin-2-yl)cyclopropanecarboxamide(CDAU-3)

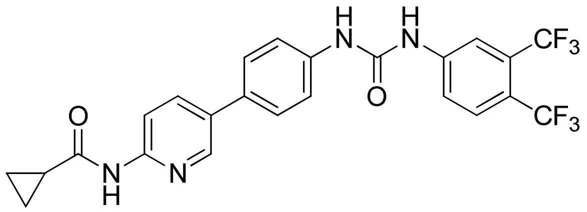

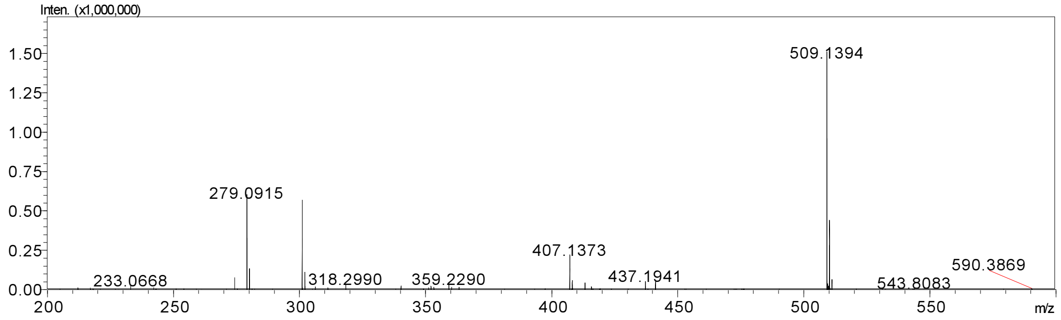

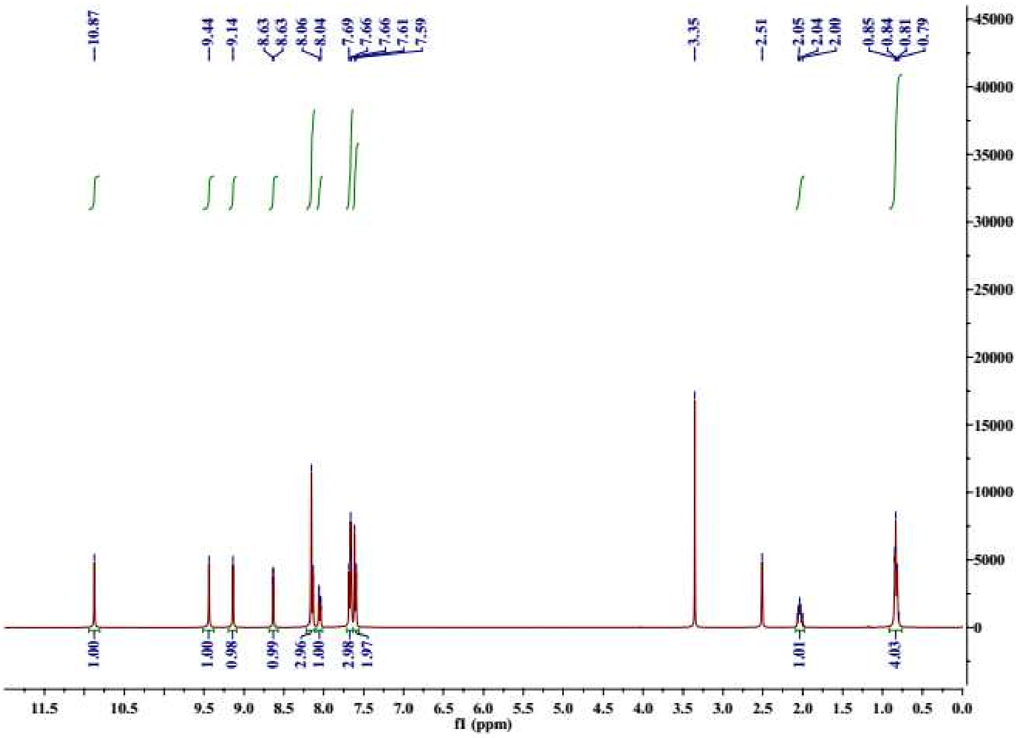

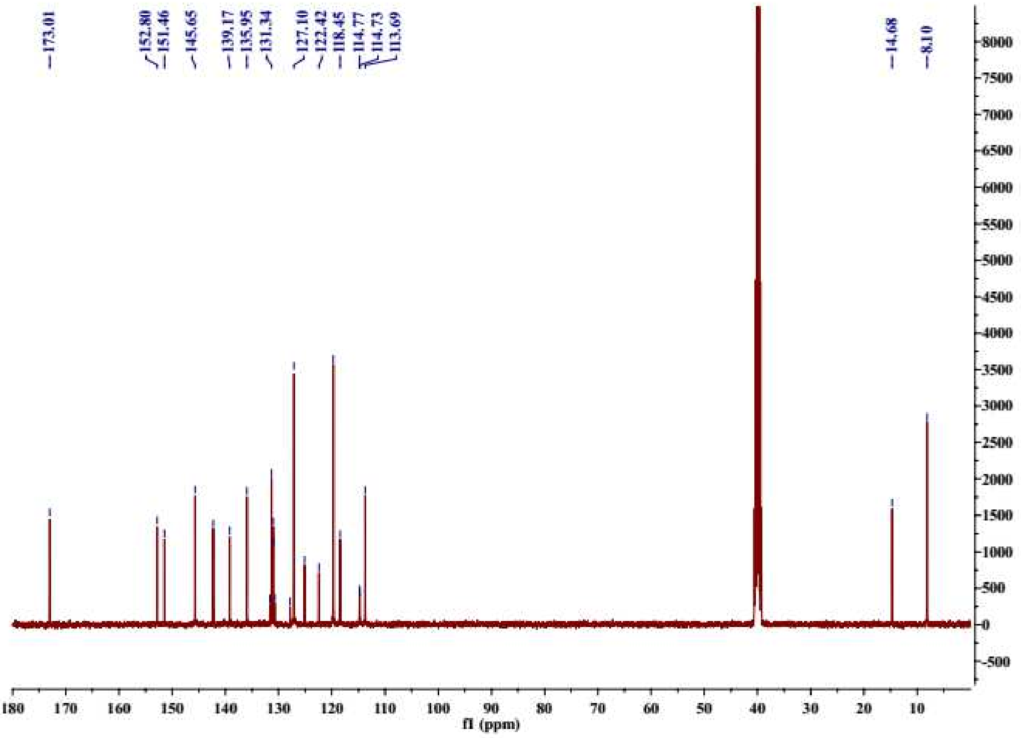

mp:241~243℃, HRMS *m/z* calcd for $C_{24}H_{18}F_6N_4O_2$ ($[M+H]^+$) 508.1412, found 509.1394. ^{1}H NMR (400 MHz, DMSO-d_6) δ 10.87 (s, 1H), 9.44 (s, 1H), 9.14 (s, 1H), 8.63 (d, *J* = 2.1 Hz, 1H), 8.15 (d, *J* = 8.9 Hz, 3H), 8.05 (m, *J* = 8.7, 2.4 Hz, 1H), 7.67 (t, *J* = 5.9 Hz, 3H), 7.60 (d, *J* = 8.7 Hz, 2H), 2.09 – 1.98 (m, 1H), 0.82 (m, *J* = 14.6, 6.4 Hz, 4H).^{13}C NMR (101 MHz, DMSO-d_6) δ 173.01, 152.80, 151.46, 145.65, 142.28, 139.17, 135.95, 131.66, 131.34, 131.01, 130.98, 130.69, 127.84, 127.10, 125.13, 122.42, 119.69, 118.45, 114.77, 114.73, 113.69, 14.68, 8.10.

N-(5-(4-(3-(3,4-difluorophenyl)ureido)phenyl)pyridin-2-yl)cyclopropanecarboxamide(CDAU-4)

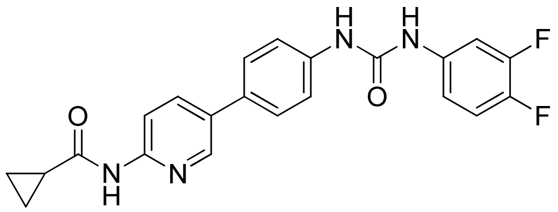

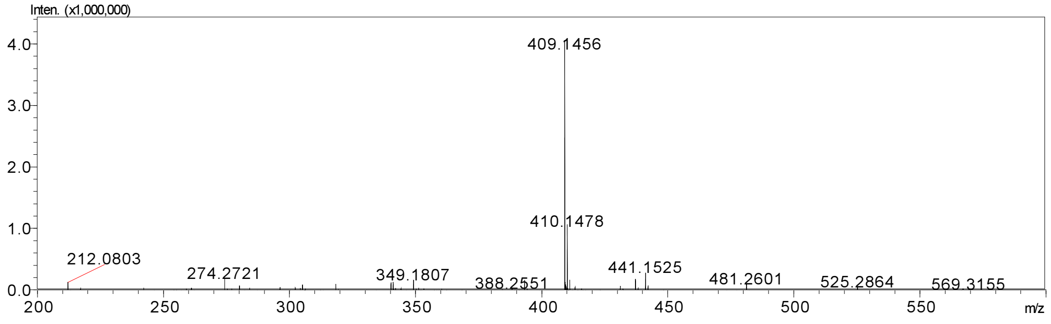

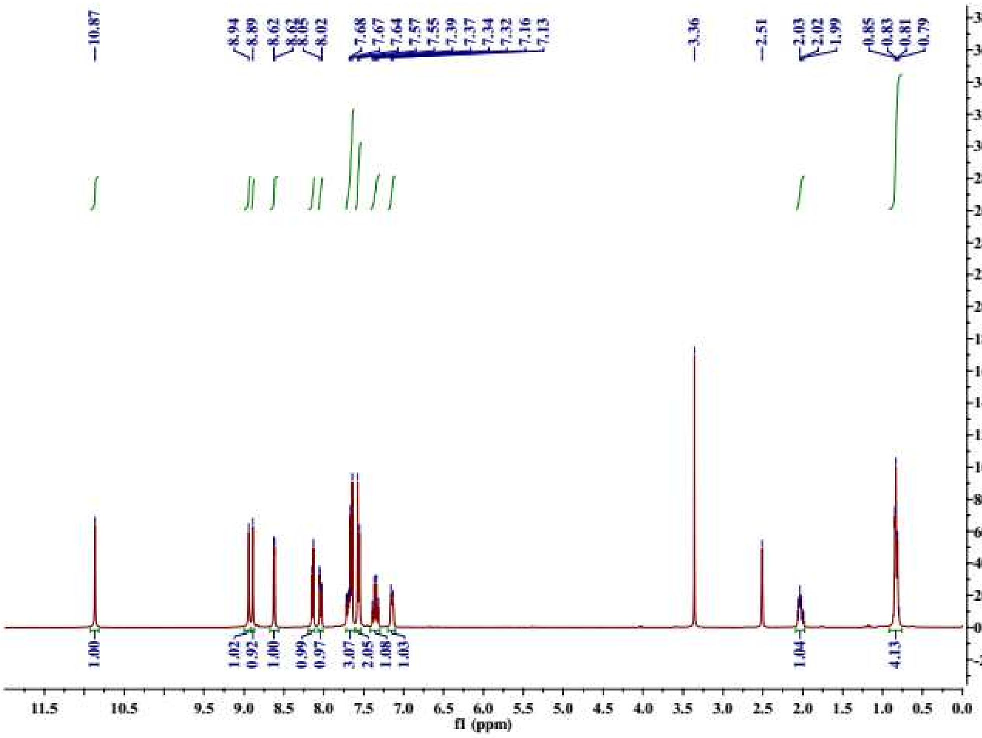

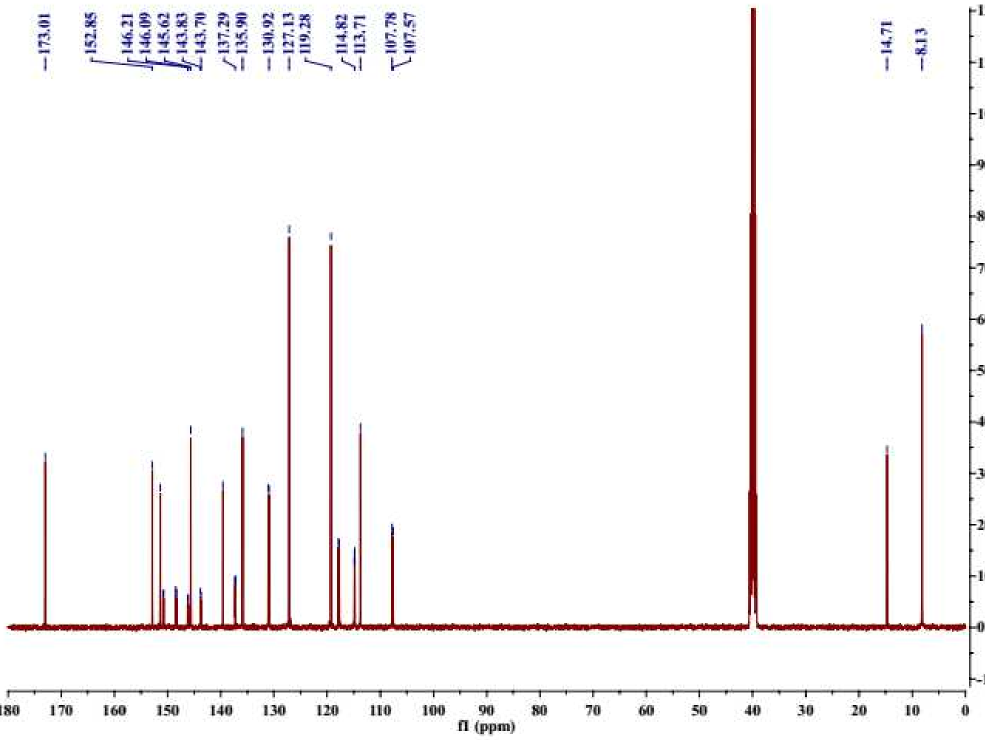

mp:280~282℃, HRMS *m/z* calcd for $C_{22}H_{18}F_2N_4O_2$ ($[M+H]^+$) 409.1476, found 409.1456. ^1H NMR (400 MHz, DMSO-d_6) δ 10.87 (s, 1H), 8.94 (s, 1H), 8.89 (s, 1H), 8.62 (d, *J* = 2.1 Hz, 1H), 8.14 (d, *J* = 8.7 Hz, 1H), 8.04 (m, *J* = 8.7, 2.3 Hz, 1H), 7.72 – 7.62 (m, 2H), 7.56 (d, *J* = 8.6 Hz, 2H), 7.35 (m, *J* = 19.6, 9.3 Hz, 1H), 7.14 (d, *J* = 9.0 Hz, 1H), 2.09 – 1.98 (m, 1H), 0.82 (m, *J* = 14.9, 6.4 Hz, 4H).^{13}C NMR (101 MHz, DMSO-d_6) δ 173.01, 152.85, 151.40, 150.87, 150.74, 148.46, 148.33, 146.21, 146.09, 145.62, 143.83, 143.70, 139.56, 137.31, 137.29, 137.22, 137.20,

135.90, 131.06, 130.92, 127.13, 119.28, 117.88, 117.70, 114.88, 114.85, 114.82, 114.79, 113.71, 107.78, 107.57, 14.71, 8.13.

N-(5-(4-(3-(3-chloro-4-methylphenyl)ureido)phenyl)pyridin-2-yl)cyclopropanecarboxamide(CDAU-5)

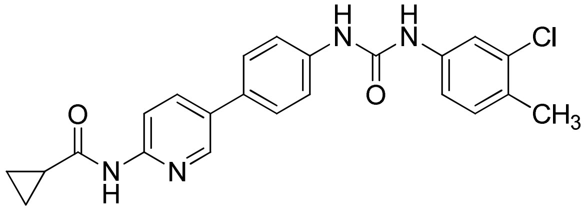

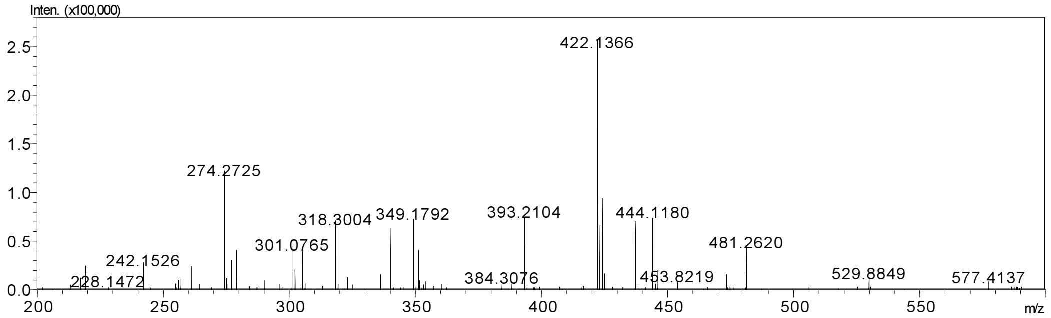

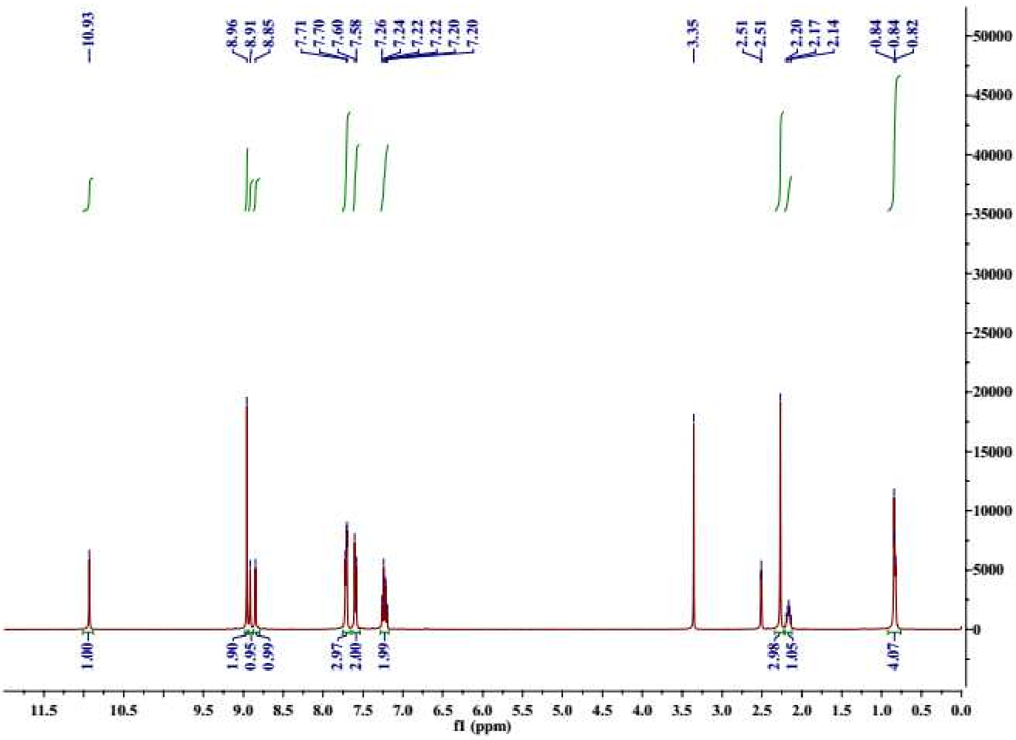

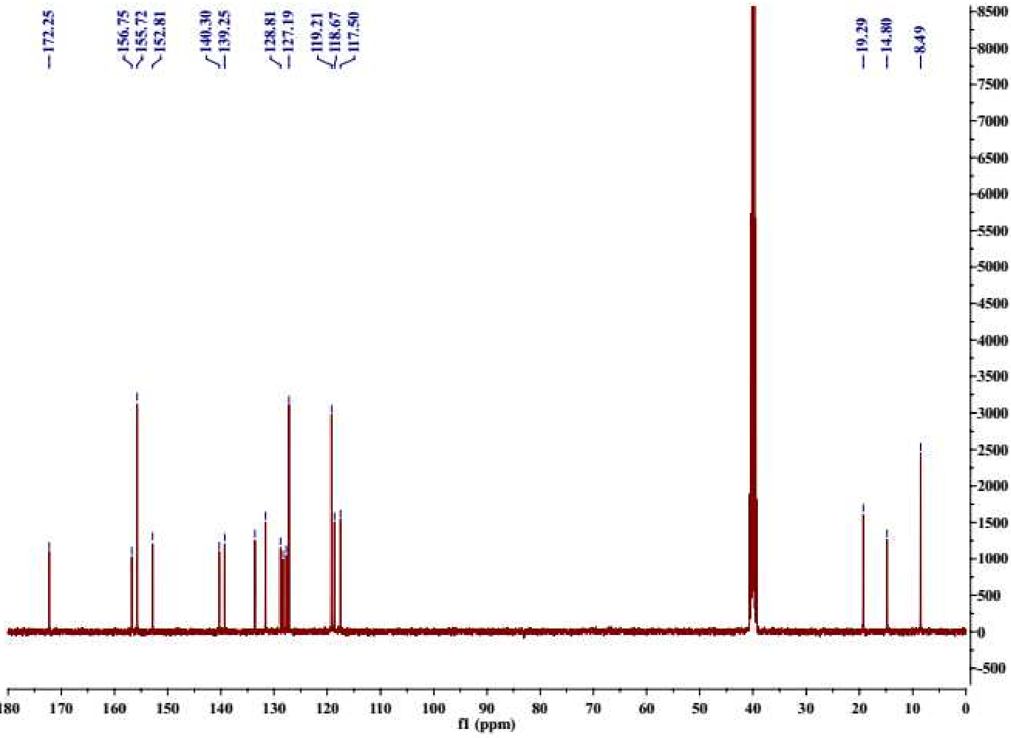

mp:229~231℃, HRMS *m/z* calcd for $C_{23}H_{21}ClN_4O_2$ ($[M+2H]^+$) 422.1510, found 422.1366. ^{1}H NMR (400 MHz, DMSO-d_6) δ 10.93 (s, 1H), 8.96 (s, 2H), 8.91 (s, 1H), 8.85 (s, 1H), 7.71 (m, J = 5.3, 3.3 Hz, 3H), 7.59 (d, J = 8.7 Hz, 2H), 7.22 (m, J = 8.3, 5.1 Hz, 2H), 2.27 (s, 3H), 2.22 – 2.12 (m, 1H), 0.92 – 0.76 (m, 4H). ^{13}C NMR (101 MHz, DMSO-d_6) δ 172.25, 156.75, 155.72, 152.81, 140.30, 139.25, 133.59, 131.63, 128.81, 128.34, 127.71, 127.19, 119.21, 118.67, 117.50, 19.29, 14.80, 8.49.

N-(5-(4-(3-(3-chlorophenyl)ureido)phenyl)pyridin-2-yl)cyclopropanecarboxamide (CDAU-6)

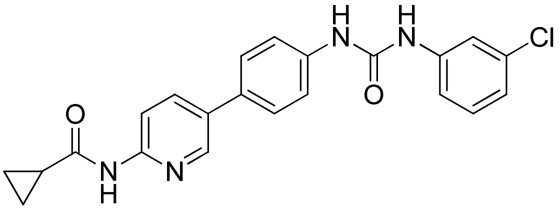

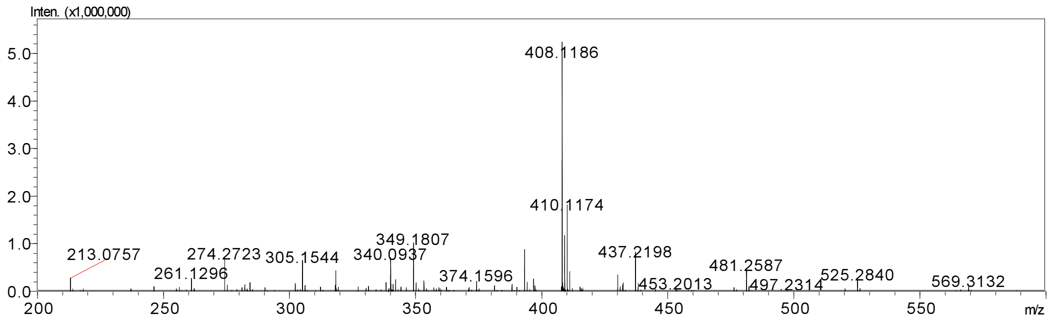

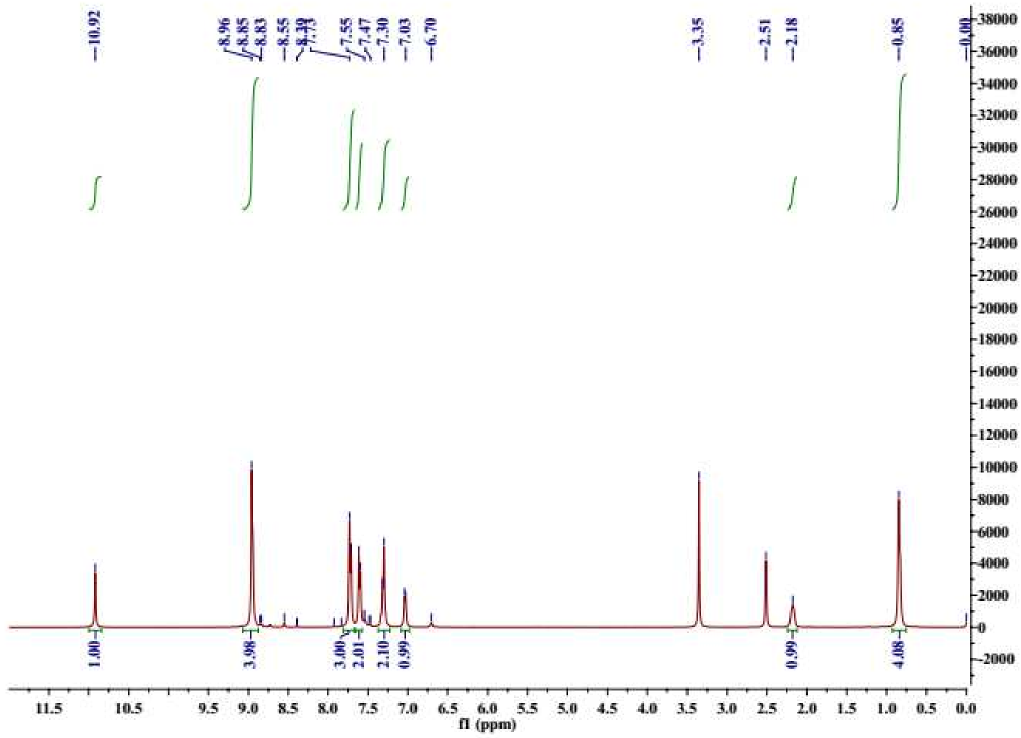

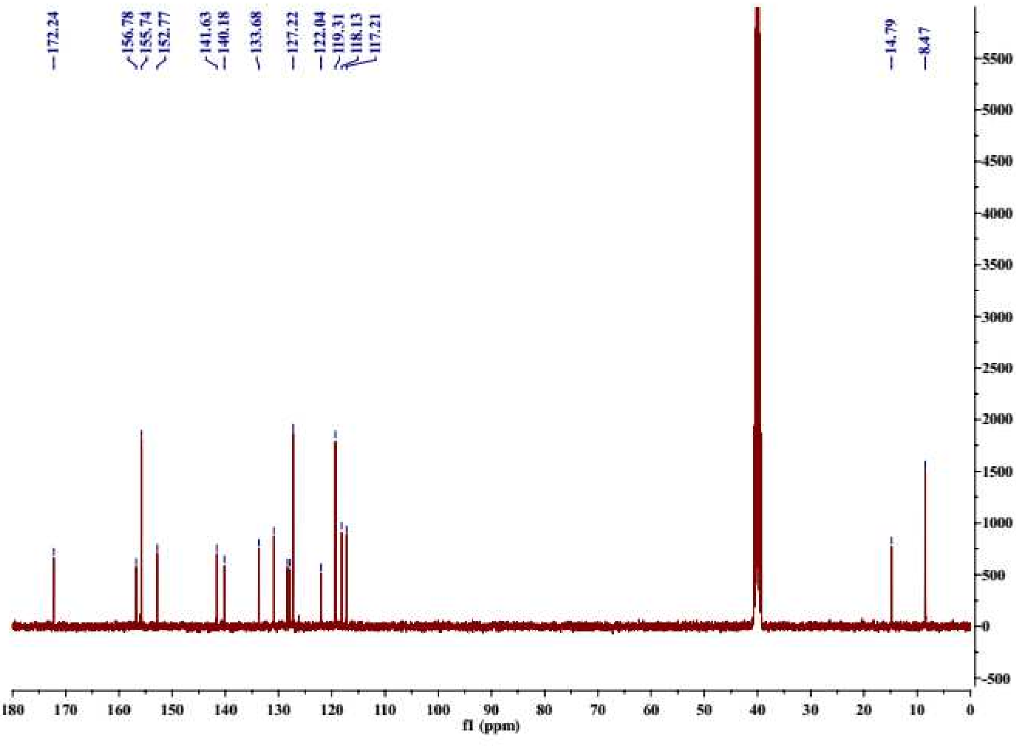

mp:270~272℃, HRMS *m/z* calcd for $C_{22}H_{19}ClN_4O_2$ ($[M+2H]^+$) 408.1197, found 408.1186. ^1H NMR (400 MHz, DMSO-d_6) δ 10.92 (s, 1H), 8.96 (s, 4H), 7.72 (d, *J* = 8.2 Hz, 3H), 7.60 (d, *J* = 8.2 Hz, 2H), 7.31 (d, *J* = 6.2 Hz, 2H), 7.04 (d, *J* = 6.0 Hz, 1H), 2.18 (s, 1H), 0.85 (s, 4H).^{13}C NMR (101 MHz, DMSO-d_6) δ 172.24, 156.78, 155.74, 152.77, 141.63, 140.18, 133.68, 130.87, 128.33, 127.86, 127.22, 122.04, 119.31, 118.13, 117.21, 14.79, 8.47.

N-(5-(4-(3-(2-fluorophenyl)ureido)phenyl)pyridin-2-yl)cyclopropanecarboxamide (CDAU-7)

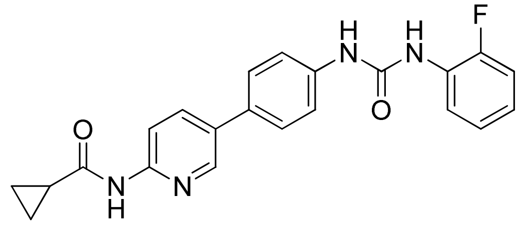

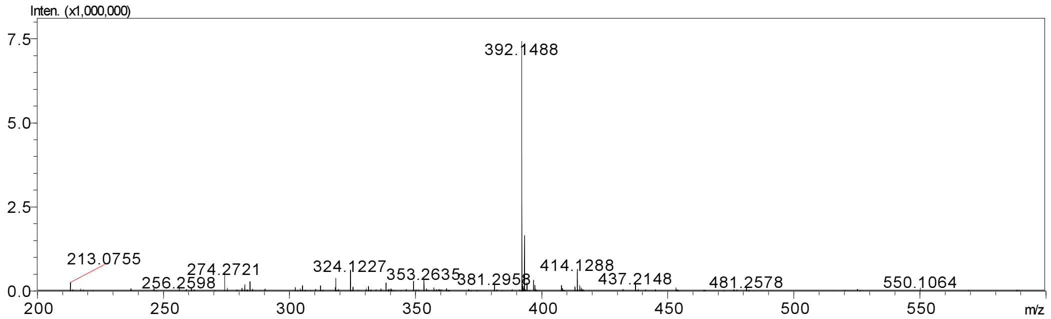

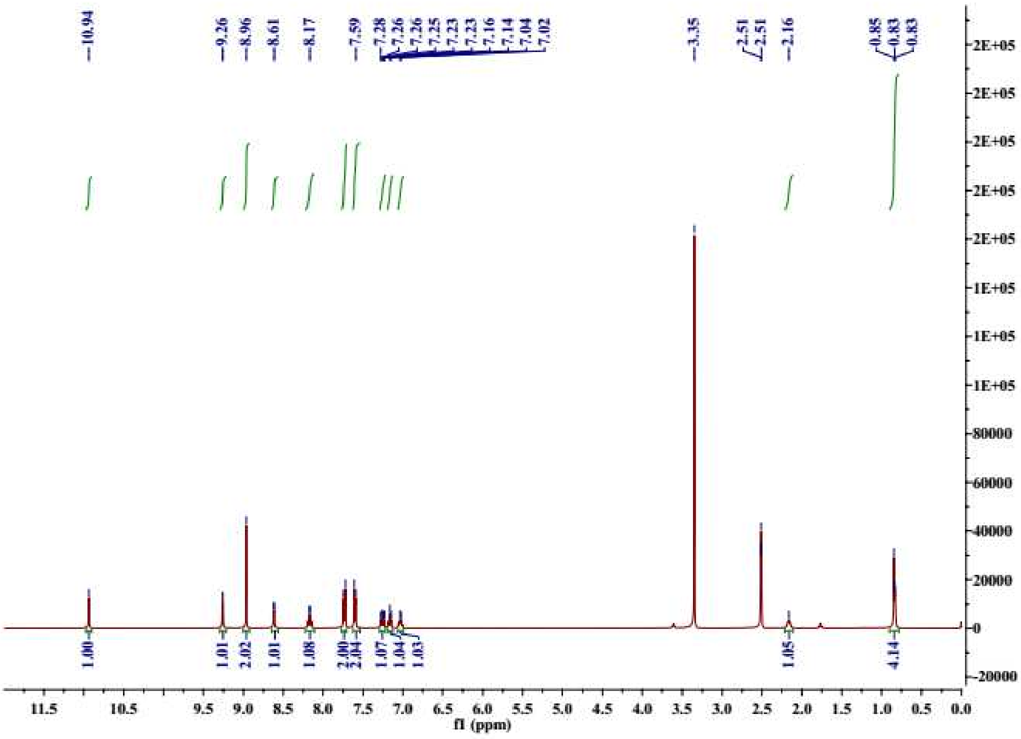

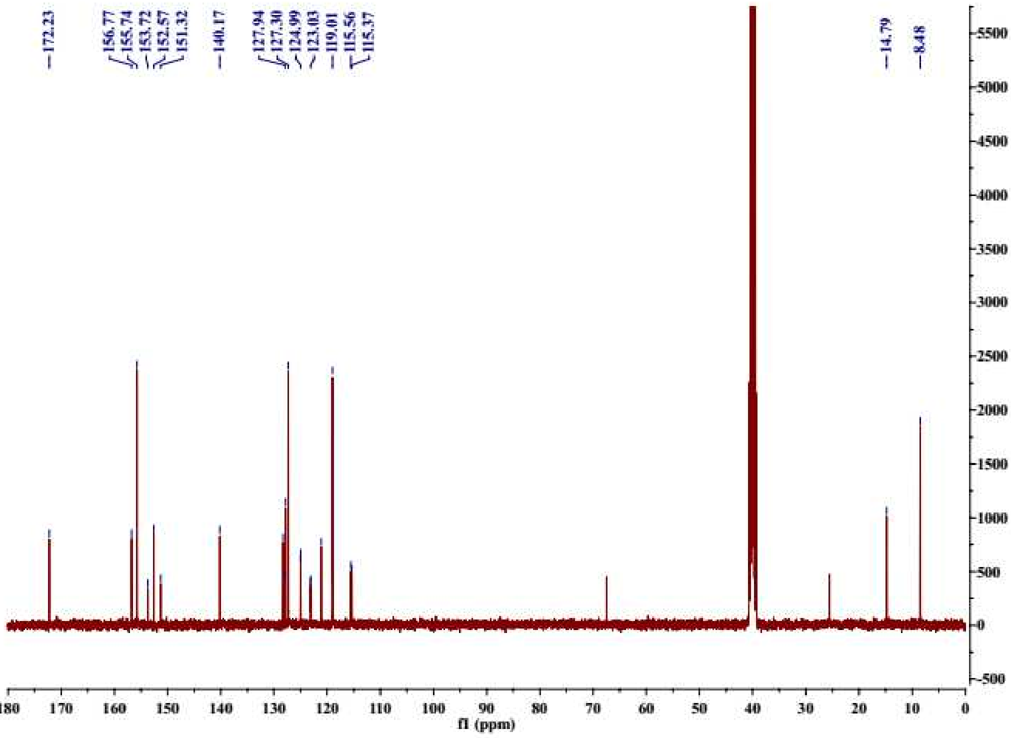

mp:222~224℃, HRMS *m/z* calcd for $C_{22}H_{19}FN_4O_2$ ($[M+2H]^+$) 392.1492, found m/z =392.1148. ^{1}H NMR (400 MHz, DMSO-d_6) δ 10.94 (s, 1H), 9.26 (s, 1H), 8.96 (s, 2H), 8.62 (d, J = 2.2 Hz, 1H), 8.17 (d, J = 1.3 Hz, 1H), 7.73 (d, J = 8.7 Hz, 2H), 7.60 (d, J = 8.7 Hz, 2H), 7.30 – 7.22 (m, 1H), 7.15 (d, J = 7.6 Hz, 1H), 7.03 (d, J = 7.0 Hz, 1H), 2.16 (s, 1H), 0.90 – 0.78 (m, 4H). ^{13}C NMR (101 MHz, DMSO-d_6) δ 172.23, 156.77, 155.74, 153.72, 152.57, 151.32, 140.17, 128.31, 127.94, 127.83, 127.30, 125.02, 124.99, 123.11, 123.03, 121.10, 119.01, 115.56, 115.37, 14.79, 8.48.

N-(5-(4-(3-(4-(trifluoromethoxy)phenyl)ureido)phenyl)cyclopropanecarboxamide (CDAU-8)

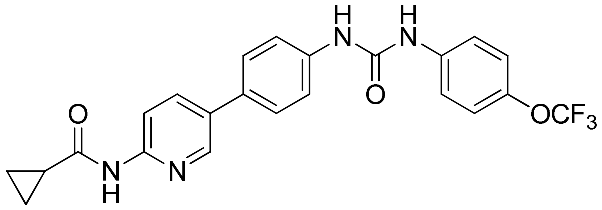

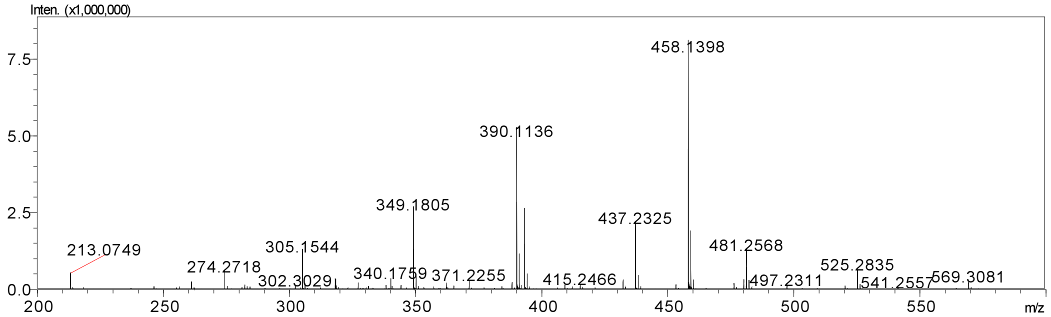

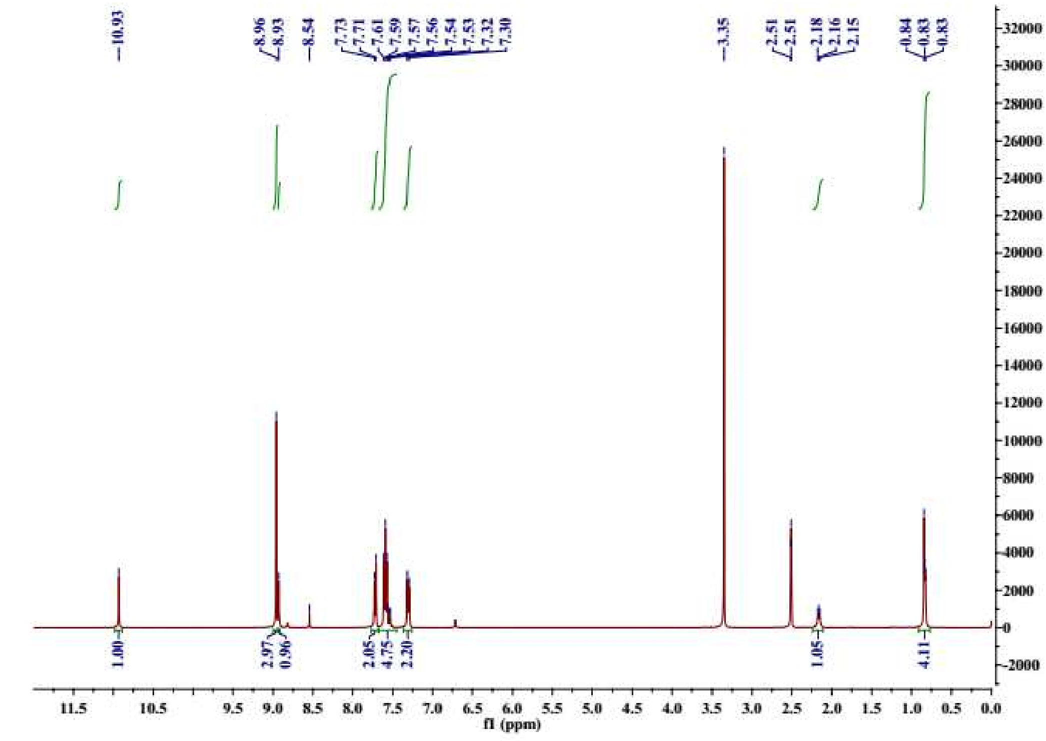

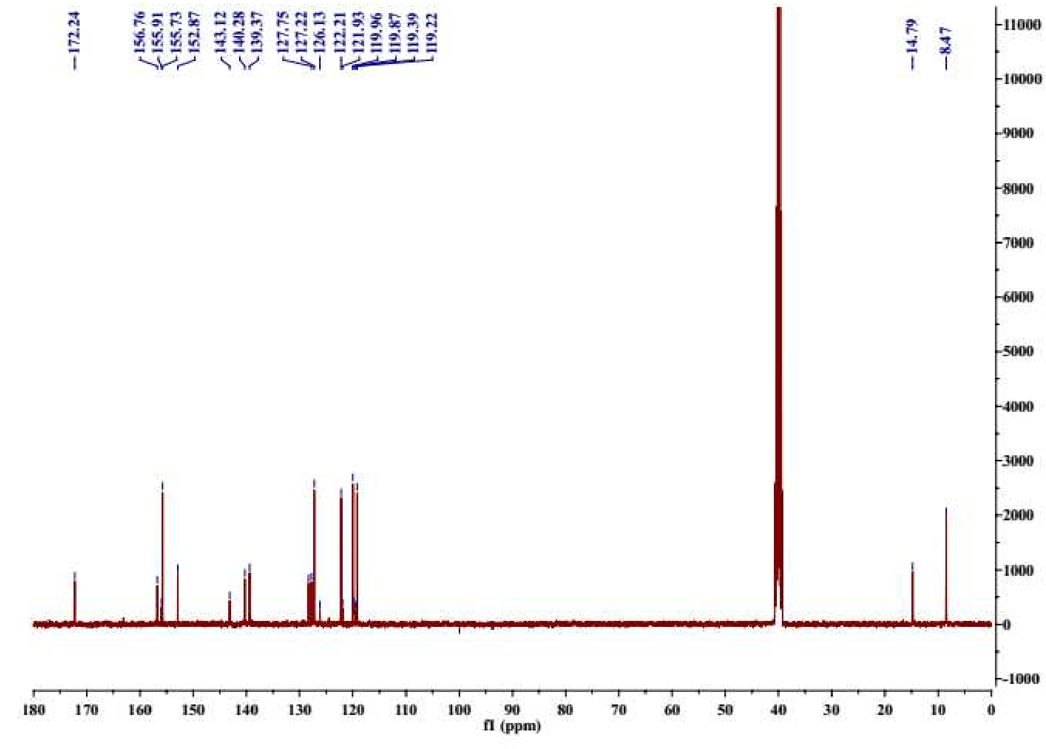

mp:236~238℃, HRMS *m/z* calcd for $C_{23}H_{19}F_3N_4O_3$ ($[M+2H]^+$) 458.1409, found 458.1398. ^{1}H NMR (400 MHz, DMSO-d_6) δ 10.93 (s, 1H), 8.96 (s, 3H), 8.93 (s, 1H), 7.72 (d, *J* = 8.7 Hz, 2H), 7.67 – 7.45 (m, 4H), 7.31 (d, *J* = 8.6 Hz, 2H), 2.24 – 2.11 (m, 1H), 0.91 – 0.77 (m, 4H).^{13}C NMR (101 MHz, DMSO-d_6) δ 172.24, 156.76, 155.91, 155.73, 152.87, 143.12, 140.28, 139.37, 128.33, 127.75, 127.22, 126.13, 122.21, 121.93, 119.96, 119.87, 119.39, 119.22, 14.79, 8.47.

N-(5-(4-(3-(tert-butyl)ureido)phenyl)pyridin-2-yl)cyclopropanecarboxamide(CDAU-9)

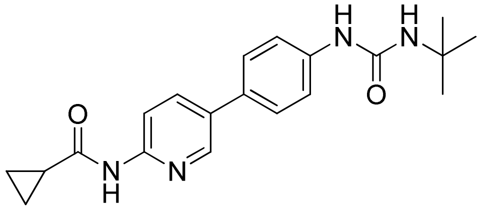

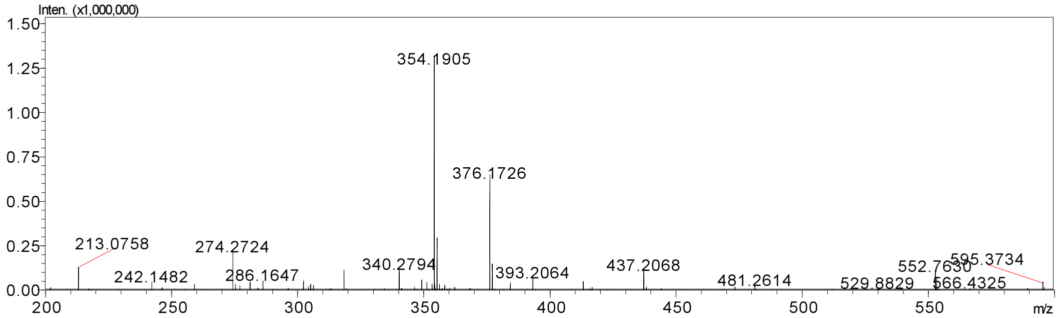

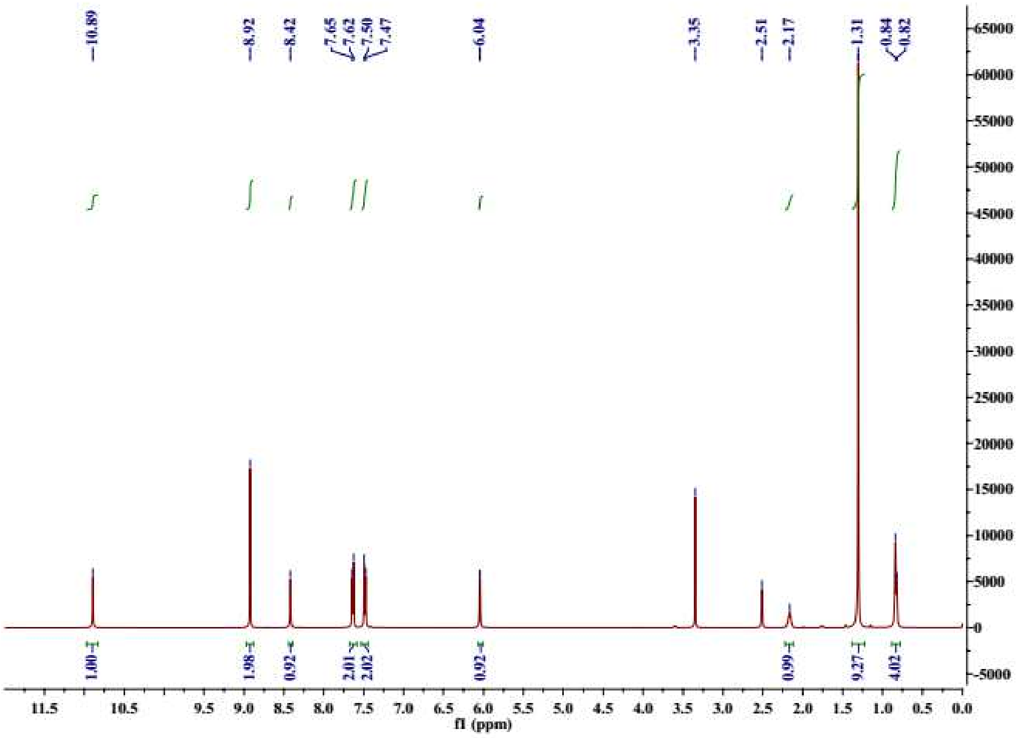

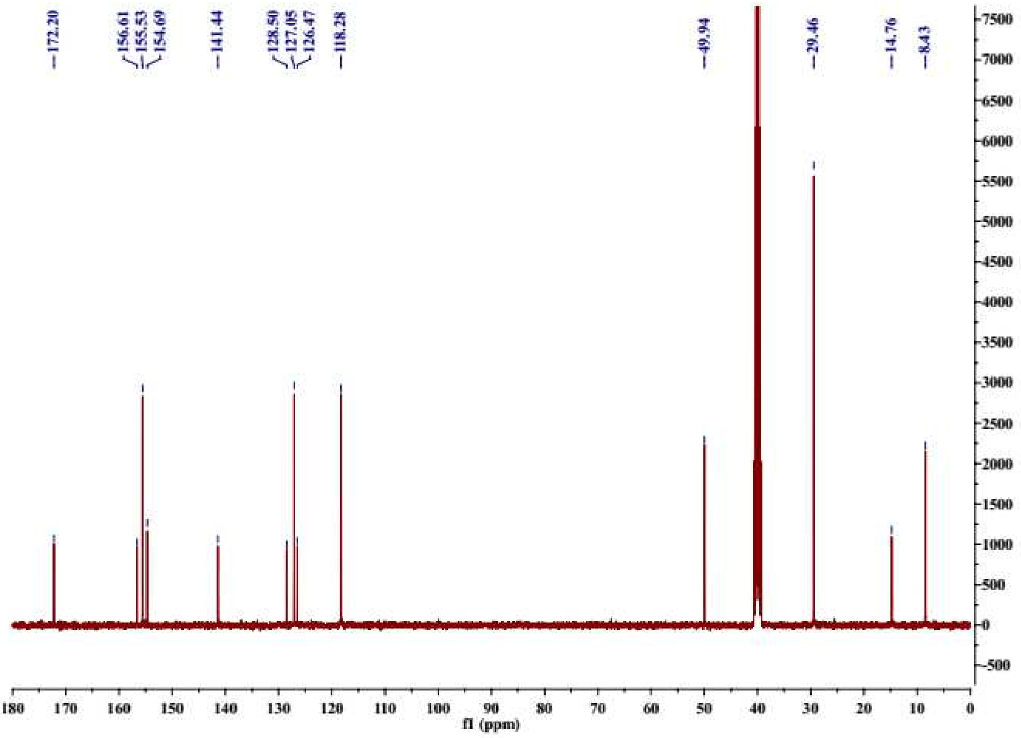

mp:214~216℃, HRMS *m/z* calcd for $C_{20}H_{24}N_4O_2$ ($[M+H]^+$) 354.1899, found 354.1905. ^{1}H NMR (400 MHz, DMSO-d_6) δ 10.89 (s, 1H), 8.92 (s, 2H), 8.42 (s, 1H), 7.63 (d, J = 8.6 Hz, 2H), 7.49 (d, J = 8.6 Hz, 2H), 6.04 (s, 1H), 2.17 (s, 1H), 1.31 (s, 9H), 0.83 (d, J = 7.9 Hz, 4H).^{13}C NMR (101 MHz, DMSO-d_6) δ 172.20, 156.61, 155.53, 154.69, 141.44, 128.50, 127.05, 126.47, 118.28, 49.94, 29.46, 14.76, 8.43.

N-(5-(4-(3-(2-(dimethylamino)ethyl)ureido)phenyl)pyridin-2-yl)cyclopropanecarboxamide(CDAU-10)

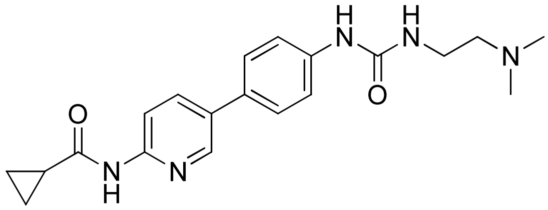

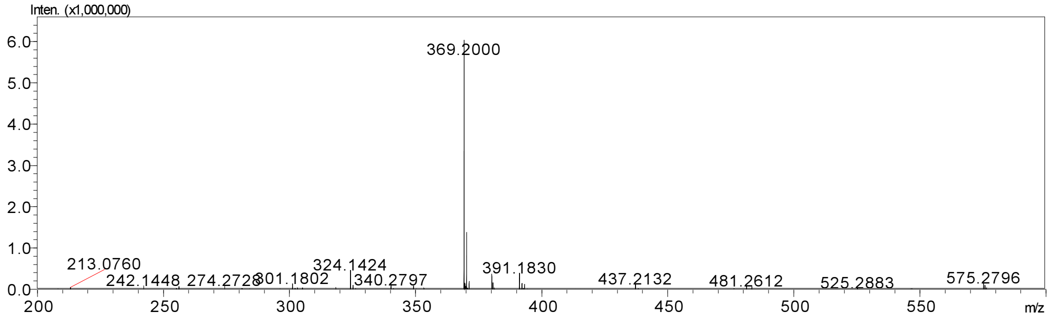

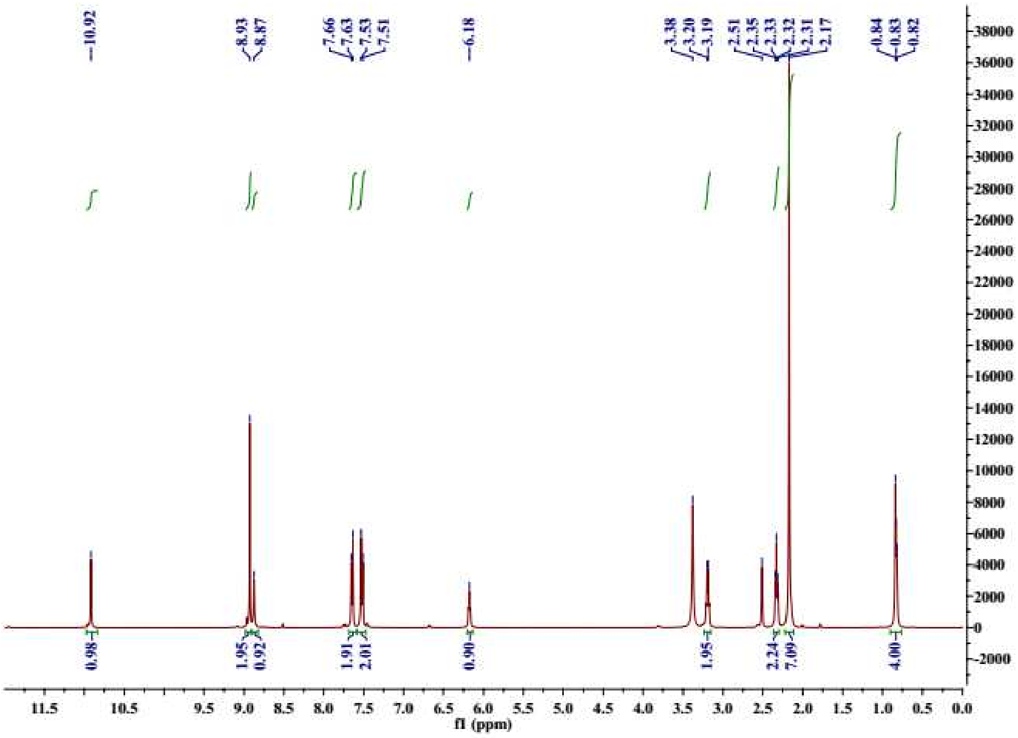

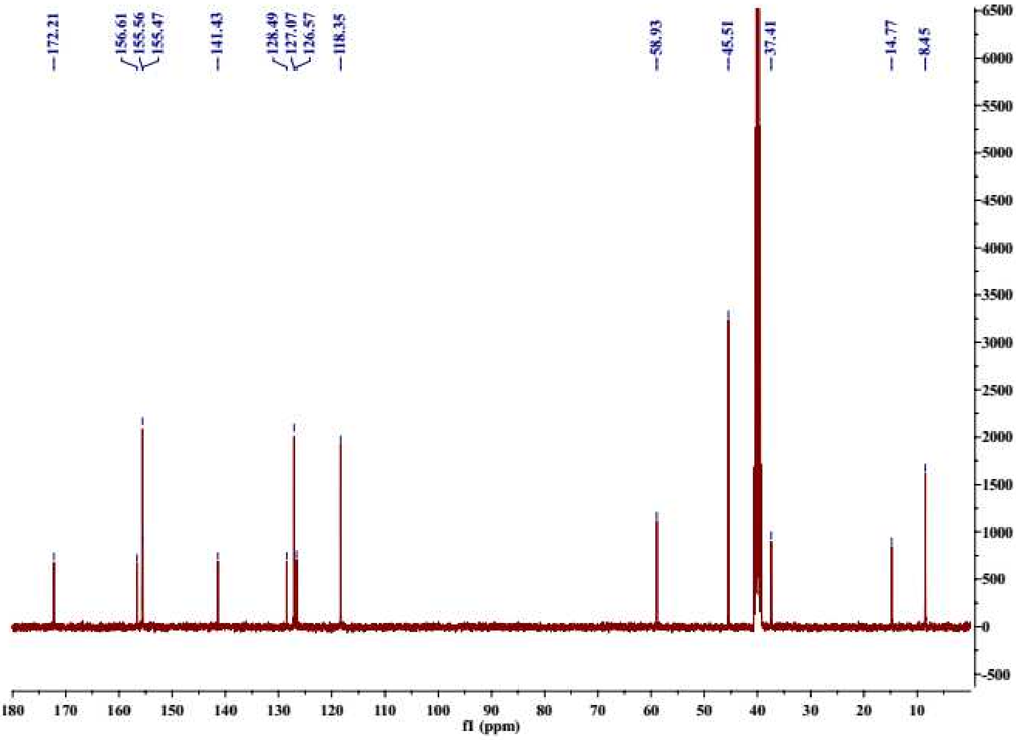

mp:214~216℃, HRMS *m/z* calcd for $C_{20}H_{25}N_5O_2$ ($[M+2H]^+$) 369.2008, found 369.2000. ^1H NMR (400 MHz, DMSO-d_6) δ 10.92 (s, 1H), 8.93 (s, 2H), 8.87 (s, 1H), 7.65 (d, J = 8.6 Hz, 2H), 7.52 (d, J = 8.6 Hz, 2H), 6.18 (s, 1H), 3.19 (d, J = 5.6 Hz, 2H), 2.32 (m, J = 11.0, 4.8 Hz, 2H), 2.17 (s, 7H), 0.90 – 0.76 (m, 4H). ^{13}C NMR (101 MHz, DMSO-d_6) δ 172.21, 156.61, 155.56, 155.47, 141.43, 128.49, 127.07, 126.57, 118.35, 58.93, 45.51, 37.41, 14.77, 8.45.

N-(4-(6-(cycloropanecarboxamido)pyridin-3-yl)phenyl)-2,2-dimethylhydrazinecarboxamide(CDAU-11)

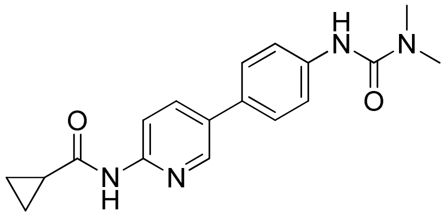

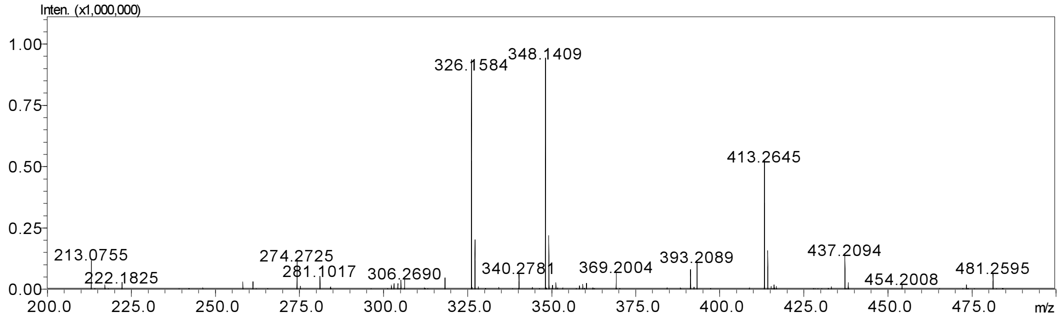

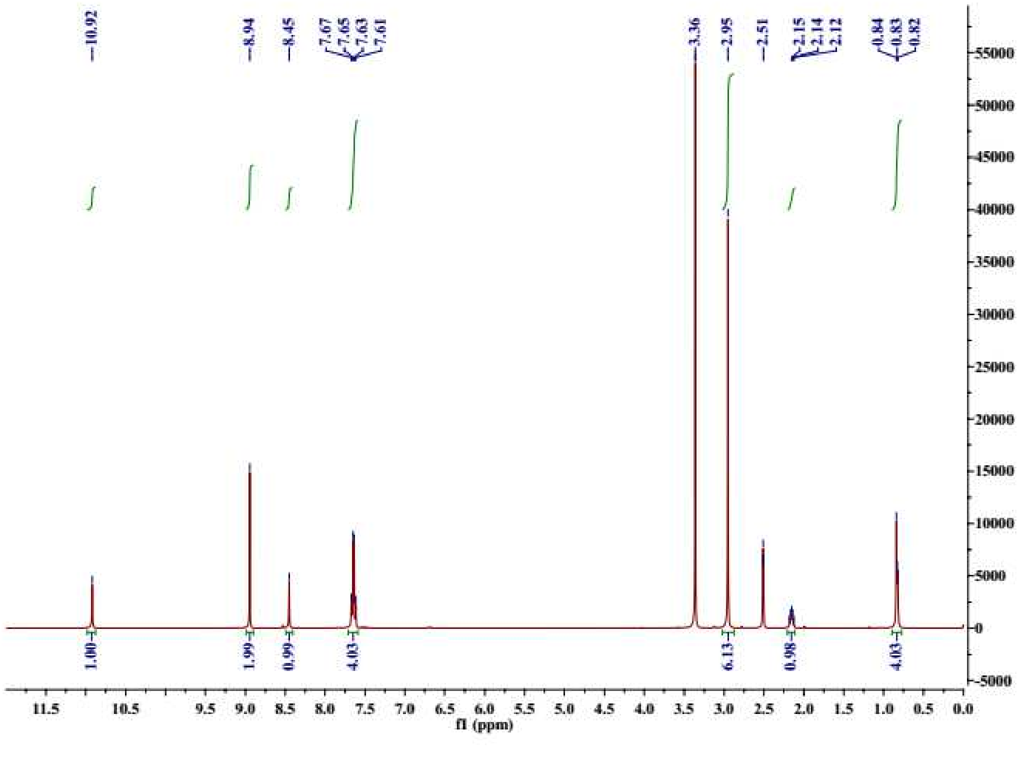

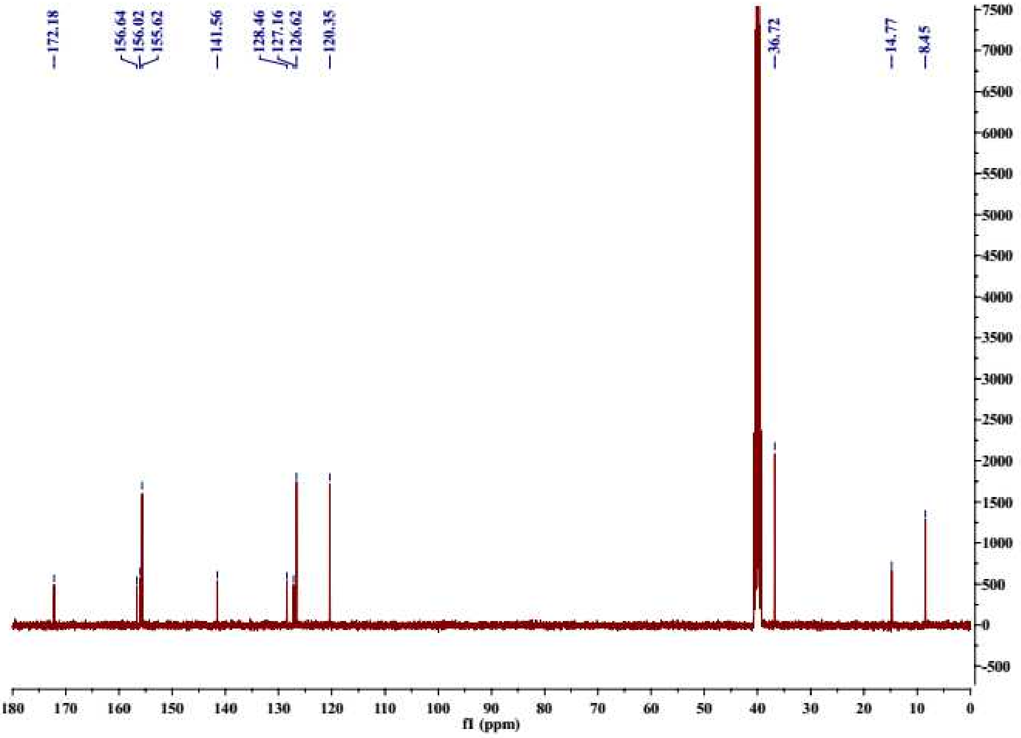

mp:230~232℃, HRMS *m/z* calcd for $C_{18}H_{20}N_4O_2$ ($[M+2H]^+$) 326.1586, found 326.1584. ^{1}H NMR (400 MHz, DMSO-*d6*) δ 10.92 (s, 1H), 8.94 (s, 2H), 8.45 (s, 1H), 7.64 (q, *J* = 8.9 Hz, 4H), 2.95 (s, 6H), 2.21 – 2.11 (m, 1H), 0.89 – 0.77 (m, 4H).^{13}C NMR (101 MHz, DMSO-*d6*) δ 172.18, 156.64, 156.02, 155.62, 141.56, 128.46, 127.16, 126.62, 120.35, 36.72, 14.77, 8.45.

N-(6-(4-(3-(4-chloro-3-(trifluoromethyl)phenyl)ureido)phenyl)pyridin-2-yl)cyclopropanecarboxamide(CDAU-12)

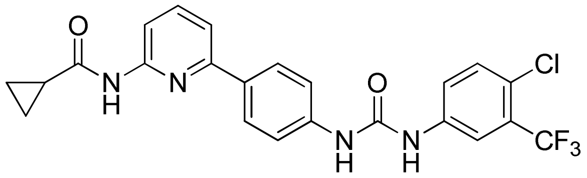

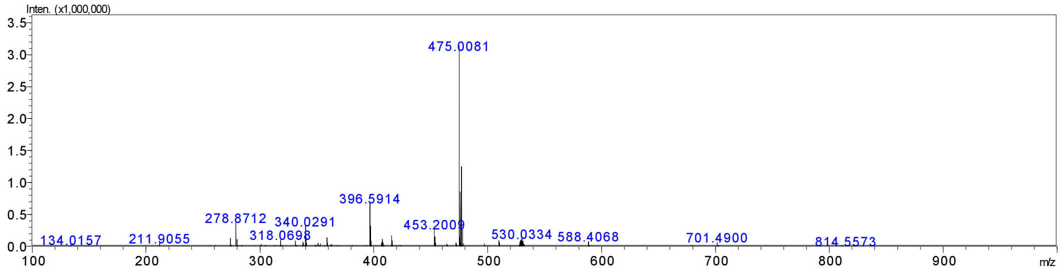

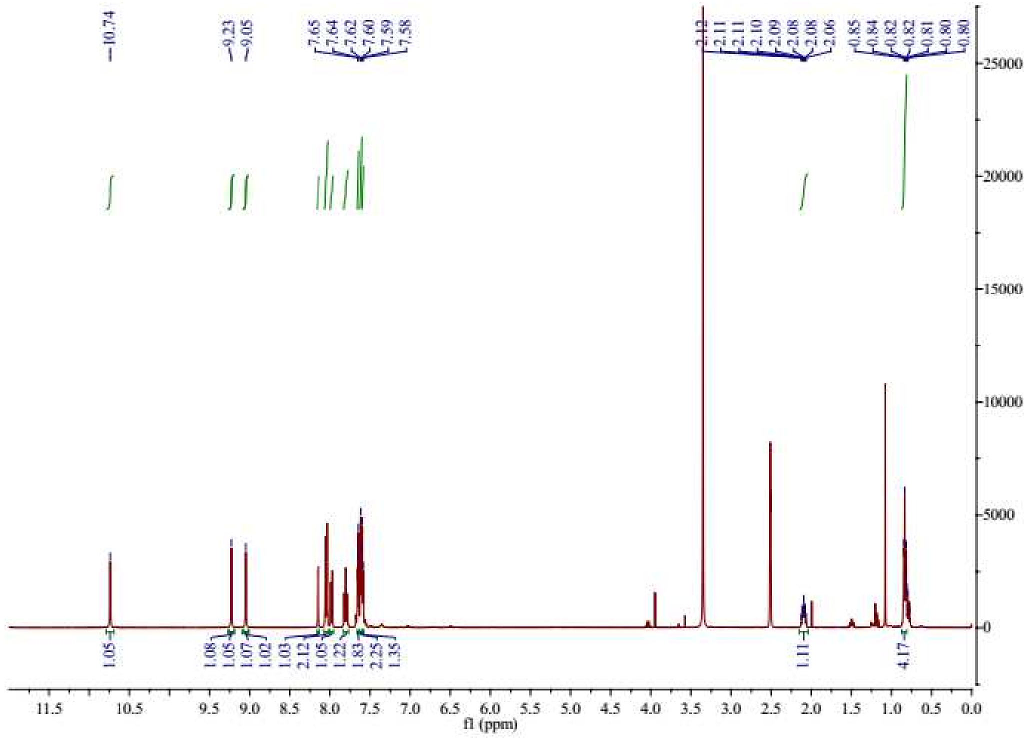

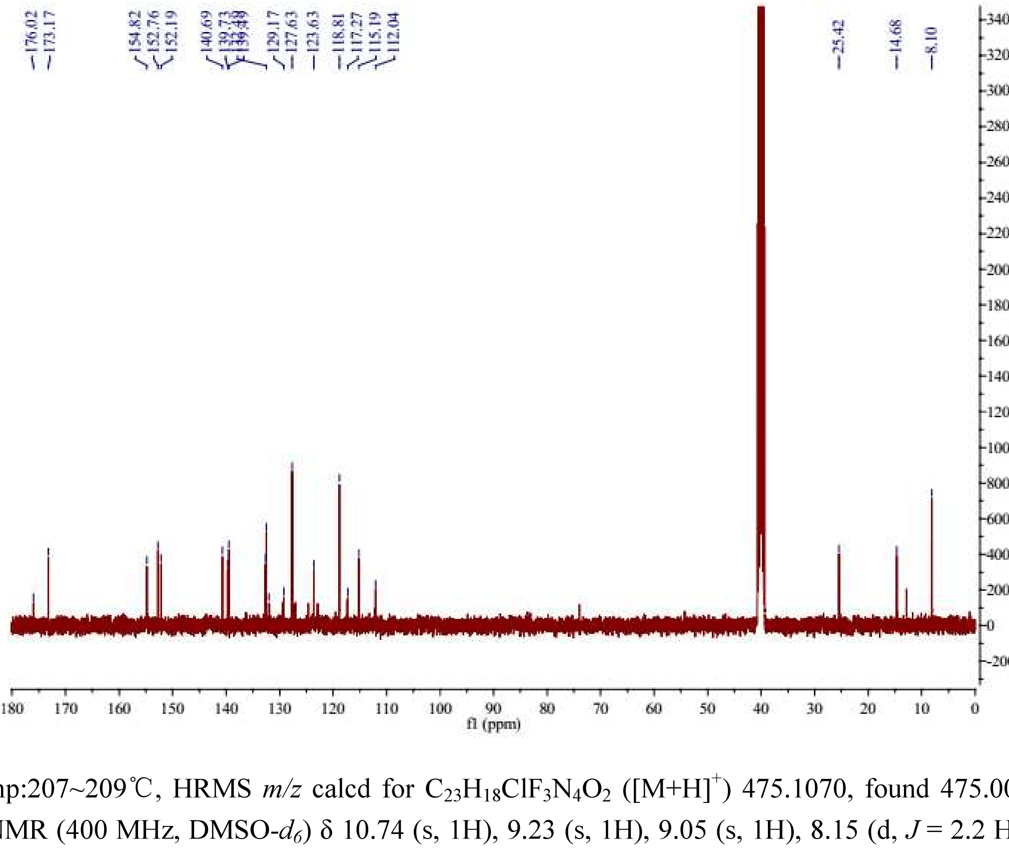

mp:207~209℃, HRMS *m/z* calcd for $C_{23}H_{18}ClF_3N_4O_2$ ($[M+H]^+$) 475.1070, found 475.0081. ^{1}H NMR (400 MHz, DMSO-d_6) δ 10.74 (s, 1H), 9.23 (s, 1H), 9.05 (s, 1H), 8.15 (d, *J* = 2.2 Hz, 1H), 8.04 (d, *J* = 8.8 Hz, 2H), 7.98 (d, *J* = 8.2 Hz, 1H), 7.80 (t, *J* = 7.9 Hz, 1H), 7.65 (d, *J* = 3.2 Hz, 2H), 7.61 (d, *J* = 5.4 Hz, 2H), 7.59 (d, *J* = 4.1 Hz, 1H), 2.09 (m, *J* = 7.4, 5.1 Hz, 1H), 0.83 (m, *J* = 9.0, 3.2 Hz, 4H).^{13}C NMR (101 MHz, DMSO-d_6) δ 176.02, 173.17, 154.82, 152.76, 152.19, 140.69,

139.73, 139.49, 132.62, 132.49, 132.00, 129.17, 127.63, 123.63, 118.81, 117.27, 115.19, 112.04, 25.42, 14.68, 8.10.

N-(6-(4-(3-(4-chloro-3-methylphenyl)ureido)phenyl)pyridin-2-yl)cyclopropanecarboxamide(CDAU-13)

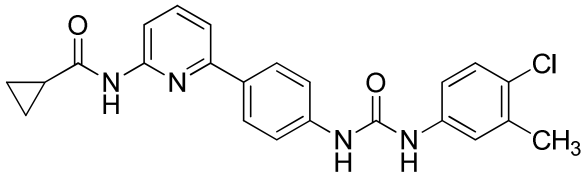

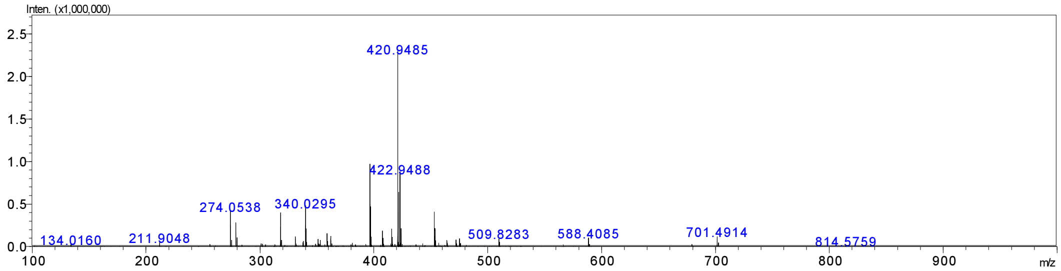

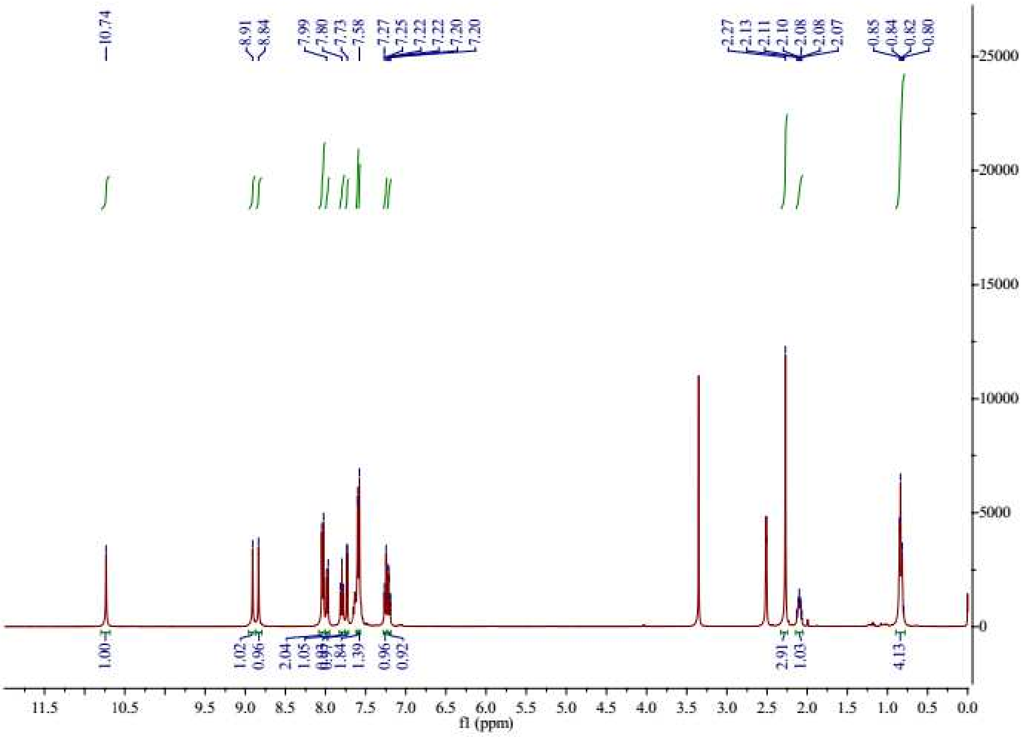

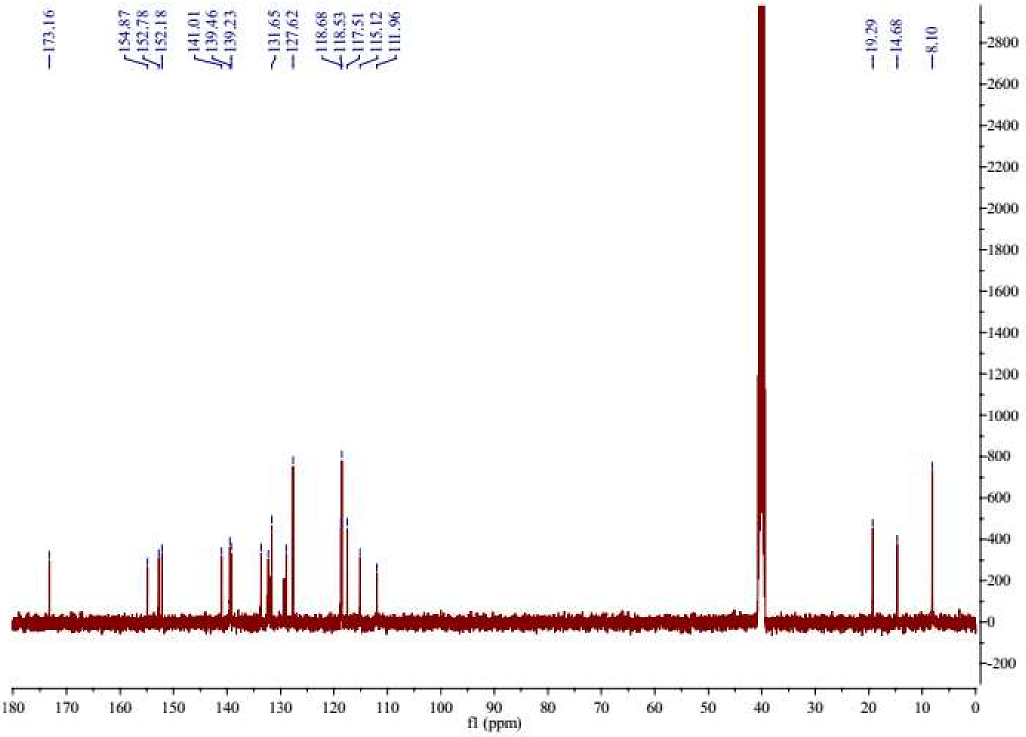

mp:272~274℃, HRMS *m/z* calcd for $C_{23}H_{21}ClN_4O_2$ ($[M+H]^+$) 420.9353, found 420.9485. ^1H NMR (400 MHz, DMSO-d_6) δ 10.74 (s, 1H), 8.91 (s, 1H), 8.84 (s, 1H), 8.03 (d, J = 8.7 Hz, 2H), 7.98 (d, J = 8.2 Hz, 1H), 7.80 (t, J = 7.9 Hz, 1H), 7.73 (d, J = 1.9 Hz, 1H), 7.60 (d, J = 1.2 Hz, 2H), 7.58 (s, 1H), 7.26 (d, J = 8.4 Hz, 1H), 7.21 (m, J = 8.3, 2.0 Hz, 1H), 2.27 (s, 3H), 2.15 – 2.04 (m, 1H), 0.83 (m, J = 14.6, 6.4 Hz, 4H).^{13}C NMR (101 MHz, DMSO-d_6) δ 173.16, 154.87, 152.78, 152.18, 141.01, 139.46, 139.23, 133.61, 132.30, 131.65, 128.85, 127.62, 118.68, 118.53, 117.51, 115.12, 111.96, 19.29, 14.68, 8.10.

N-(6-(4-(3-(4-fluorophenyl)ureido)phenyl)pyridin-2-yl)cyclopropanecarboxamide(CDAU-14)

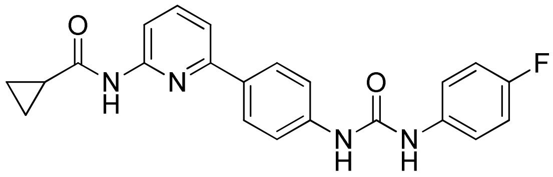

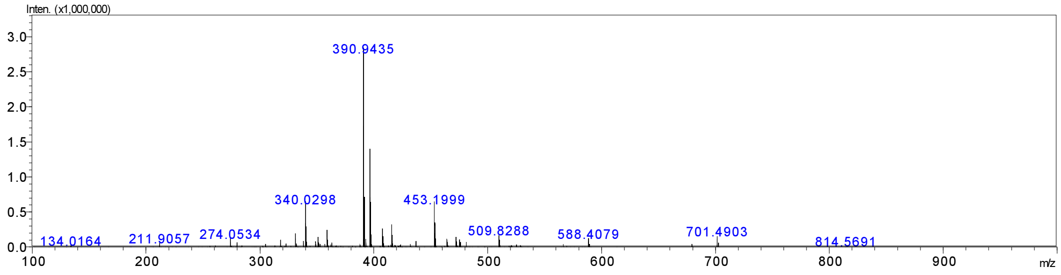

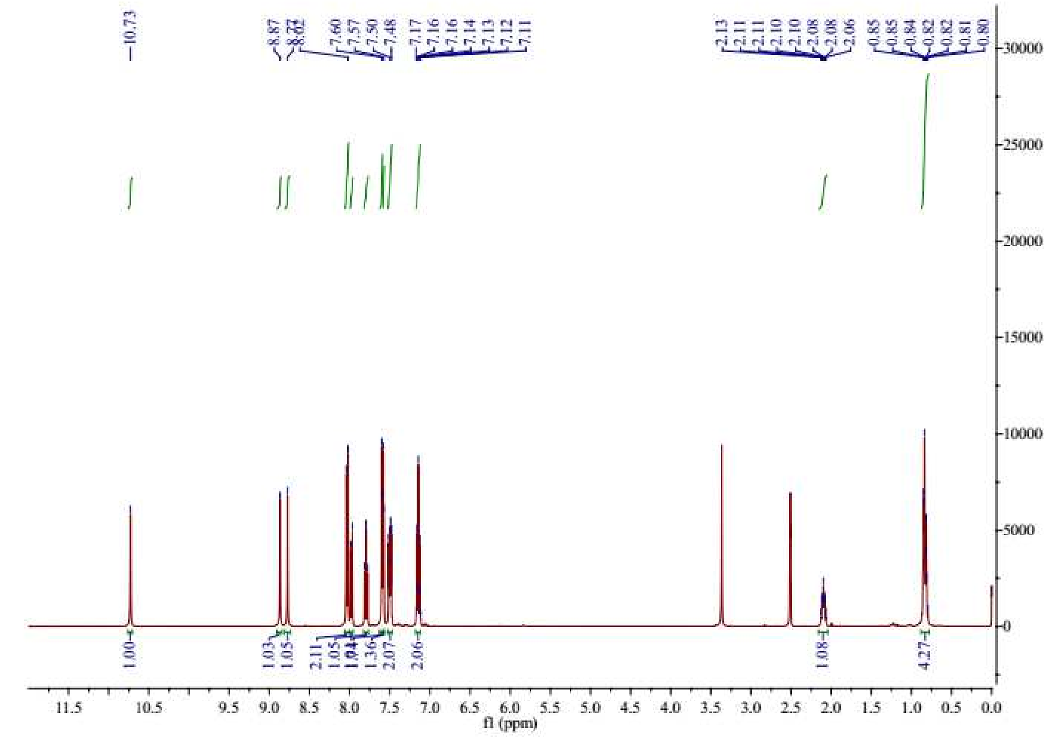

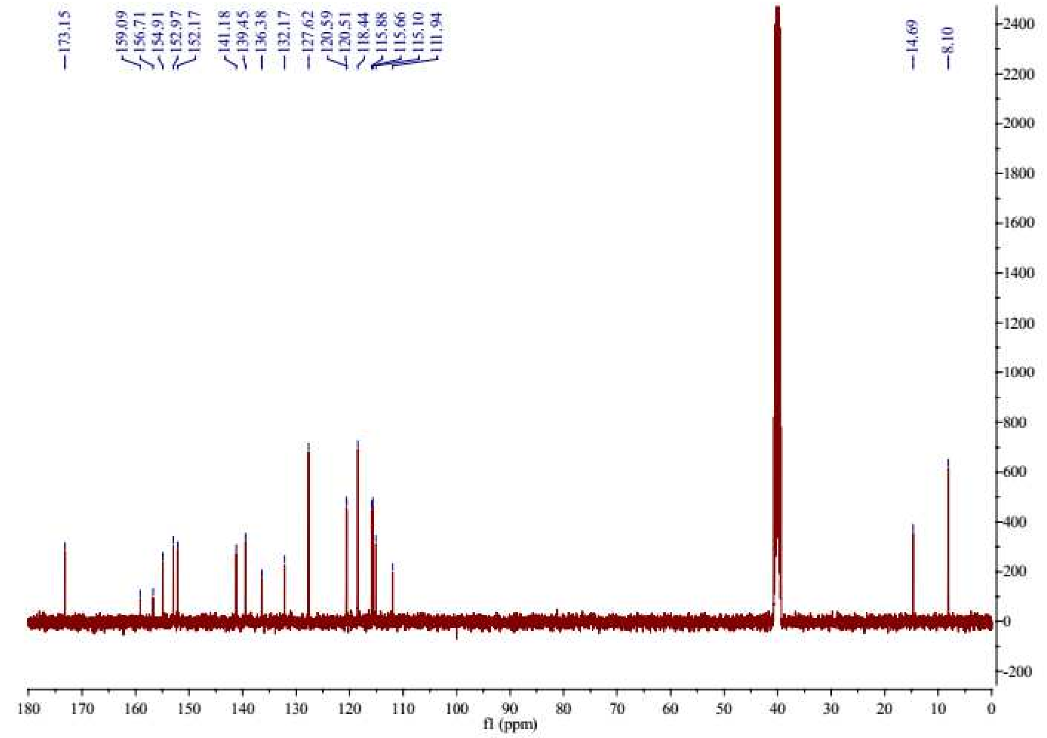

mp:278~280℃, HRMS *m/z* calcd for $C_{22}H_{19}FN_4O_2$ ($[M]^+$) 390.1492, found 390.9435. ^1H NMR (400 MHz, DMSO-d_6) δ 10.73 (s, 1H), 8.87 (s, 1H), 8.77 (s, 1H), 8.03 (d, *J* = 8.8 Hz, 2H), 7.98 (d, *J* = 8.2 Hz, 1H), 7.80 (t, *J* = 7.9 Hz, 1H), 7.59 (d, *J* = 2.7 Hz, 2H), 7.57 (d, *J* = 1.5 Hz, 1H), 7.52 – 7.47 (m, 2H), 7.18 – 7.11 (m, 2H), 2.10 (m, *J* = 7.5, 5.0 Hz, 1H), 0.87 – 0.80 (m, 4H). ^{13}C NMR (101 MHz, DMSO-d_6) δ 173.15, 159.09, 156.71, 154.91, 152.97, 152.17, 141.18, 139.45, 136.38, 132.17, 127.62, 120.59, 120.51, 118.44, 115.88, 115.66, 115.10, 111.94, 14.69, 8.10.

N-(6-(4-(3-(4-(tert-butyl)phenyl)ureido)phenyl)pyridin-2-yl)cyclopropanecarboxamide(CDAU-15)

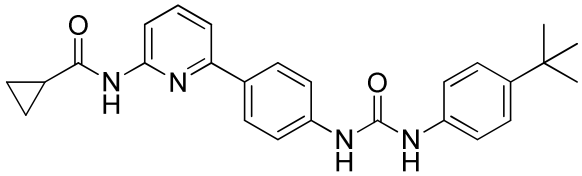

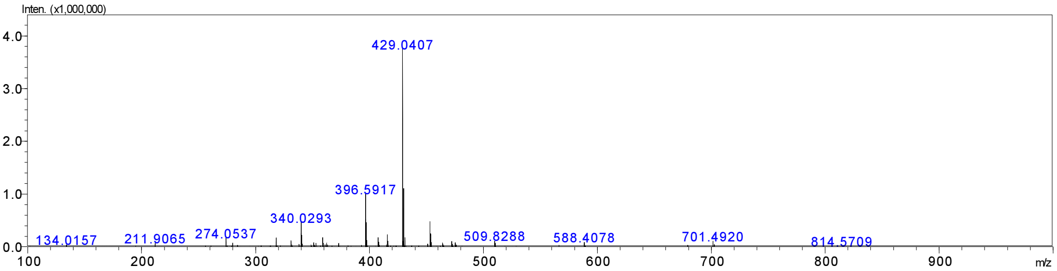

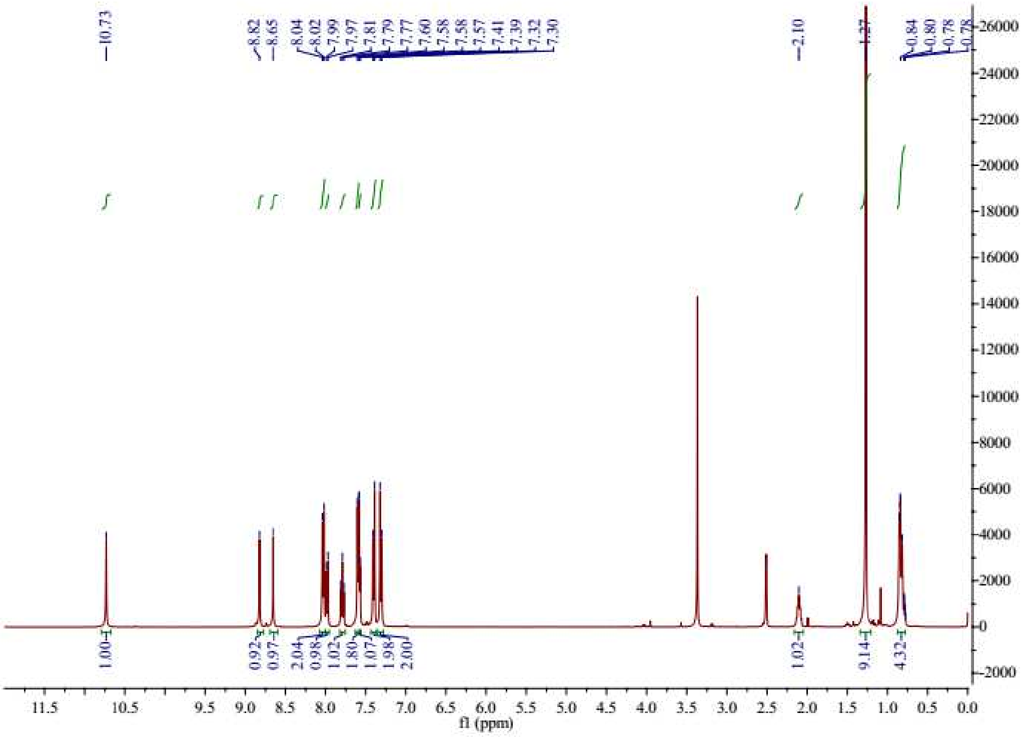

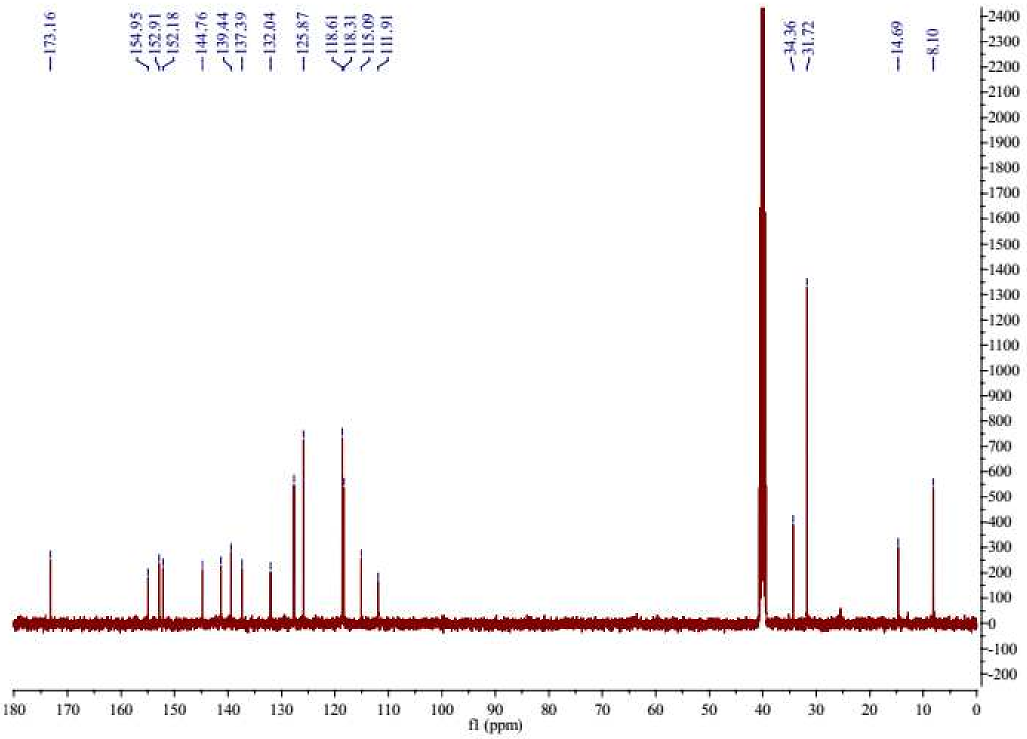

mp:209~211℃, HRMS *m/z* calcd for $C_{26}H_{28}N_4O_2$ ($[M]^+$) 428.2212, found 429.0407. ^{1}H NMR (400 MHz, DMSO-d_6) δ 10.73 (s, 1H), 8.82 (s, 1H), 8.65 (s, 1H), 8.03 (d, *J* = 8.7 Hz, 2H), 7.98 (d, *J* = 8.2 Hz, 1H), 7.79 (t, *J* = 7.9 Hz, 1H), 7.59 (d, *J* = 7.0 Hz, 2H), 7.57 (d, *J* = 5.8 Hz, 1H), 7.40 (d, *J* = 8.7 Hz, 2H), 7.31 (d, *J* = 8.7 Hz, 2H), 2.19 – 2.04 (m, 1H), 1.27 (s, 9H), 0.90 – 0.77 (m, 4H).^{13}C NMR (101 MHz, DMSO-d_6) δ 173.16, 154.95, 152.91, 152.18, 144.76, 141.31, 139.44, 137.39, 132.04, 127.63, 125.87, 118.61, 118.31, 115.09, 111.91, 34.36, 31.72, 14.69, 8.10.

N-(6-(4-(3-(2-chlorophenyl)ureido)phenyl)pyridin-2-yl)cyclopropanecarboxamide(CDAU-16)

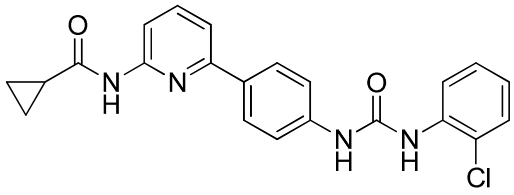

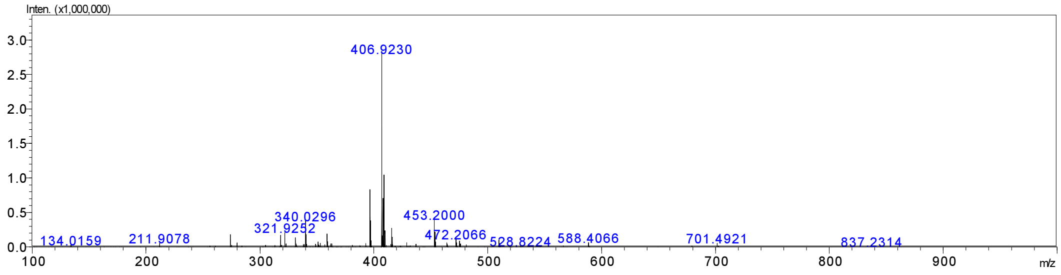

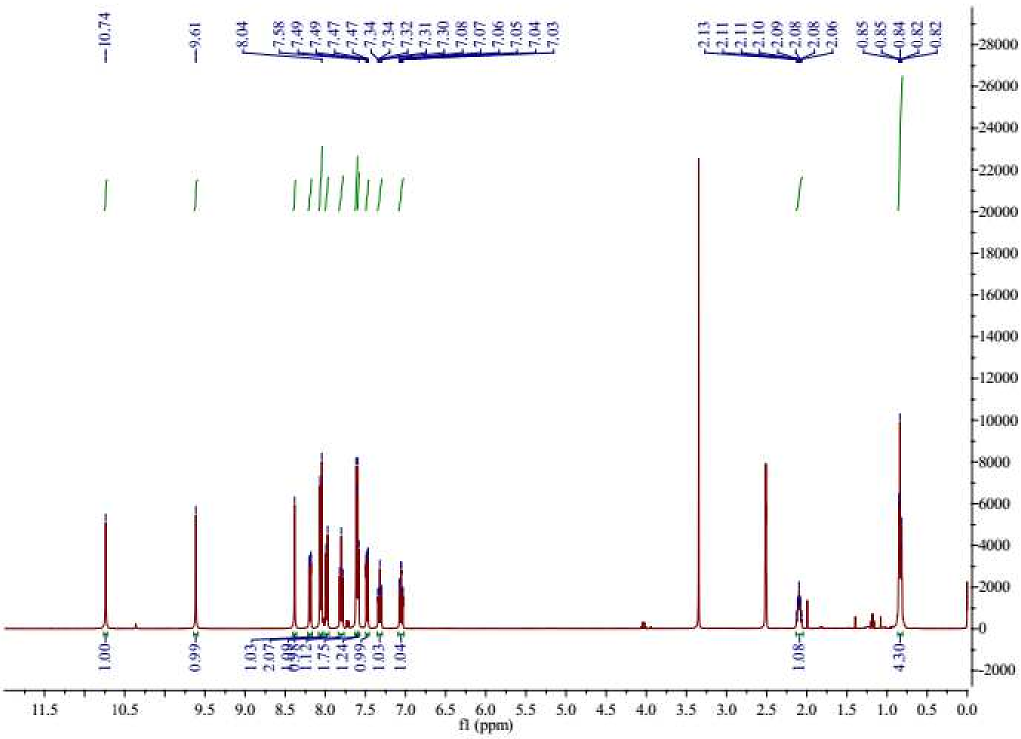

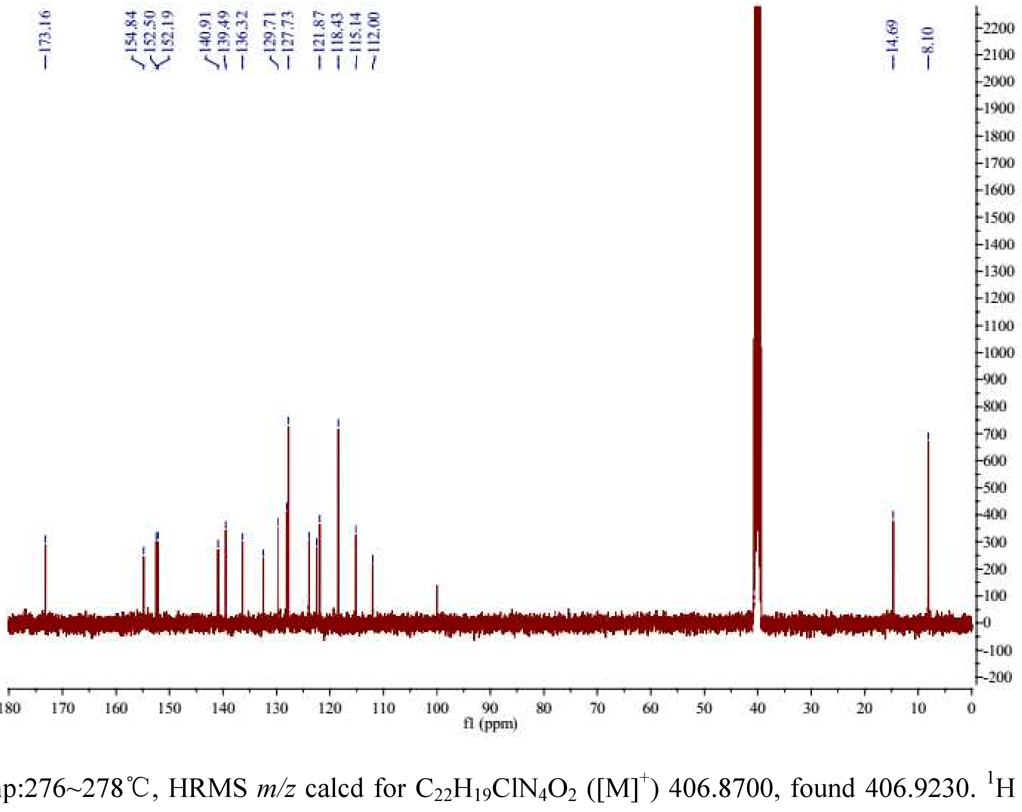

mp:276~278℃, HRMS *m/z* calcd for $C_{22}H_{19}ClN_4O_2$ ($[M]^+$) 406.8700, found 406.9230. ^1H NMR (400 MHz, DMSO-d_6) δ 10.74 (s, 1H), 9.61 (s, 1H), 8.38 (s, 1H), 8.19 (m, J = 8.3, 1.4 Hz, 1H), 8.05 (d, J = 8.7 Hz, 2H), 7.98 (d, J = 8.2 Hz, 1H), 7.80 (t, J = 7.9 Hz, 1H), 7.61 (d, J = 6.5 Hz, 2H), 7.59 (d, J = 5.0 Hz, 1H), 7.48 (m, J = 8.0, 1.4 Hz, 1H), 7.35 – 7.29 (m, 1H), 7.05 (td, J = 7.9, 1.5 Hz, 1H), 2.10 (dq, J = 7.6, 5.0 Hz, 1H), 0.87 – 0.81 (m, 4H).^{13}C NMR (101 MHz, DMSO-d_6) δ

173.16, 154.84, 152.50, 152.19, 140.91, 139.49, 136.32, 132.46, 129.71, 128.07, 127.73, 123.92, 122.52, 121.87, 118.43, 115.14, 112.00, 14.69, 8.10.

N-(6-(4-(3-(3-isopropylphenyl)ureido)phenyl)pyridin-2-yl)cyclopropanecarboxamide(CDAU-17)

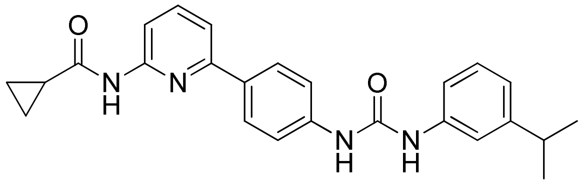

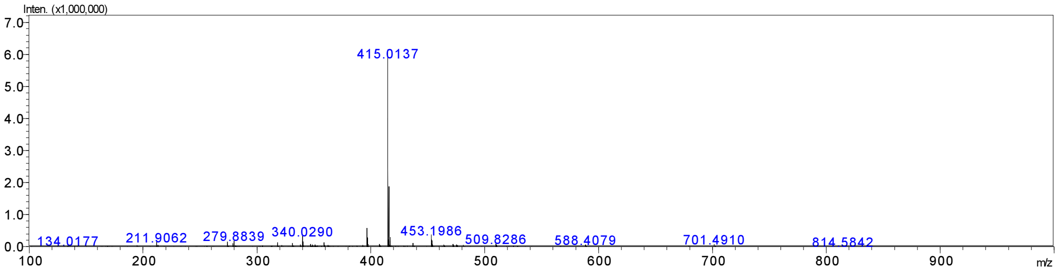

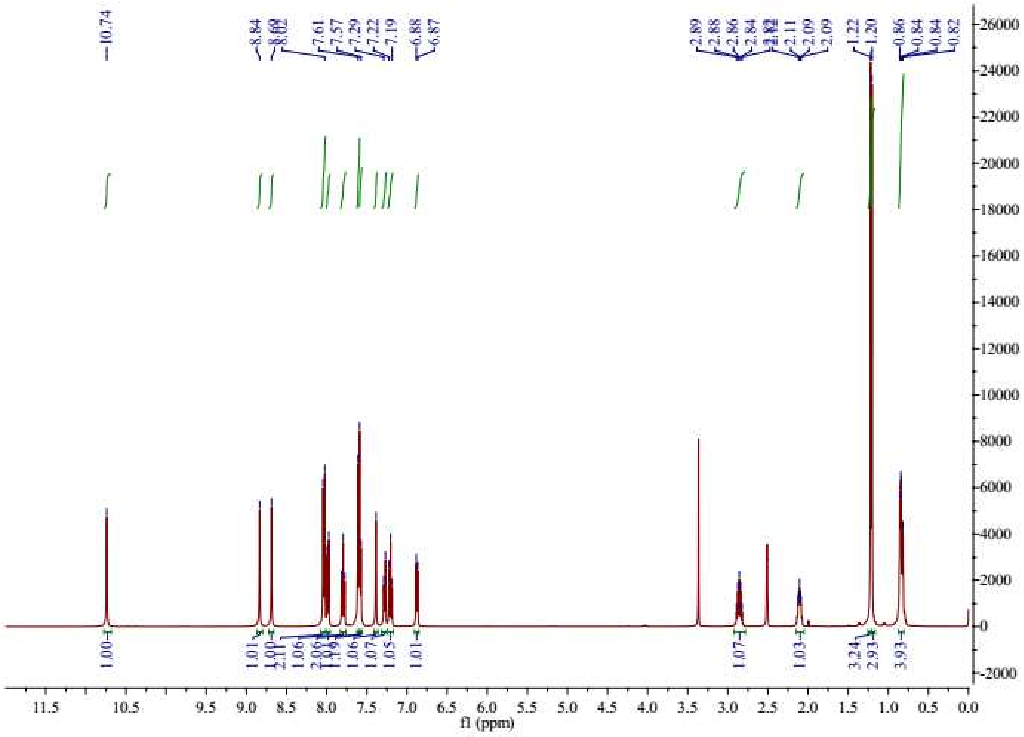

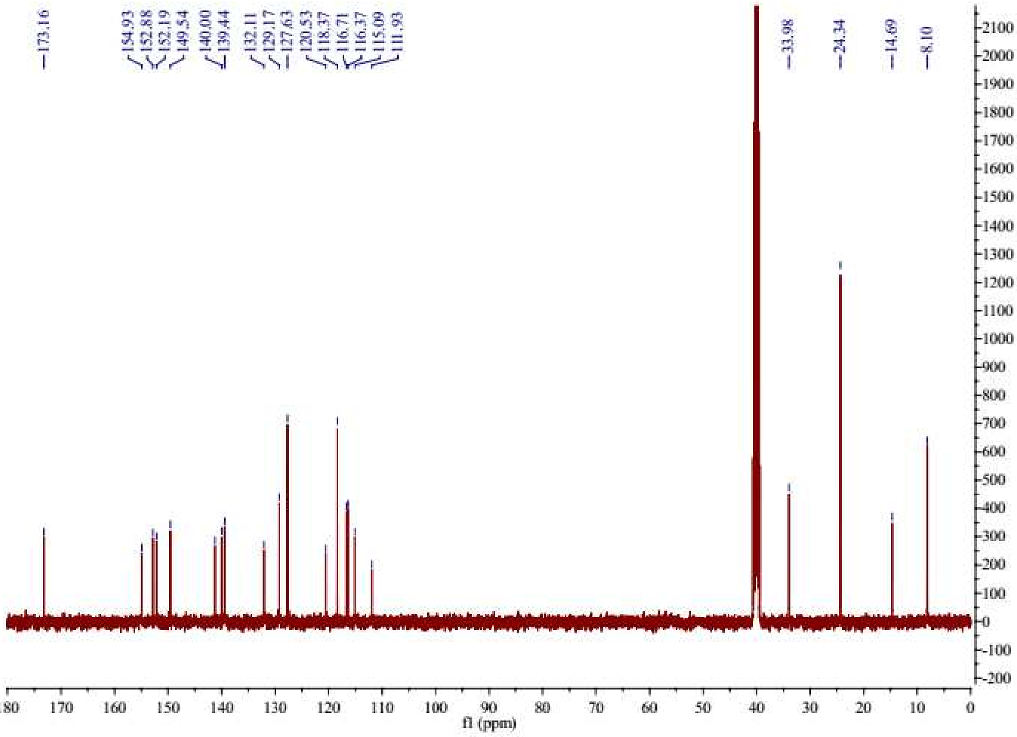

mp:207~209℃, HRMS *m/z* calcd for $C_{25}H_{26}N_4O_2$ ($[M+H]^+$) 415.2056, found 415.0137. ^{1}H NMR (400 MHz, DMSO-d_6) δ 10.74 (s, 1H), 8.84 (s, 1H), 8.69 (s, 1H), 8.03 (d, J = 8.7 Hz, 2H), 7.98 (d, J = 8.2 Hz, 1H), 7.79 (t, J = 7.9 Hz, 1H), 7.60 (d, J = 8.5 Hz, 2H), 7.58 (d, J = 6.8 Hz, 1H), 7.39 (s, 1H), 7.28 (d, J = 8.1 Hz, 1H), 7.21 (t, J = 7.8 Hz, 1H), 6.88 (d, J = 7.5 Hz, 1H), 2.93 – 2.78 (m, 1H), 2.15 – 2.04 (m, 1H), 1.22 (s, 3H), 1.20 (s, 3H), 0.84 (m, J = 8.5, 6.5 Hz, 4H).^{13}C NMR (101 MHz, DMSO-d_6) δ 173.16, 154.93, 152.88, 152.19, 149.54, 141.25, 140.00, 139.44, 132.11, 129.17, 127.63, 120.53, 118.37, 116.71, 116.37, 115.09, 111.93, 33.98, 24.34, 14.69, 8.10.

N-(6-(4-(3-(3-chlorophenyl)ureido)phenyl)pyridin-2-yl)cyclopropanecarboxamide(CDAU-18)

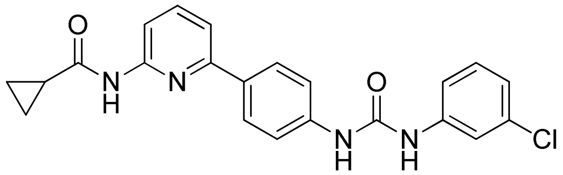

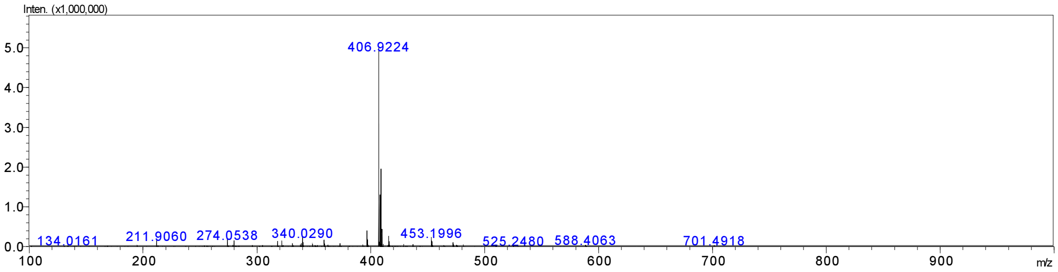

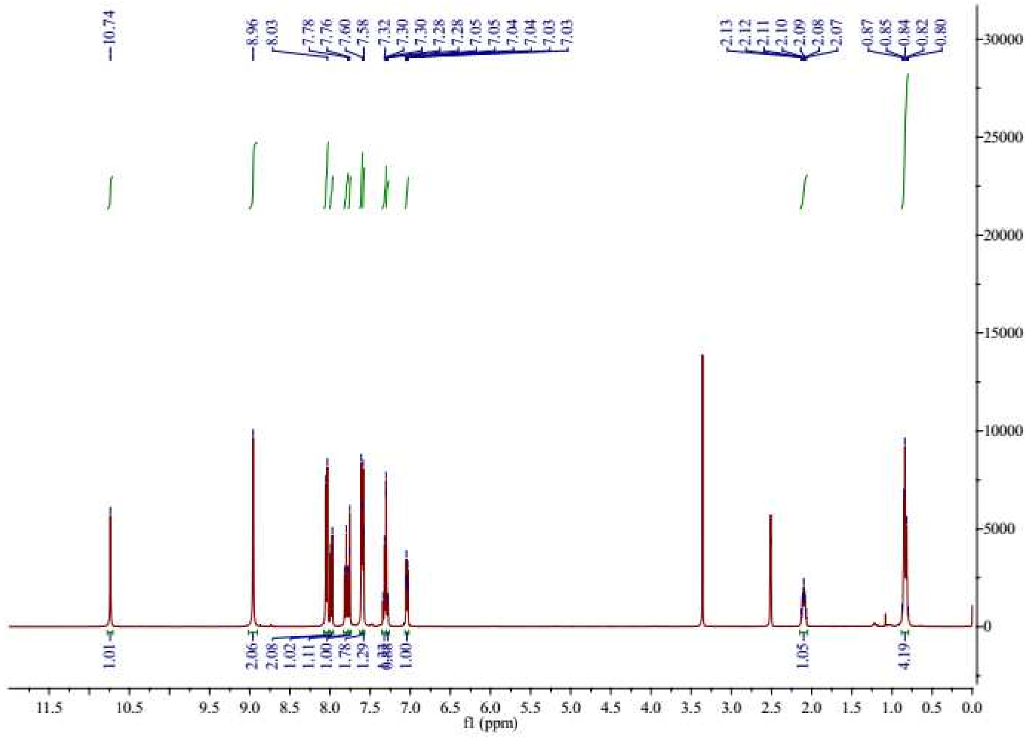

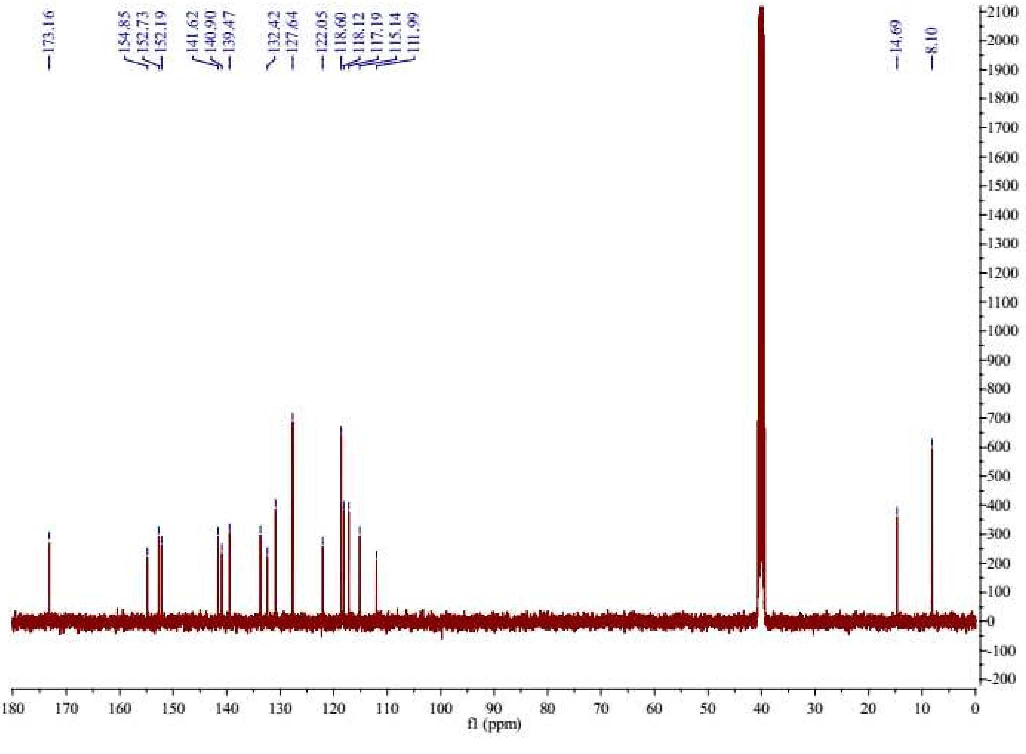

mp:216~218℃, HRMS *m/z* calcd for $C_{22}H_{19}ClN_4O_2$ ($[M]^+$) 406.8700, found 406.9224. ^1H NMR (400 MHz, DMSO-d_6) δ 10.74 (s, 1H), 8.96 (s, 2H), 8.04 (d, J = 8.7 Hz, 2H), 7.98 (d, J = 8.2 Hz, 1H), 7.80 (t, J = 7.9 Hz, 1H), 7.76 (d, J = 1.8 Hz, 1H), 7.60 (d, J = 5.0 Hz, 2H), 7.58 (d, J = 3.5 Hz, 1H), 7.35 – 7.30 (m, 1H), 7.30 – 7.27 (m, 1H), 7.04 (m, J = 7.3, 1.8 Hz, 1H), 2.15 – 2.06 (m, 1H), 0.88 – 0.80 (m, 4H).^{13}C NMR (101 MHz, DMSO-d_6) δ 173.16, 154.85, 152.73, 152.19, 141.62,

140.90, 139.47, 133.70, 132.42, 130.88, 127.64, 122.05, 118.60, 118.12, 117.19, 115.14, 111.99, 14.69, 8.10.

N-(6-(4-(3-(4-(trifluoromethoxy)phenyl)ureido)phenyl)pyridin-2-yl)cyclopropanecarboxamide(CDAU-19)

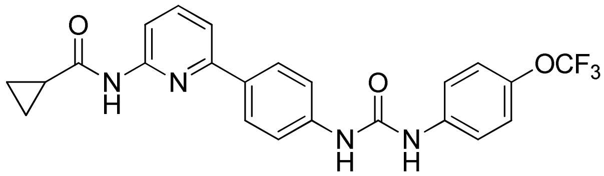

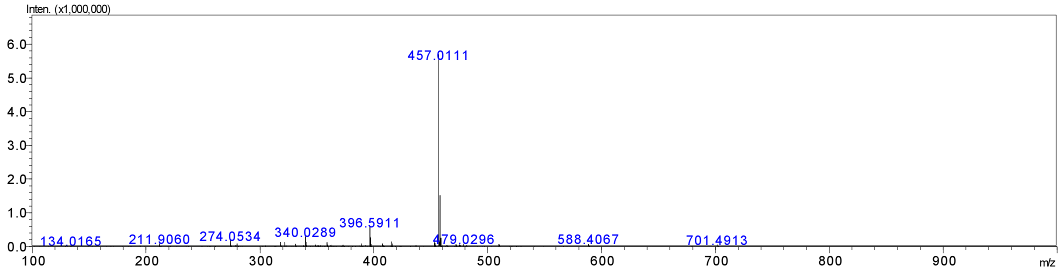

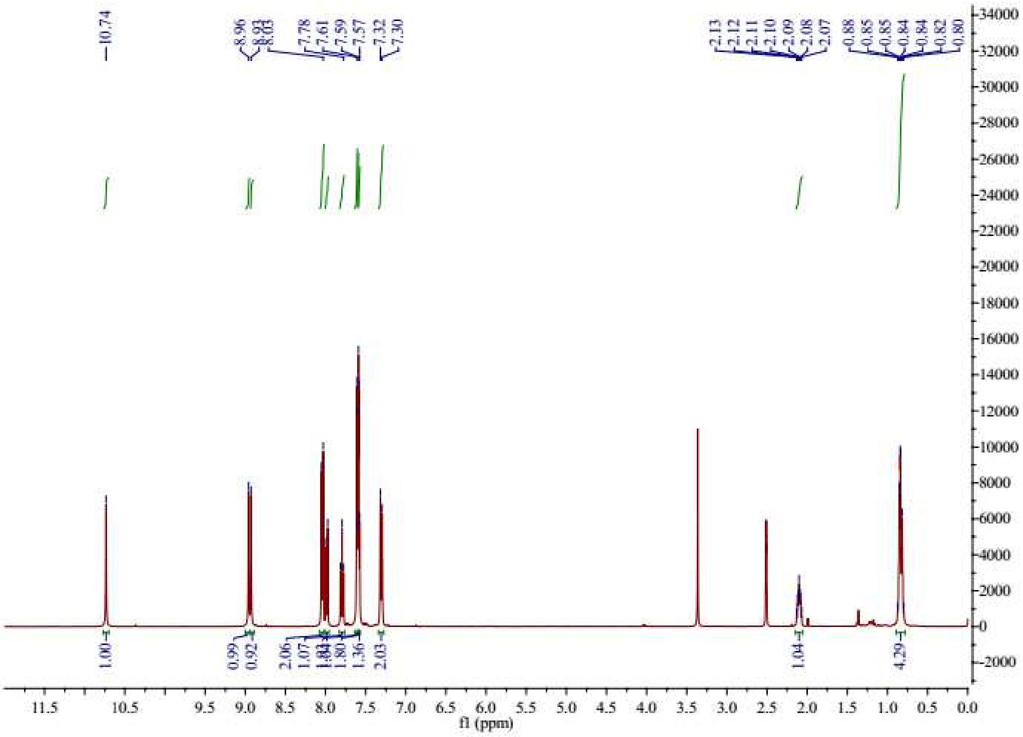

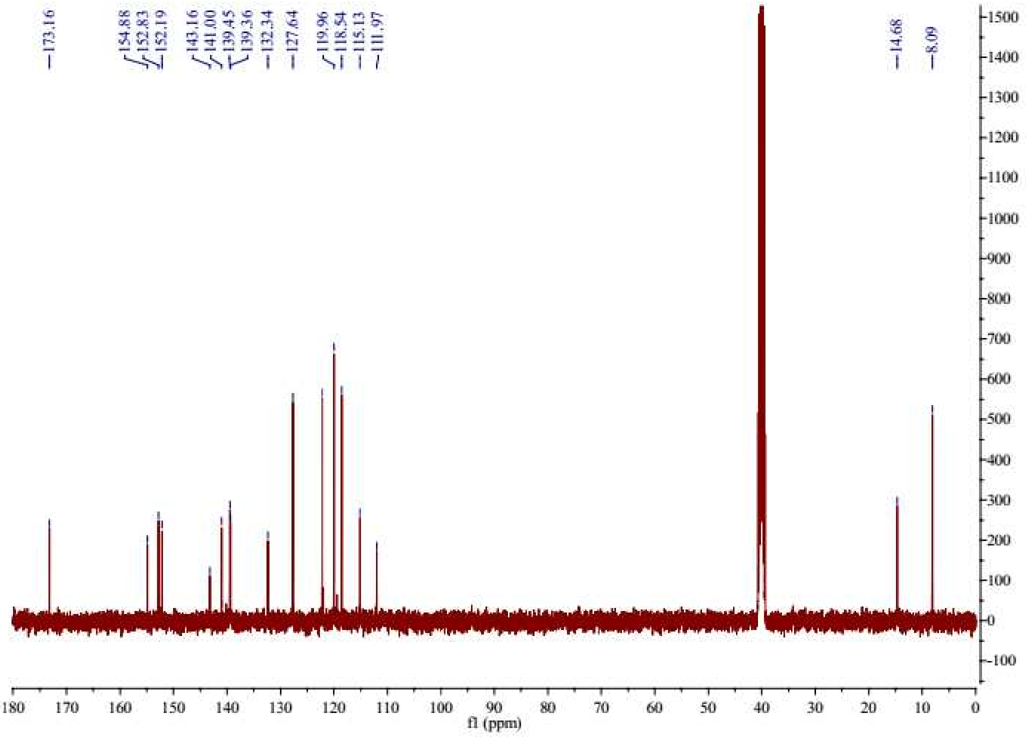

mp:276~278℃, HRMS *m/z* calcd for $C_{23}H_{19}F_3N_4O_3$ ([M]$^+$) 456.1409, found 457.0111. ^{1}H NMR (400 MHz, DMSO-d_6) δ 10.74 (s, 1H), 8.96 (s, 1H), 8.93 (s, 1H), 8.04 (d, *J* = 8.7 Hz, 2H), 7.98 (d, *J* = 8.2 Hz, 1H), 7.80 (t, *J* = 7.9 Hz, 1H), 7.61 (d, *J* = 1.8 Hz, 2H), 7.59 (d, *J* = 4.7 Hz, 2H), 7.58 (d, *J* = 3.9 Hz, 1H), 7.31 (d, *J* = 8.6 Hz, 2H), 2.14 – 2.05 (m, 1H), 0.89 – 0.78 (m, 4H). ^{13}C NMR (101 MHz, DMSO-d_6) δ 173.16, 154.88, 152.83, 152.19, 143.16, 141.00, 139.45, 139.36, 132.34, 127.64, 122.20, 119.96, 118.54, 115.13, 111.97, 14.68, 8.09.

N-(6-(4-(3-(2,4-dichlorophenyl)ureido)phenyl)pyridin-2-yl)cyclopropanecarboxamide(CDAU-20)

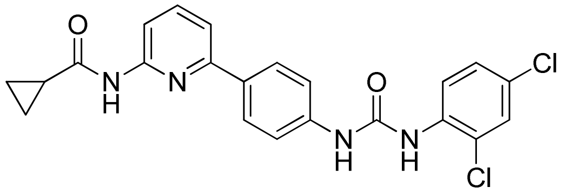

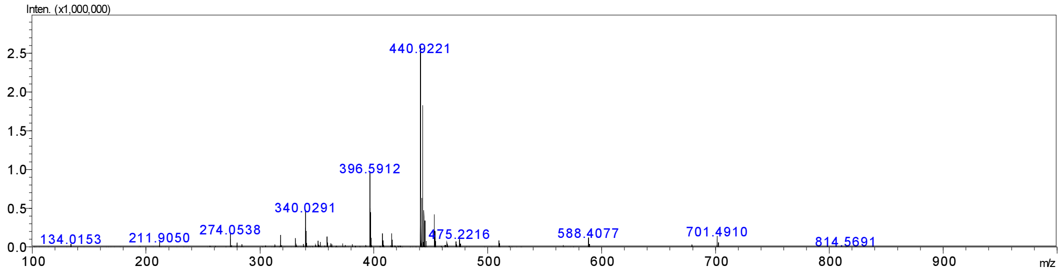

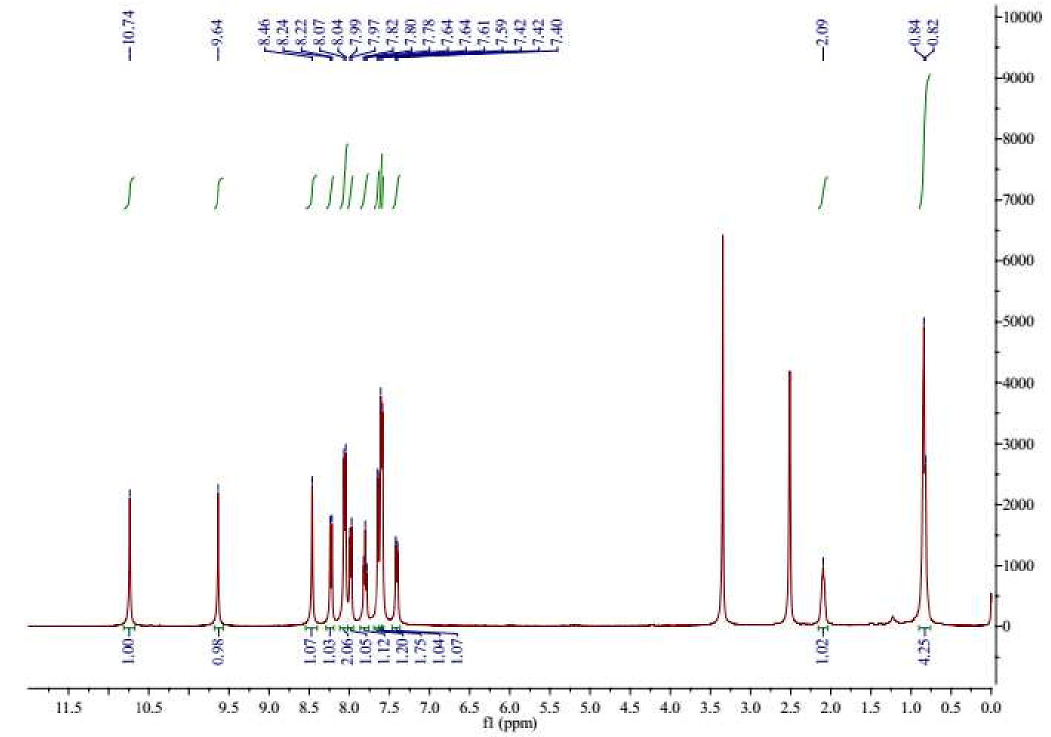

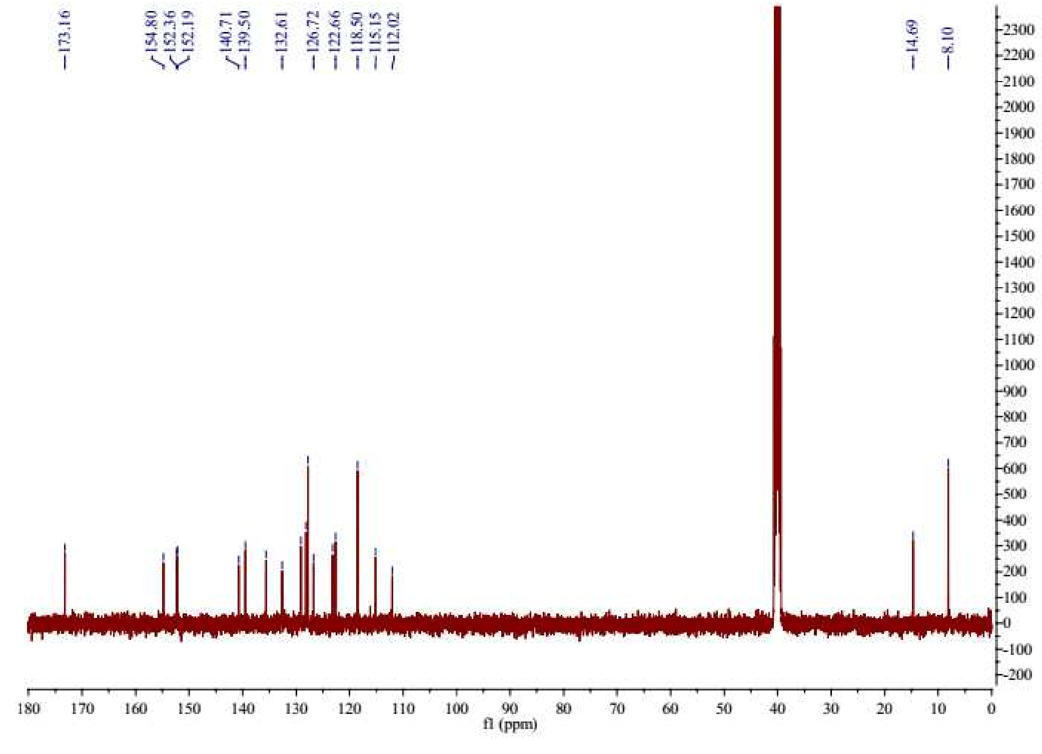

mp:222~224℃, HRMS *m/z* calcd for $C_{22}H_{18}Cl_2N_4O_2$ ($[M]^+$) 440.0807, found 440.9221. ^{1}H NMR (400 MHz, DMSO-d_6) δ 10.74 (s, 1H), 9.64 (s, 1H), 8.46 (s, 1H), 8.23 (d, J = 8.9 Hz, 1H), 8.06 (d, J = 8.5 Hz, 2H), 7.98 (d, J = 8.1 Hz, 1H), 7.80 (t, J = 7.9 Hz, 1H), 7.64 (d, J = 2.2 Hz, 1H), 7.61 (s, 2H), 7.59 (s, 1H), 7.47 – 7.37 (m, 1H), 2.09 (s, 1H), 0.83 (d, J = 7.7 Hz, 4H).^{13}C NMR (101 MHz, DMSO-d_6) δ 173.16, 154.80, 152.36, 152.19, 140.71, 139.50, 135.58, 132.61, 129.06, 128.13, 127.74, 126.72, 123.20, 122.66, 118.50, 115.15, 112.02, 14.69, 8.10.

N-(6-(4-(3-(3-(trifluoromethyl)phenyl)ureido)phenyl)pyridin-2-yl)cyclopropanecarboxamide(CDAU-21)

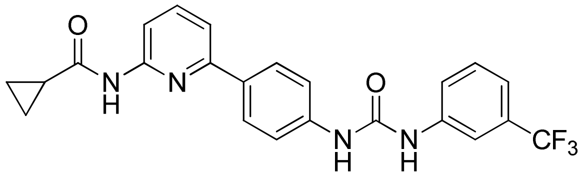

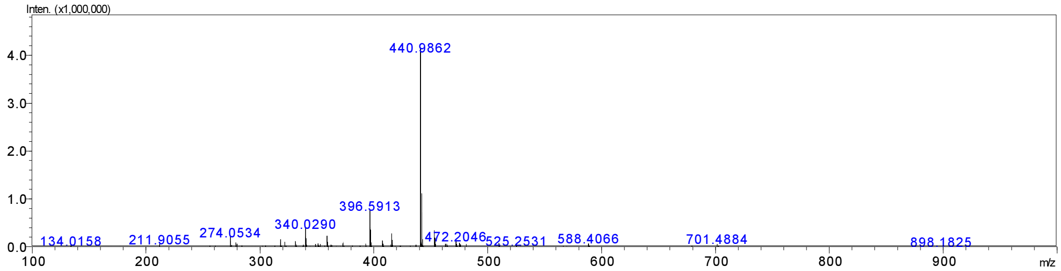

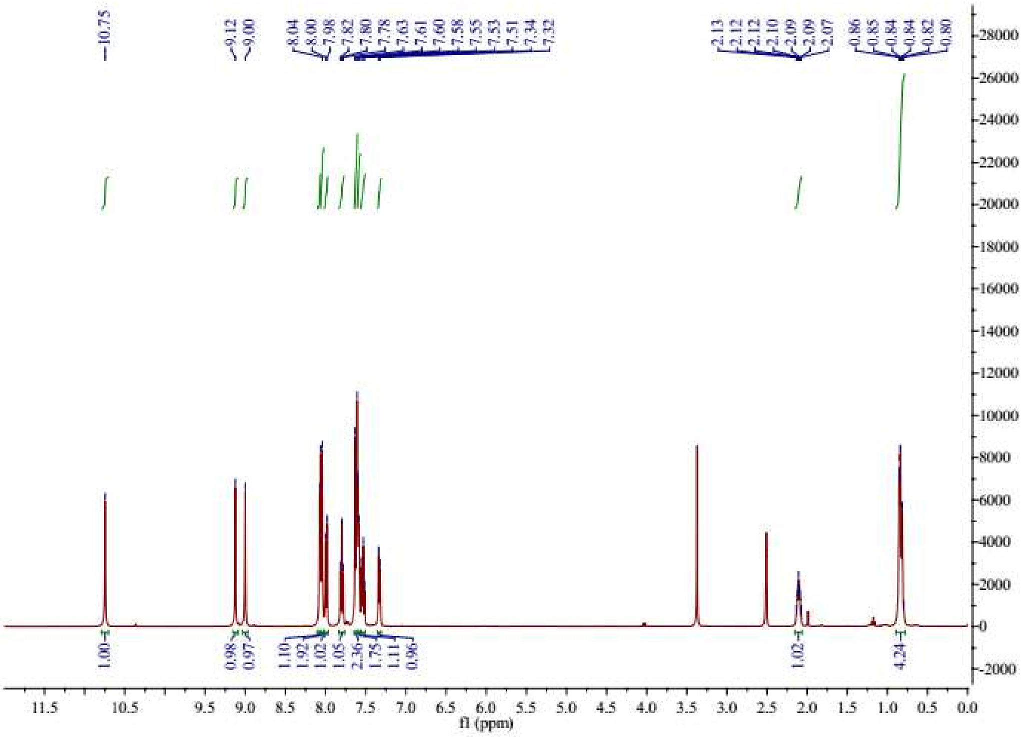

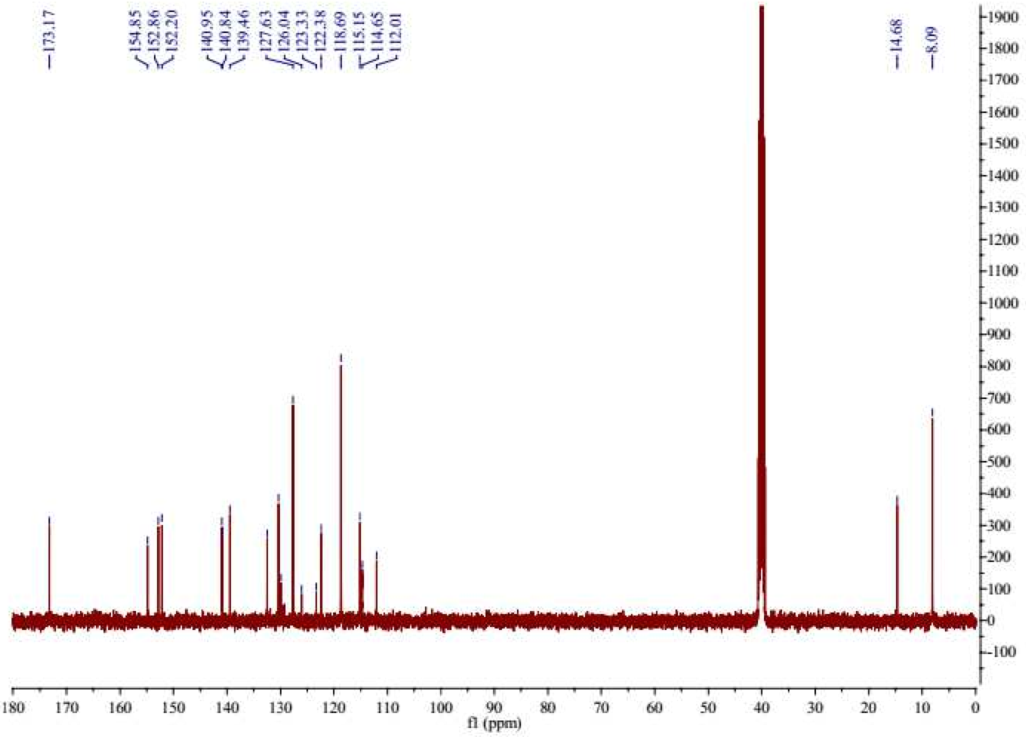

mp:275~277℃, HRMS *m/z* calcd for $C_{23}H_{19}F_3N_4O_2$ ($[M]^+$) 440.1460, found 440.9862. ^{1}H NMR (400 MHz, DMSO-d_6) δ 10.75 (s, 1H), 9.12 (s, 1H), 9.00 (s, 1H), 8.07 (s, 1H), 8.05 (d, *J* = 8.8 Hz, 2H), 7.99 (d, *J* = 8.2 Hz, 1H), 7.80 (t, *J* = 7.9 Hz, 1H), 7.62 (d, *J* = 8.7 Hz, 2H), 7.59 (d, *J* = 7.6 Hz, 2H), 7.53 (t, *J* = 7.9 Hz, 1H), 7.33 (d, *J* = 7.5 Hz, 1H), 2.15 – 2.06 (m, 1H), 0.83 (m, *J* = 16.0, 4.7 Hz, 4H).^{13}C NMR (101 MHz, DMSO-d_6) δ 173.17, 154.85, 152.86, 152.20, 140.95, 140.84, 139.46, 132.49, 130.39, 129.88, 127.63, 126.04, 123.33, 122.38, 118.69, 115.15, 114.65, 112.01, 14.68, 8.09.

N-(6-(4-(3-(2,4-difluorophenyl)ureido)phenyl)pyridin-2-yl)cyclopropanecarboxamide(CDAU-22)

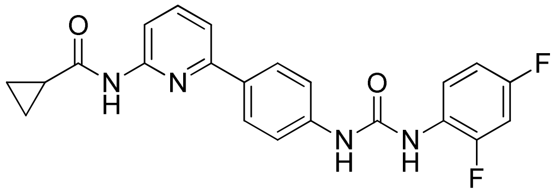

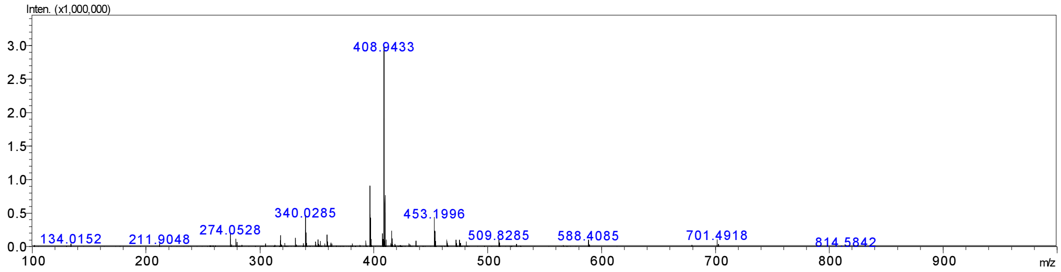

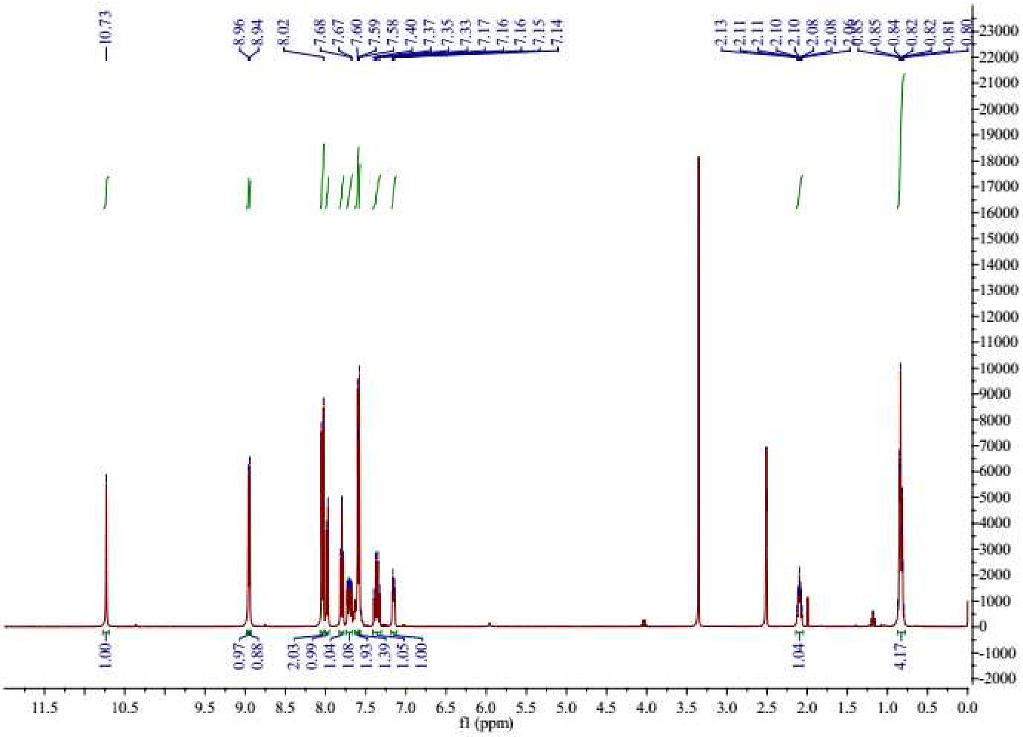

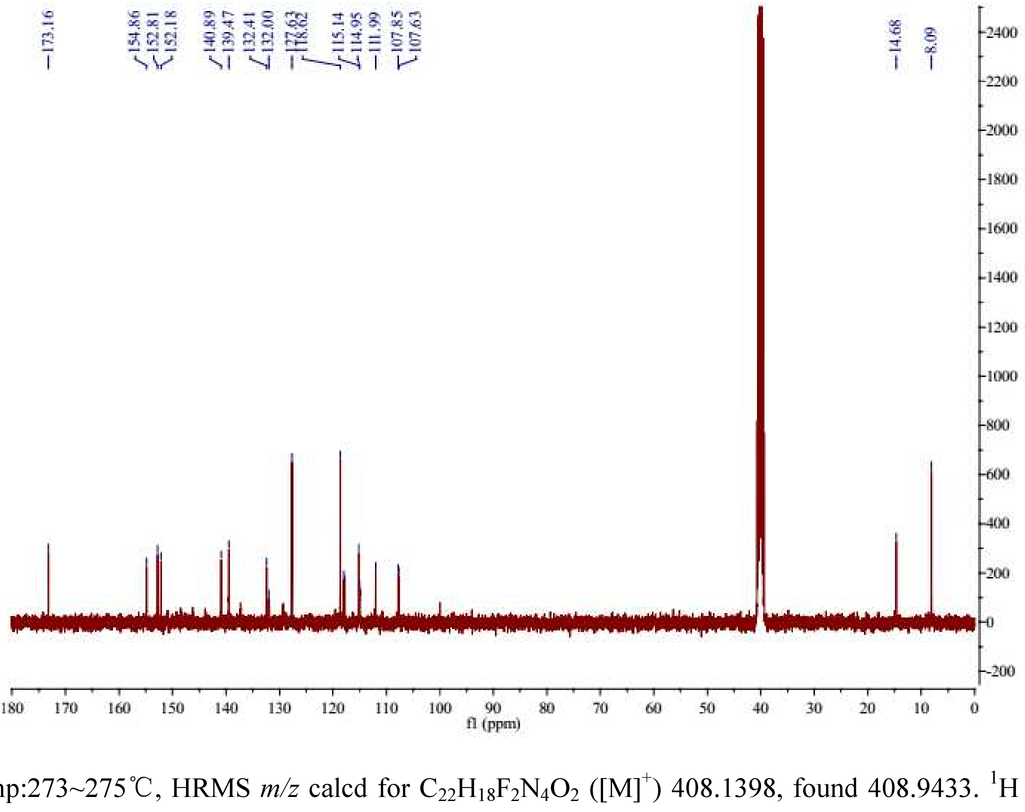

mp:273~275℃, HRMS *m/z* calcd for $C_{22}H_{18}F_2N_4O_2$ ($[M]^+$) 408.1398, found 408.9433. ^1H NMR (400 MHz, DMSO-d_6) δ 10.73 (s, 1H), 8.96 (s, 1H), 8.94 (s, 1H), 8.04 (d, *J* = 8.8 Hz, 2H), 7.98 (d, *J* = 8.2 Hz, 1H), 7.80 (t, *J* = 8.0 Hz, 1H), 7.70 (md, *J* = 13.4, 7.4, 2.6 Hz, 1H), 7.60 (d, *J* = 2.1 Hz, 2H), 7.58 (s, 1H), 7.36 (m, *J* = 19.7, 9.2 Hz, 1H), 7.18 – 7.12 (m, 1H), 2.10 (m, *J* = 7.5, 5.0 Hz, 1H), 0.89 – 0.76 (m, 4H).^{13}C NMR (101 MHz, DMSO-d_6) δ 173.16, 154.86, 152.81, 152.18,

140.89, 139.47, 132.41, 132.00, 127.63, 118.62, 117.96, 117.77, 115.14, 114.95, 111.99, 107.85, 107.63, 14.68, 8.09.

N-(6-(4-(3-(2-fluorophenyl)ureido)phenyl)pyridin-2-yl)cyclopropanecarboxamide(CDAU-23)

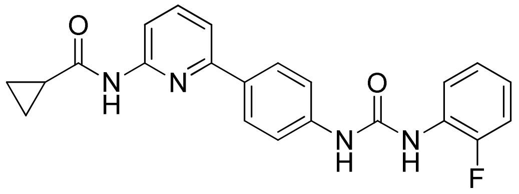

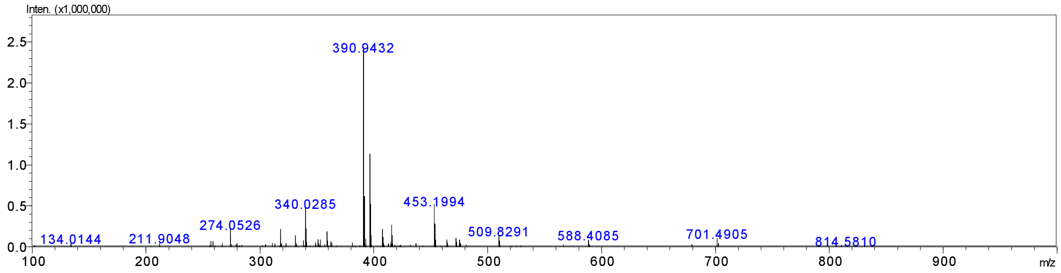

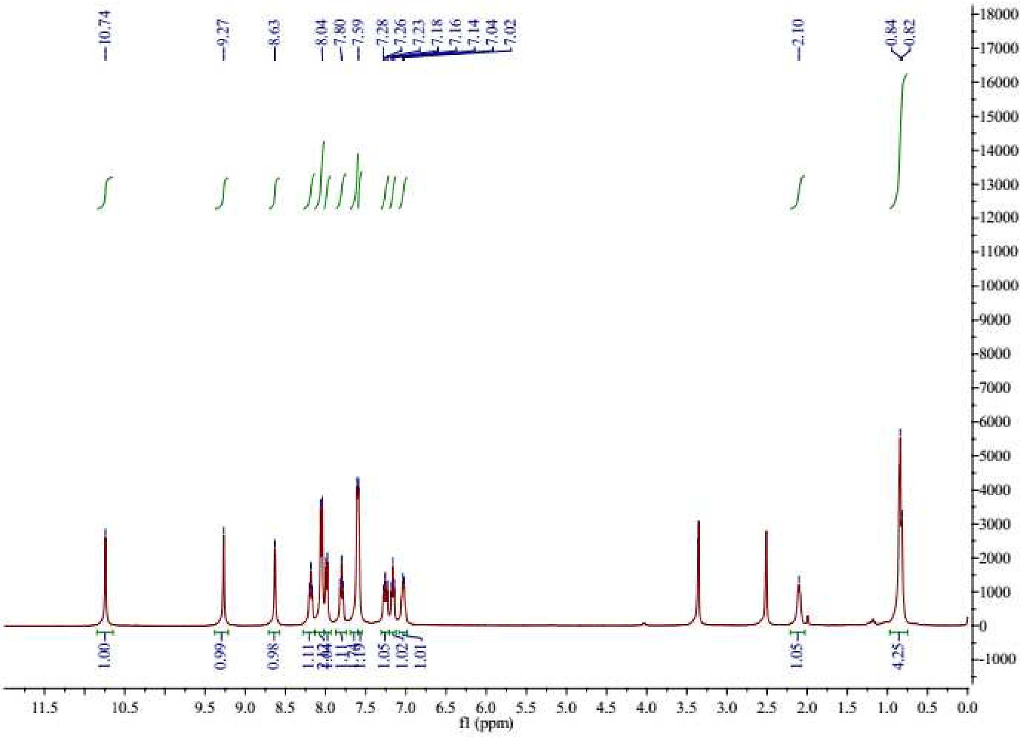

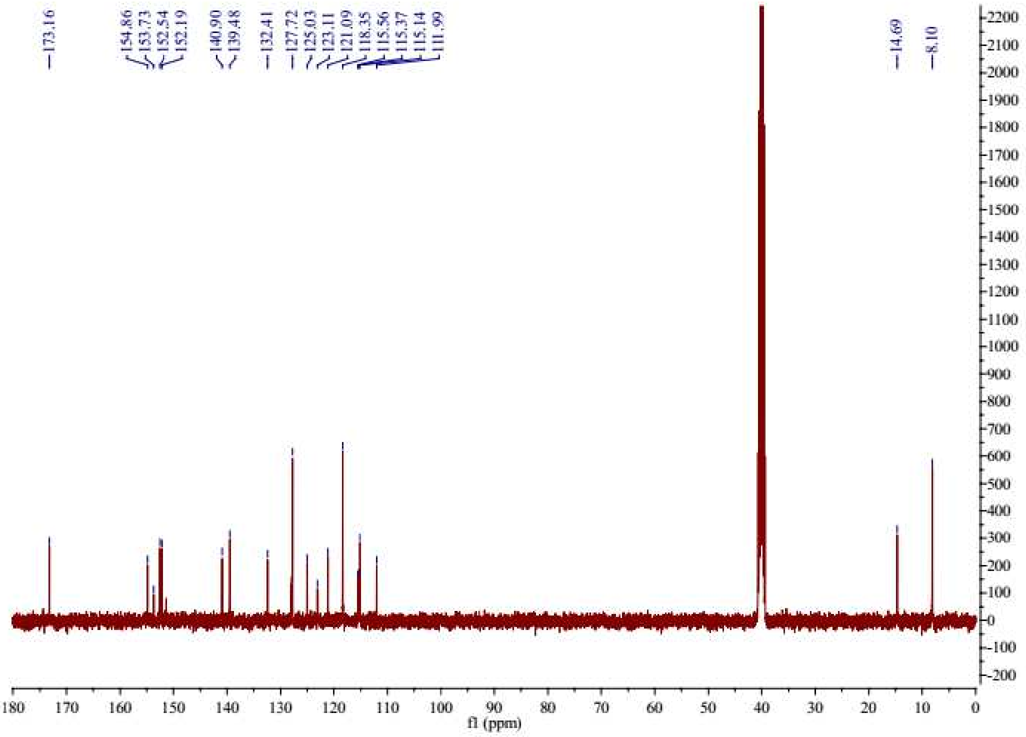

mp:239~241℃, HRMS *m/z* calcd for $C_{22}H_{19}FN_4O_2$ ($[M+H]^+$) 390.1492, found 390.9432. ^1H NMR (400 MHz, DMSO-d_6) δ 10.74 (s, 1H), 9.27 (s, 1H), 8.63 (s, 1H), 8.18 (t, *J* = 7.9 Hz, 1H), 8.05 (d, *J* = 8.4 Hz, 2H), 7.98 (d, *J* = 8.0 Hz, 1H), 7.80 (t, *J* = 7.8 Hz, 1H), 7.60 (d, *J* = 3.8 Hz, 2H), 7.59 (s, 1H), 7.31 – 7.21 (m, 1H), 7.16 (t, *J* = 7.5 Hz, 1H), 7.03 (d, *J* = 5.5 Hz, 1H), 2.10 (s, 1H), 0.83 (d, *J* = 8.0 Hz, 4H).^{13}C NMR (101 MHz, DMSO-d_6) δ 173.16, 154.86, 153.73, 152.54, 152.19, 140.90, 139.48, 132.41, 127.72, 125.03, 123.11, 121.09, 118.35, 115.56, 115.37, 115.14, 111.99, 14.69, 8.10.

N-(6-(4-(3-(3-fluorophenyl)ureido)phenyl)pyridin-2-yl)cyclopropanecarboxamide(CDAU-24)

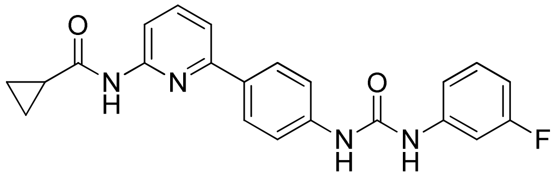

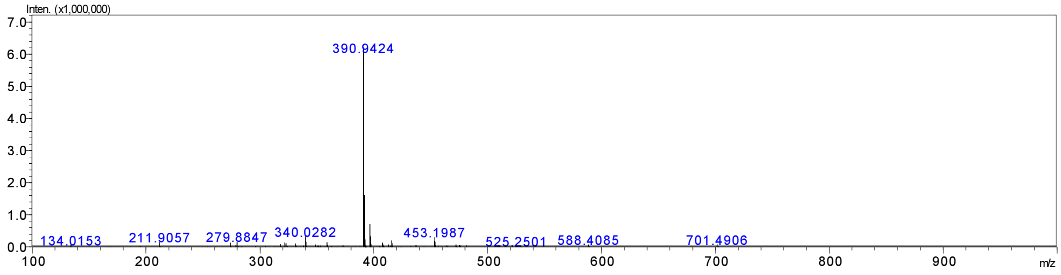

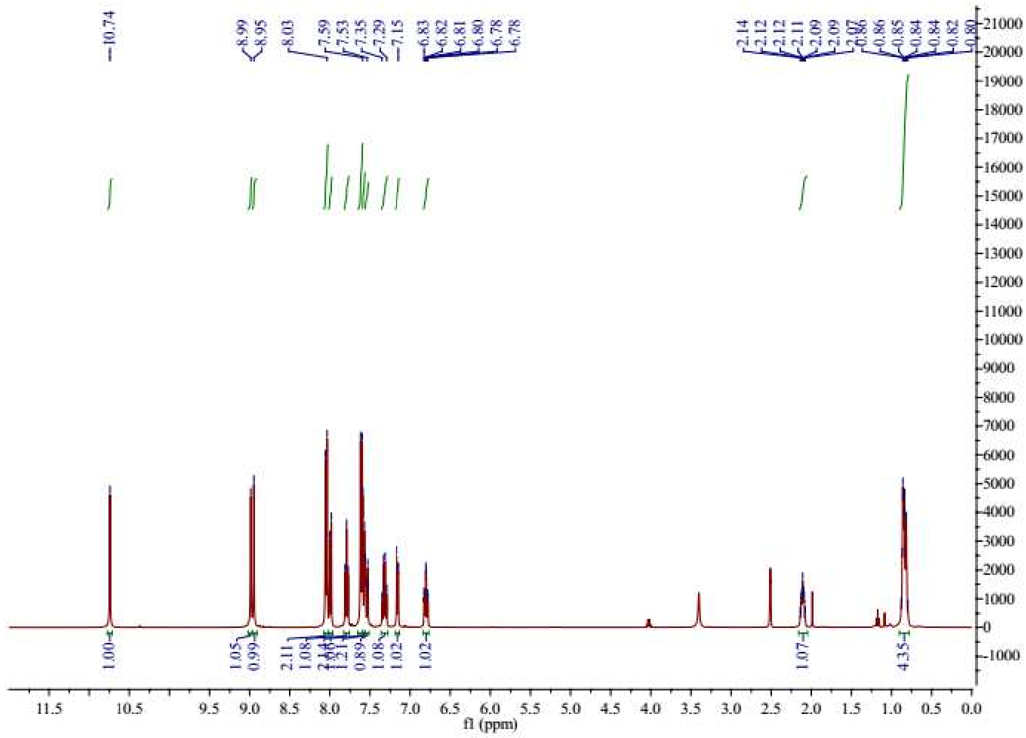

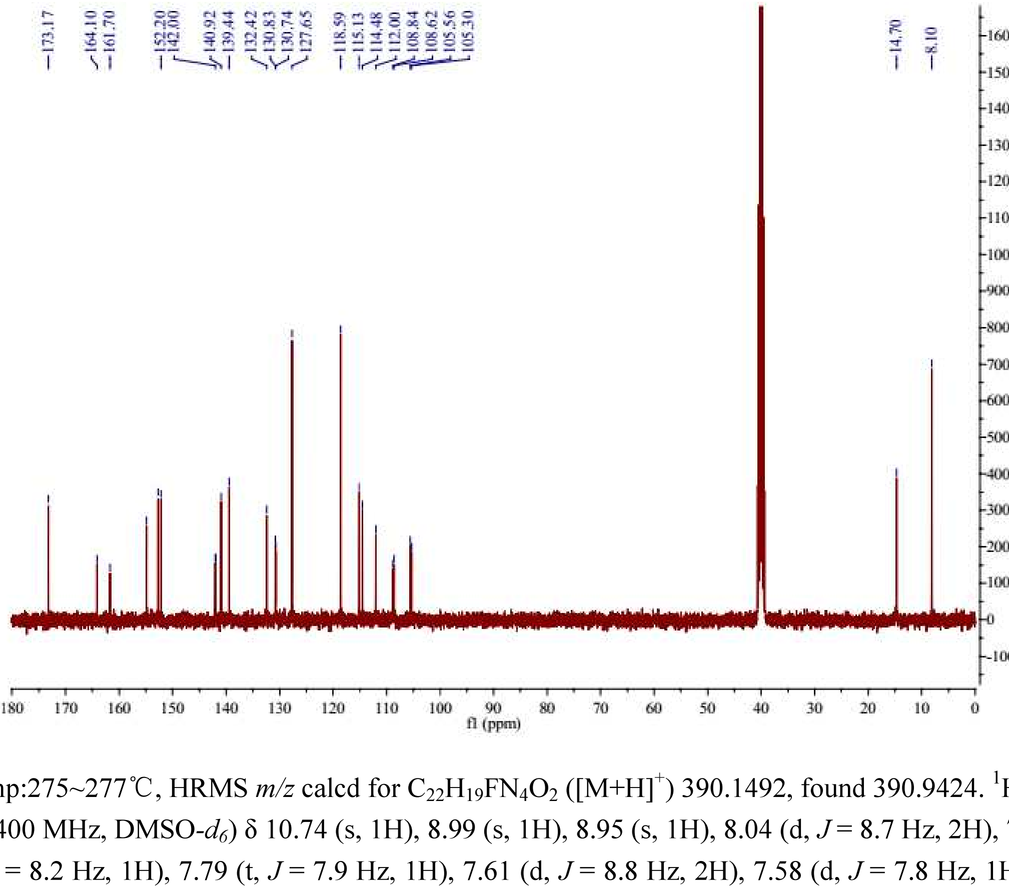

mp:275~277℃, HRMS *m/z* calcd for $C_{22}H_{19}FN_4O_2$ ($[M+H]^+$) 390.1492, found 390.9424. ^1H NMR (400 MHz, DMSO-d_6) δ 10.74 (s, 1H), 8.99 (s, 1H), 8.95 (s, 1H), 8.04 (d, *J* = 8.7 Hz, 2H), 7.99 (d, *J* = 8.2 Hz, 1H), 7.79 (t, *J* = 7.9 Hz, 1H), 7.61 (d, *J* = 8.8 Hz, 2H), 7.58 (d, *J* = 7.8 Hz, 1H), 7.54 (m, *J* = 8.0, 6.0 Hz, 1H), 7.32 (m, *J* = 15.2, 8.1 Hz, 1H), 7.18 – 7.13 (m, 1H), 6.80 (t, *J* = 8.4, 2.2 Hz, 1H), 2.18 – 2.05 (m, 1H), 0.90 – 0.78 (m, 4H).^{13}C NMR (101 MHz, DMSO-d_6) δ 173.17,

164.10, 161.70, 154.88, 152.74, 152.20, 142.00, 141.89, 140.92, 139.44, 132.42, 130.83, 130.74, 127.65, 118.59, 115.13, 114.48, 112.00, 108.84, 108.62, 105.56, 105.30, 14.70, 8.10.

N-(6-(4-(3-(2,6-dimethylphenyl)ureido)phenyl)pyridin-2-yl)cyclopropanecarboxamide(CDAU-25)

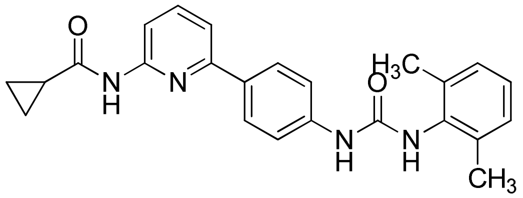

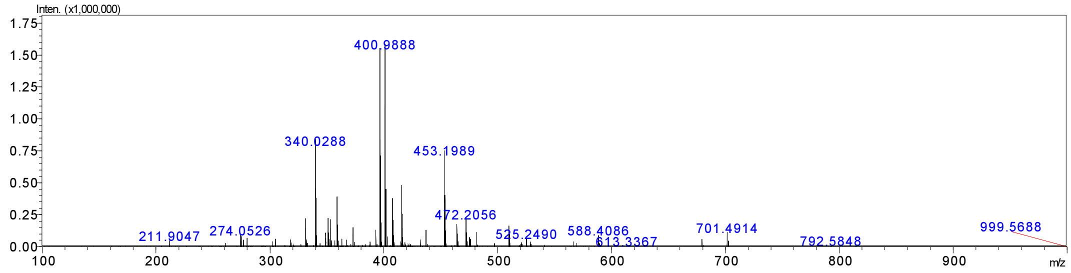

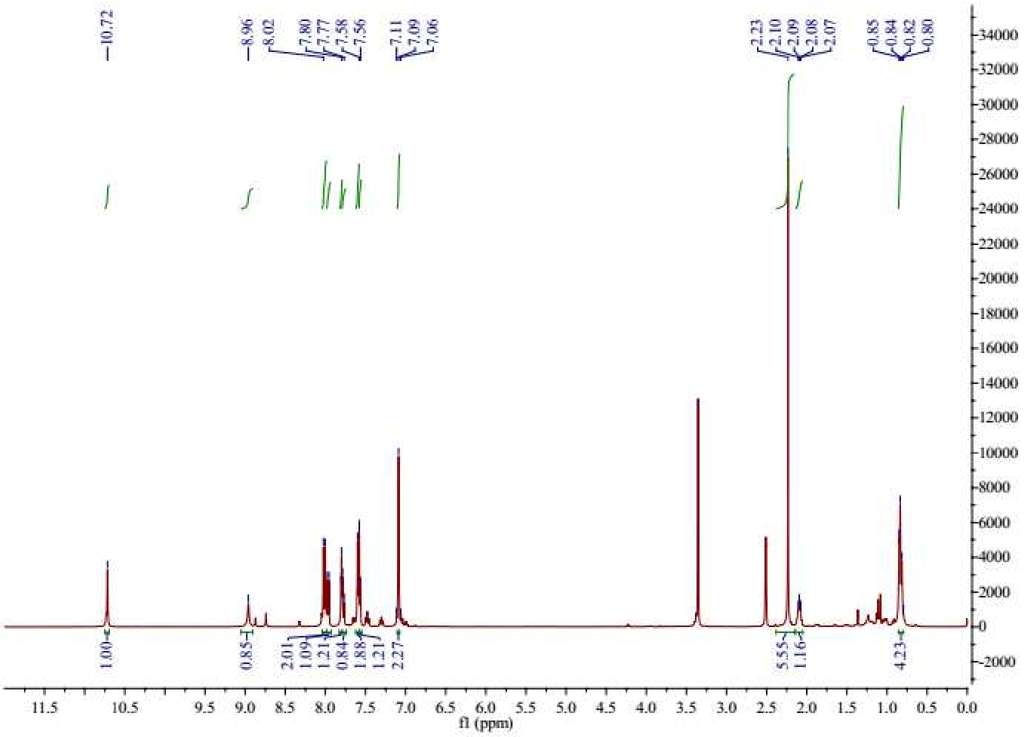

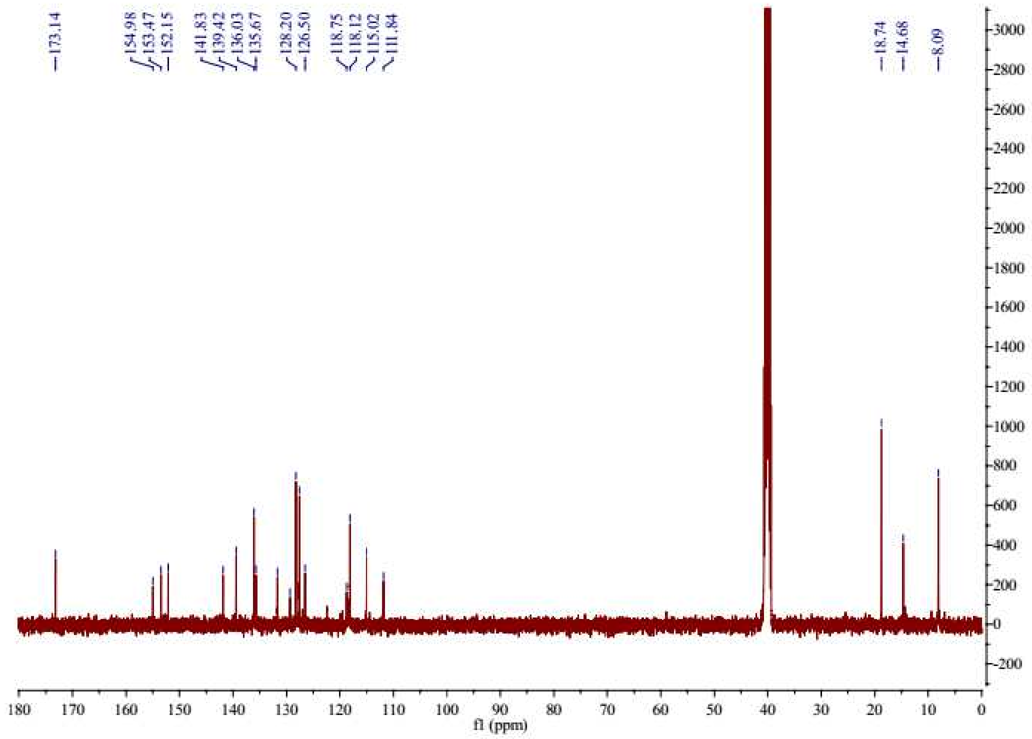

mp:202~204℃, HRMS *m/z* calcd for $C_{24}H_{24}N_4O_2$ ($[M]^+$) 400.1899, found 400.9888. ^{1}H NMR (400 MHz, DMSO-*d*$_6$) δ 10.72 (s, 1H), 8.96 (s, 1H), 8.01 (d, *J* = 8.8 Hz, 2H), 7.97 (d, *J* = 8.0 Hz, 1H), 7.80 (d, *J* = 2.8 Hz, 1H), 7.78 (d, *J* = 8.0 Hz, 1H), 7.59 (d, *J* = 7.9 Hz, 2H), 7.57 (d, *J* = 6.3 Hz, 1H), 7.12 – 7.06 (m,2H), 2.23 (s,6H), 2.14 – 2.05 (m, 1H), 0.83 (m, *J* = 14.8, 6.2 Hz, 4H).^{13}C NMR (101 MHz, DMSO-*d*$_6$) δ 173.14, 154.98, 153.47, 152.15, 141.83, 139.42, 136.03, 135.67, 131.70, 129.28, 128.20, 127.56, 126.50, 118.75, 118.12, 115.02, 111.84, 18.74, 14.68, 8.09.
